# A meta-analysis of the long-term effects of antihypertensive therapy on the risk of major cardiovascular disease across 51 randomized trials

**DOI:** 10.1038/s41591-026-04514-3

**Published:** 2026-07-01

**Authors:** Qianqian Yang, Zeinab Bidel, Dexter Canoy, Guyu Zeng, William C. Cushman, Anthony Rodgers, Koon Teo, Barry R. Davis, John Chalmers, Carl J. Pepine, Johan Sundström, Mark Woodward, Milad Nazarzadeh, Kazem Rahimi

**Affiliations:** 1 Deep Medicine, Nuffield Department of Women’s and Reproductive Health, University of Oxford, Oxford, UK; 2 Population Health Sciences Institute, Newcastle University, Newcastle, UK; 3 University of Tennessee Health Science Center, Memphis, TN USA; 4 The George Institute for Global Health, University of New South Wales, Sydney, New South Wales Australia; 5 Population Health Research Institute, Hamilton Health Sciences, McMaster University, Hamilton, Ontario Canada; 6 The University of Texas School of Public Health, Houston, TX USA; 7 College of Medicine, University of Florida, Gainesville, FL USA; 8 Department of Medical Sciences, Clinical Epidemiology, Uppsala University, Uppsala, Sweden; 9 The George Institute for Global Health, School of Public Health, Imperial College London, London, UK

**Keywords:** Hypertension, Risk factors, Epidemiology

## Abstract

Blood pressure (BP)-lowering therapy reduces cardiovascular risk, but whether its proportional benefits increase with longer treatment duration remains unclear. We conducted an individual participant-level data meta-analysis of 51 randomized trials from the Blood Pressure Lowering Treatment Trialistsʼ Collaboration (358,642 participants; median follow-up: 4.2 years). Using Cox proportional hazards models, we estimated time-stratified hazard ratios (HRs) for major cardiovascular events (MACE; fatal or non-fatal stroke, ischemic heart disease or heart failure) across annual follow-up intervals up to more than 5 years, standardized to a 5-mmHg systolic BP reduction. Network meta-analysis examined whether temporal patterns differed across antihypertensive drug classes. Annual MACE incidence was highest during year 1 (3.0% treatment versus 3.6% control), declined during years 1−5 and then rose at more than 5 years (3.1% versus 3.4%). BP lowering reduced MACE risk, with benefits established early and not progressively increasing over time. A 5-mmHg systolic BP reduction was associated with a 12% lower MACE risk in year 1 (HR = 0.88, 95% confidence interval (CI): 0.84−0.91), with modest attenuation thereafter: HRs were 0.88 (0.85−0.92) in years 1−2, 0.94 (0.90−0.98) in years 2−3, 0.87 (0.83−0.92) in years 3−4, 0.97 (0.91–1.03) in years 4−5 and 0.94 (0.87−1.01) at more than 5 years (*P* for trend = 0.006). Similar patterns occurred across five drug classes. These findings indicate that the relative cardiovascular benefits of BP lowering emerge within months and do not increase over time, suggesting that prioritizing higher-risk individuals for treatment yields greater clinical utility than prolonged treatment in low-risk individuals.

## Abstract (web summary)

Results from an individual participant data meta-analysis across 51 randomized trials challenge the assumption that the cardiovascular benefits from antihypertensive treatment compound over a prolonged duration of treatment.

## Main

With growing emphasis on lifecourse cardiovascular risk management^1^, BP lowering has become central to discussions of earlier intervention^2,3^. Observational studies have shown that cumulative BP burden, thought to reflect progressive vascular and cardiac damage^4^, is strongly associated with cardiovascular disease^5,6^. In parallel, long-term follow-up of randomized trials has reported so-called ‘legacy effects’^7^, whereby early BP-lowering treatment is associated with persistent cardiovascular protection beyond the trial period.

Mendelian randomization studies, often considered proxies for lifelong BP exposure, have further reinforced these observations, showing substantially larger relative reductions in cardiovascular risk associated with genetically lower BP than those observed in short-term pharmacological trials^8–10^. Together, this evidence has motivated the hypothesis that longer BP-lowering treatment duration could yield progressively greater relative cardiovascular benefit^10^.

Similar time-dependent patterns have also been observed in lipid-lowering treatment^11,12^, where statin trials demonstrate modest early effects followed by larger proportional risk reductions with longer follow-up. By analogy, these findings have informed expectations of progressively increasing relative benefits with longer BP-lowering treatment.

On this basis, earlier and more proactive BP-lowering strategies have been proposed, including age-specific treatment thresholds^13^ and broader lifecourse prevention paradigms. Such approaches have been framed metaphorically as ‘vaccine-like’^14^, in the sense that BP-lowering and lipid-lowering interventions initiated earlier in life may confer long-term cardiovascular protection, on the premise that earlier intervention may yield disproportionately larger relative risk reductions over the lifecourse^15^.

However, no randomized evidence has directly tested whether BP-lowering therapy itself exhibits a similar time-dependent amplification of relative benefit. Most trials are neither designed nor sufficiently powered to assess whether the magnitude of cardiovascular risk reduction varies by treatment duration. Consequently, it remains unclear whether BP-lowering therapy follows the compounding efficacy profile observed in cholesterol management or whether treatment effects simply plateau after an initial period.

Utilizing an individual participant-level data (IPD) meta-analysis of randomized controlled trials, we investigated whether antihypertensive therapy confers a cumulative temporal benefit analogous to lipid-lowering interventions, aiming to optimize BP management strategies across the lifecourse.

## Results

### Summary of included studies

Of the 52 randomized trials included in the Blood Pressure Lowering Treatment Trialists’ Collaboration (BPLTTC) resource, one trial was excluded owing to a lack of time-to-event data^16^, leaving 51 trials with a total of 358,642 participants. A total of 1,328 participants were excluded because follow-up time for the primary outcome was missing, leaving 357,314 participants in the analytic cohort. The median follow-up duration across these trials was 4.2 years (interquartile interval: 3.0−5.0 years), ranging from 1.6 years in the Comparison of Amlodipine vs Enalapril to Limit Occurrences of Thrombosis (CAMELOT) trial^17^ to 7.9 years in the United Kingdom Prospective Diabetes Study (UKPDS)^18^. The number of participants contributing data to the primary outcome declined progressively across annual follow-up intervals (0−1, 1−2, 2−3, 3−4, 4−5 and >5 years), due to censoring and event occurrence, from 357,314 participants in the first year to 82,678 participants at more than 5 years (Table 1).

**Table 1:** Baseline characteristics of participants contributing to each follow-up interval for the primary outcome, by treatment arms

Baseline characteristics	Follow-up time intervals (years)											
	0−1 years		1−2 years		2−3 years		3−4 years		4−5 years		>5 years	
	Treatment	Control	Treatment	Control	Treatment	Control	Treatment	Control	Treatment	Control	Treatment	Control
Number of participants	168,980	188,334	158,669	176,296	146,108	162,675	120,181	134,429	86,599	98,349	39,286	43,392
Age (years)	64.8 (9.8)	65.2 (9.6)	64.7 (9.8)	65.1 (9.5)	64.5 (9.7)	64.9 (9.5)	64.2 (9.7)	64.6 (9.5)	63.4 (9.7)	64.0 (9.5)	61.7 (10.4)	62.5 (10.3)
Sex (women)	70,495 (41.7%)	78,044 (41.4%)	66,280 (41.8%)	73,057 (41.4%)	61,130 (41.8%)	67,507 (41.5%)	49,328 (41.0%)	54,639 (40.6%)	34,545 (39.9%)	38,749 (39.4%)	16,399 (41.7%)	18,033 (41.6%)
Systolic BP (mmHg)	152.6 (21.2)	152.2 (21.3)	152.4 (21.1)	152.0 (21.3)	152.3 (21.1)	151.8 (21.3)	152.6 (21.4)	152.0 (21.6)	152.5 (21.8)	151.8 (21.9)	155.8 (21.7)	155.2 (22.0)
Diastolic BP (mmHg)	87.5 (12.4)	87.2 (12.4)	87.6 (12.4)	87.2 (12.4)	87.7 (12.4)	87.3 (12.4)	87.9 (12.6)	87.5 (12.5)	88.4 (12.7)	87.8 (12.6)	91.4 (12.7)	90.7 (12.8)
Body mass index (kg m^−^^2^)	28.0 (5.1)	28.1 (5.1)	28.0 (5.1)	28.1 (5.1)	28.1 (5.1)	28.2 (5.1)	28.1 (5.0)	28.2 (5.0)	28.3 (5.0)	28.4 (5.0)	28.5 (5.1)	28.6 (5.1)
Comorbidity												
Cardiovascular disease	74,841 (44.6%)	83,559 (44.5%)	70,214 (44.5%)	78,314 (44.5%)	63,613 (43.7%)	71,386 (43.9%)	48,111 (40.2%)	55,372 (41.2%)	33,982 (39.4%)	40,341 (41.1%)	10,425 (26.6%)	11,919 (27.5%)
Ischemic heart disease	54,202 (35.5%)	60,218 (37.4%)	51,160 (35.7%)	56,847 (37.7%)	47,696 (36.1%)	53,276 (38.4%)	37,199 (34.8%)	42,642 (37.5%)	27,389 (36.3%)	32,315 (39.5%)	8,231 (24.9%)	9,363 (27.2%)
Atrial fibrillation	5,049 (6.5%)	5,427 (6.2%)	4,611 (6.4%)	4,950 (6.0%)	4,200 (6.4%)	4,537 (6.0%)	3,814 (6.6%)	4,110 (6.2%)	2,904 (6.4%)	3,131 (5.9%)	1,034 (4.9%)	1,079 (4.6%)
Cerebrovascular disease	24,447 (17.5%)	26,233 (17.7%)	22,534 (17.1%)	24,129 (17.3%)	18,894 (15.6%)	20,359 (15.9%)	12,675 (13.0%)	14,084 (13.5%)	7,463 (10.8%)	8,798 (11.6%)	2,094 (6.6%)	2,332 (7.0%)
Chronic kidney disease	11,225 (15.5%)	11,081 (15.7%)	10,661 (15.6%)	10,474 (15.7%)	10,187 (16.0%)	9,966 (15.9%)	9,157 (18.9%)	8,906 (18.9%)	6,475 (22.2%)	6,329 (22.6%)	5,017 (33.1%)	4,952 (33.7%)
Diabetes	48,201 (28.5%)	54,509 (29.0%)	44,789 (28.2%)	50,583 (28.7%)	40,902 (28.0%)	46,442 (28.6%)	34,168 (28.4%)	38,817 (28.9%)	25,493 (29.5%)	29,274 (29.8%)	9,973 (25.4%)	11,315 (26.1%)
Previous use of non-trial medications												
ACE inhibitors	26,662 (32.3%)	31,552 (35.5%)	24,989 (32.3%)	29,536 (35.5%)	22,563 (32.3%)	26,833 (35.5%)	16,844 (31.0%)	20,929 (34.7%)	12,526 (32.9%)	16,406 (37.2%)	3,452 (39.4%)	4,218 (41.4%)
ARB	4,290 (8.4%)	4,252 (8.7%)	3,985 (8.4%)	3,934 (8.6%)	3,589 (8.5%)	3,514 (8.7%)	2,994 (8.8%)	2,994 (9.1%)	1,394 (6.2%)	1,424 (6.5%)	376 (7.3%)	402 (7.9%)
CCB	31,094 (32.8%)	32,816 (32.4%)	29,204 (32.7%)	30,716 (32.3%)	26,721 (33.0%)	28,117 (32.4%)	21,663 (33.4%)	23,118 (32.7%)	15,385 (33.1%)	17,051 (32.5%)	3,674 (30.2%)	4,089 (30.2%)
Diuretic	19,256 (21.3%)	21,711 (22.5%)	17,945 (21.2%)	20,107 (22.2%)	16,230 (21.2%)	18,201 (22.0%)	12,610 (20.8%)	14,421 (21.6%)	9,243 (21.7%)	10,905 (22.5%)	2,463 (23.5%)	2,937 (24.8%)
β-blocker	27,800 (29.4%)	32,074 (31.7%)	26,167 (29.4%)	30,287 (31.9%)	24,112 (29.8%)	28,182 (32.5%)	21,632 (33.4%)	25,606 (36.2%)	17,821 (38.4%)	21,441 (40.9%)	4,597 (37.8%)	5,401 (39.9%)
α-blocker	3,108 (4.7%)	3,430 (4.7%)	2,918 (4.7%)	3,196 (4.6%)	2,681 (4.8%)	2,956 (4.7%)	1,997 (4.8%)	2,254 (4.7%)	1,401 (5.0%)	1,593 (4.6%)	427 (4.5%)	474 (4.3%)
Anticoagulant medications	3,013 (7.8%)	3,554 (7.7%)	2,834 (7.8%)	3,328 (7.7%)	2,647 (7.8%)	3,093 (7.6%)	2,482 (7.8%)	2,886 (7.5%)	1,997 (7.9%)	2,342 (7.4%)	668 (8.9%)	790 (8.8%)
Antiplatelet medications	22,024 (40.1%)	28,903 (46.2%)	20,841 (40.4%)	27,408 (46.7%)	19,651 (41.0%)	25,909 (47.4%)	18,237 (41.7%)	24,198 (48.2%)	15,056 (43.8%)	20,638 (50.5%)	3,874 (37.7%)	5,058 (43.0%)
Lipid-lowering medications	24,625 (34.3%)	29,734 (39.4%)	23,597 (34.9%)	28,459 (40.0%)	22,536 (35.7%)	27,163 (40.9%)	21,267 (36.4%)	25,710 (41.8%)	17,111 (37.7%)	21,307 (43.8%)	5,419 (30.3%)	6,313 (34.2%)

Values are mean (s.d.) or *n* (%). All characteristics were measured at trial baseline and are shown among participants who remained under follow-up and contributed data to each annual interval. Percentages were calculated using variable-specific non-missing denominators.

### Baseline characteristics

Baseline characteristics at randomization are presented for the subsets of participants contributing data to each follow-up interval. Within each interval, treatment and control groups remained well balanced. However, participants contributing data to later follow-up intervals were, on average, younger at baseline and had higher baseline BP levels and fewer cardiovascular comorbidities. In the treatment group, the mean age at randomization was 64.8 years in the years 0−1 dataset and 61.7 years in the >5-year group. Mean systolic BP was 152.6 mmHg in the years 0−1 cohort and 155.8 mmHg in the >5-year group, and mean diastolic BP was 87.5 mmHg and 91.4 mmHg, respectively. The proportion of treatment participants with a history of cardiovascular disease was 44.6% in years 0−1 and 26.6% at more than 5 years. By contrast, the prevalence of chronic kidney disease and the history of angiotensin-converting enzyme (ACE) inhibitor and β-blocker use were higher in the groups with longer follow-up durations. Detailed baseline characteristics for each interval are provided in Table 1.

### Effects of BP-lowering treatment on MACE and death across successive follow-up intervals

**Fig. 1:**
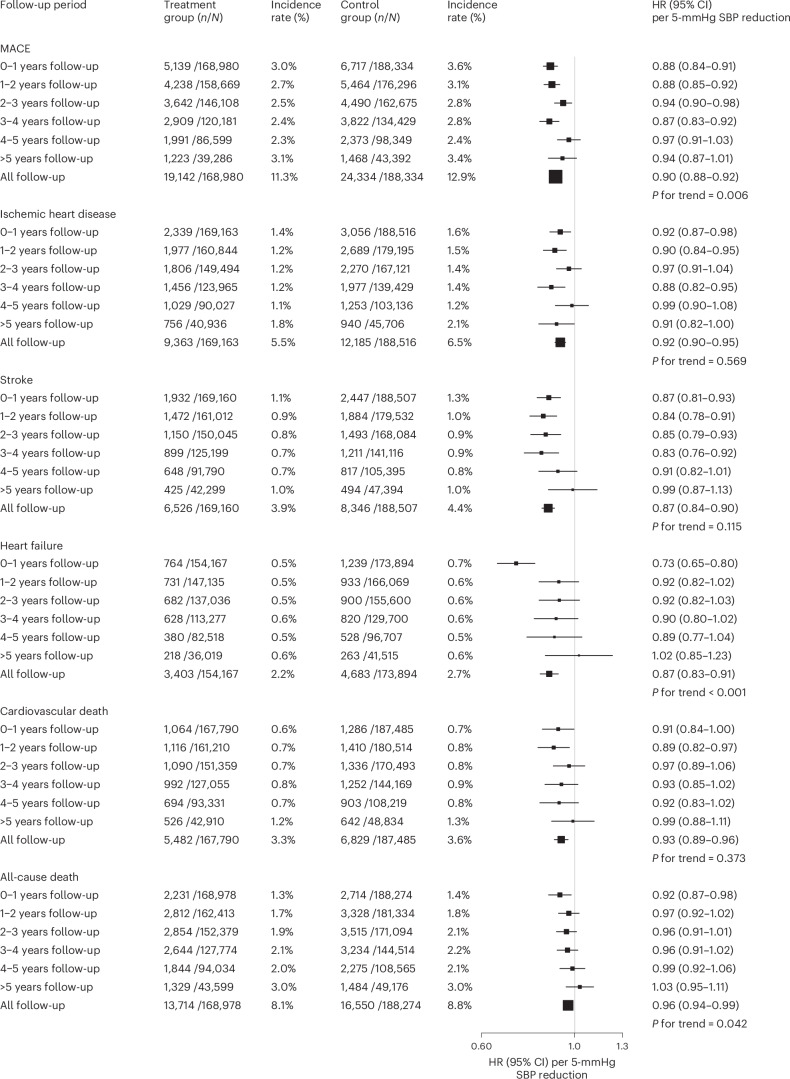
Standardized effects of BP-lowering treatment on primary and secondary outcomes across follow-up intervals. HRs and 95% CIs for MACE, ischemic heart disease, stroke, heart failure, cardiovascular death and all-cause death are shown across successive follow-up intervals (0−1, 1−2, 2−3, 3−4, 4−5 and >5 years), estimated using stratified Cox proportional hazards models. Effect estimates were standardized to a 5-mmHg reduction in systolic BP. Participants contributed data to each interval until the occurrence of the outcome, death or censoring. Squares indicate HR point estimates, with square size proportional to the number of events. *P* values for trend were calculated using two-sided Wald tests to assess linear changes in treatment log-HR across ordered follow-up intervals; for heart failure, *P* = 0.0004. No adjustment was made for multiple comparisons. SBP, systolic blood pressure.

Using prespecified 1-year follow-up intervals, we estimated interval-specific HRs with trial-stratified Cox models, standardized treatment effects to a 5-mmHg reduction in systolic BP and formally tested for linear trend across follow-up periods. The annual incidence of MACE was highest during the first year of follow-up (3.0% in the treatment group versus 3.6% in controls) and then declined during years 1−5, followed by a subsequent rise at more than 5 years (3.1% versus 3.4%). The composition of MACE varied over time: the incidence of stroke and heart failure remained largely constant, whereas ischemic heart disease and cardiovascular death were more frequent in later follow-up periods (Fig. 1). In the primary standardised analysis, there was no evidence of compounding treatment effects with longer follow-up. Relative risk reductions were established early after treatment initiation and then modestly attenuated over time (*P* for trend = 0.006). Specifically, a pharmacological reduction of 5-mm Hg in systolic BP reduced MACE by 12% in the first year (HR 0.88, 95% CI 0.84−0.91), with evidence of effect attenuation in subsequent years: HR 0.88 (0.85−0.92) in year 1−2, HR 0.94 (0.90−0.98) in year 2−3, HR 0.87 (0.83−0.92) in year 3−4, HR 0.97 (0.91−1.03) in year 4−5, and HR 0.94 (0.87−1.01) at >5 years.

Secondary outcomes showed similar patterns of relative risk reduction, without evidence of compounding effects, although statistical precision was reduced (Fig. 1). The most pronounced attenuation was observed for heart failure, where the relative effects were largest in the first year (27% reduction, HR = 0.73, 0.65−0.80), decreased significantly over time (*P* for trend < 0.001) and were compatible with no effect at more than 5 years (1.02 (0.85−1.23)). All-cause death showed suggestive evidence of diminishing effect (*P* for trend = 0.042). For stroke, relative effects appeared smaller in later years (HR = 0.99, 0.87−1.13 at >5 years), but there was no statistically significant trend (*P* for trend = 0.115). Relative effects for ischemic heart disease and cardiovascular death were broadly consistent across follow-up intervals (*P* for trend = 0.569 and 0.373, respectively).

### Continuous effects of BP-lowering treatment on major cardiovascular disease and death over time

**Fig. 2:**
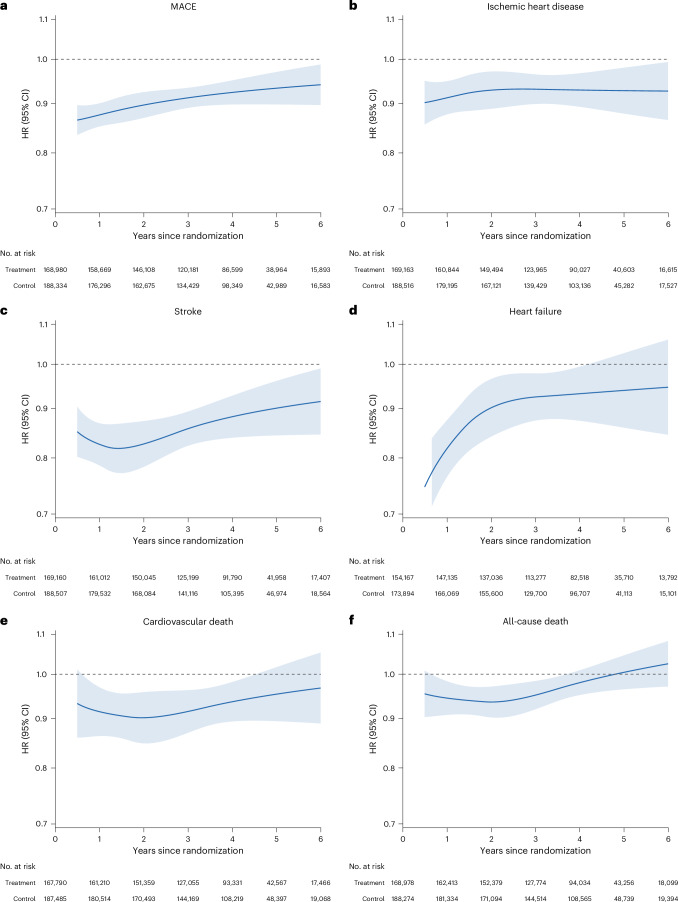
Continuous modeling of BP-lowering treatment effects on primary and secondary outcomes over follow-up time. Estimated HRs and 95% CIs are shown for MACE (**a**), ischemic heart disease (**b**), stroke (**c**), heart failure (**d**), cardiovascular death (**e**) and all-cause death (**f**), standardized to a 5-mmHg reduction in systolic BP, plotted as continuous functions of time since randomization. Solid lines indicate HR estimates; shaded areas represent 95% CIs. The horizontal dashed line denotes no effect (HR = 1). Numbers at risk by treatment group are shown below. For heart failure, the narrow CIs observed early after randomization reflect the mathematical constraints of the Royston−Parmar flexible parametric survival model near the baseline.

To complement the interval-specific analyses, we used flexible parametric survival models to visualize treatment effects continuously over follow-up (Fig. 2). For MACE, significant treatment effects emerged rapidly after randomization and were established early after initiation, with relative risk reductions persisting throughout follow-up without evidence of progressive amplification over time. Secondary outcomes showed a similar rapid onset of treatment effects, although temporal patterns varied across outcomes, and estimates became less precise at longer follow-up. For ischemic heart disease, relative risk reductions were apparent early and appeared broadly stable over time. By contrast, heart failure exhibited a pronounced early treatment effect that attenuated over follow-up and then plateaued. For stroke, early treatment effects were observed, followed by gradual attenuation with increasing uncertainty at later timepoints. For cardiovascular death and all-cause death, early relative risk reductions were evident, but estimates became imprecise and approached the null with longer follow-up (Fig. 2).

### Sensitivity analyses

Sensitivity analyses were performed to assess the robustness of the primary findings to alternative modeling of follow-up time, informative censoring, treatment adherence, trial selection and BP standardization, yielding results consistent with the primary analysis.

First, to assess whether the results were sensitive to the discretization of follow-up time into ordered 1-year intervals, we fitted a continuous-time Cox model using a counting process framework. The results were consistent with those of the primary analysis. There was no evidence of time-dependent modification of treatment effects for MACE (interaction HR = 1.014 per unit increase in log-year, 95% CI: 0.998−1.030; *P* = 0.095) or for secondary outcomes including ischemic heart disease (*P* = 0.704), stroke (*P* = 0.524), cardiovascular death (*P* = 0.992) and all-cause death (*P* = 0.236). The only exception was heart failure, for which treatment effects attenuated significantly over time (interaction HR = 1.108, 95% CI: 1.064−1.153; *P* < 0.001) (Extended Data Table 1).

**Extended Data Fig. 1:**
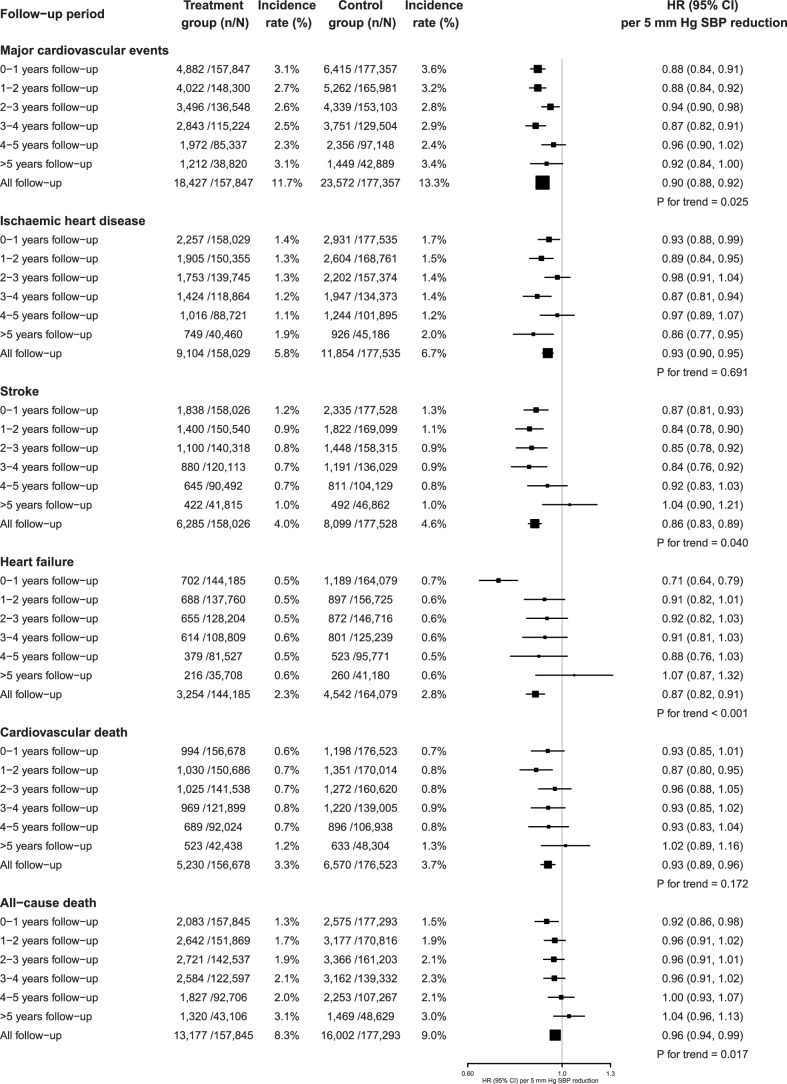
Standardised effects of systolic blood pressure-lowering treatment on outcomes, stratified by follow-up duration, adjusted for selection bias using inverse probability of censoring weighting. Squares indicate hazard ratio (HR) point estimates and horizontal lines indicate 95% confidence intervals. The vertical line indicates HR = 1. n/N indicates the number of participants with events divided by the number of participants included in the corresponding analysis interval. For each follow-up interval, inverse probability of censoring weights (IPCW) were estimated from logistic models for the probability of remaining event-free beyond the corresponding landmark. Stabilised weights used a numerator model including randomised treatment allocation only and a denominator model additionally adjusted for baseline covariates. Weights were estimated among participants surviving beyond each landmark and truncated at the 1st and 99th percentiles. Interval-specific HRs were estimated using trial-stratified weighted Cox models with robust variance estimation and standardised to a 5 mm Hg reduction in systolic blood pressure. *P* values for trend were calculated using two-sided Wald tests from weighted trial-stratified Cox proportional hazards models with robust variance estimation to assess linear changes in the treatment log-hazard ratio across ordered follow-up intervals.

**Extended Data Fig. 2:**
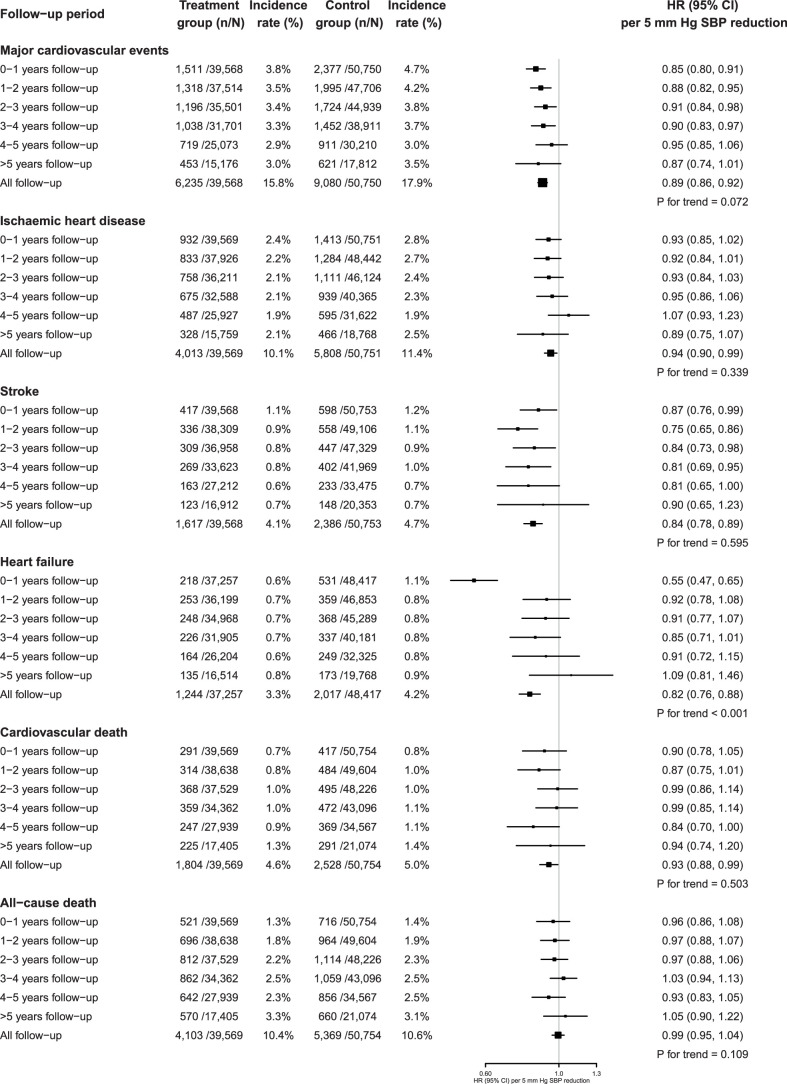
Standardised effects of systolic blood pressure-lowering treatment on outcomes, stratified by follow-up duration, using inverse probability of censoring weighting, incorporating time-varying adherence. Nine trials (AASK, ACCORD, ALLHAT, ASCOT, HIJ-CREATE, IDNT, PROGRESS, SHEP and SPRINT) with longitudinal adherence data were included. Squares indicate hazard ratio (HR) point estimates and horizontal lines indicate 95% confidence intervals. The vertical line indicates HR = 1. n/N indicates the number of participants with events divided by the number of participants included in the corresponding analysis interval. Treatment effects were estimated using trial-stratified weighted Cox models, standardised to a 5 mm Hg reduction in systolic blood pressure. Stabilised inverse probability of censoring weights incorporated baseline covariates and the most recently observed adherence status (high adherence was defined as taking ≥80% of prescribed medication) at each landmark. *P* values for trend were calculated using two-sided Wald tests from weighted trial-stratified Cox proportional hazards models with robust variance estimation to assess linear changes in the treatment log-hazard ratio across ordered follow-up intervals; for heart failure, *P* = 1.18 × 10^−5^. No adjustment was made for multiple comparisons.

Second, to help address potential selection bias arising from informative censoring across follow-up intervals, we applied inverse probability of censoring weighting (IPCW) based on measured baseline covariates. After weighting, key covariates were well balanced across follow-up cohorts compared to the unweighted analyses (Extended Data Table 2). IPCW-adjusted estimates aligned with the primary analysis and again showed no evidence of compounding treatment effects (Extended Data Fig. 1). For MACE, the HR per 5-mmHg systolic BP reduction was 0.88 (0.84−0.91) in years 0−1 and tended to diminish but remained effective after 5 years with HR = 0.91 (0.84−1.00) (*P* for trend = 0.022). Attenuation remained most evident for heart failure, whereas ischemic heart disease and cardiovascular death showed no clear time-varying effects. To further account for time-varying factors related to treatment persistence and evolving clinical status, we extended the IPCW model in the subset of nine trials with repeated adherence assessments by incorporating time-updated adherence. Results from this expanded model were again consistent with the primary findings (Extended Data Fig. 2).

**Extended Data Fig. 3:**
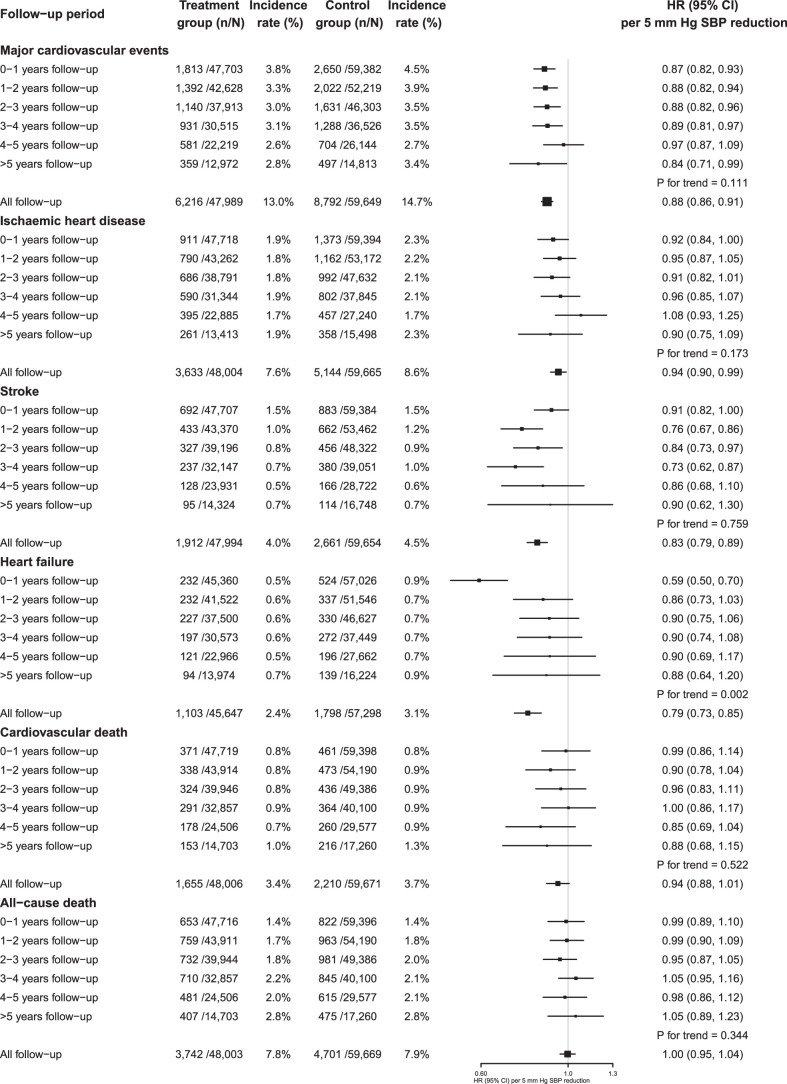
Standardised effects of systolic blood pressure-lowering treatment on outcomes among participants with high adherence, stratified by follow-up duration. Twelve trials collected and shared adherence data have been included in this analysis. For nine trials (AASK, ACCORD, ALLHAT, ASCOT, HIJ-CREATE, IDNT, PROGRESS, SHEP, and SPRINT) with longitudinal adherence data, a counting process model was employed. Only follow-up periods where participants maintained high adherence status (defined as taking ≥80% of prescribed medication) were included in the analysis. For three trials (ANBP, PREVENT, and PROFESS) lacking longitudinal data, the entire follow-up period of participants who demonstrated overall high adherence (taking ≥80% of prescribed medication) was included. Squares indicate hazard ratio (HR) point estimates and horizontal lines indicate 95% confidence intervals. The vertical line indicates HR = 1. n/N indicates the number of participants with events divided by the number of participants included in the corresponding analysis interval. Treatment effects were estimated using trial-stratified Cox models and standardised to a 5 mm Hg reduction in systolic blood pressure. *P* values for trend were calculated using two-sided Wald tests of the treatment-by-time interaction term.

Third, to examine the potential influence of declining adherence over time, we restricted the analyses to participants with high adherence (defined as taking ≥80% of assigned medication) in the 12 trials with available overall adherence data. In this high-adherence subgroup, there was no evidence of an increase in treatment benefit with longer follow-up for MACE (*P* for trend = 0.111). Notably, the attenuation observed in the primary analysis was not evident: the HR at more than 5 years was 0.84 (95% CI: 0.71−0.99), similar to the first-year effect (HR = 0.87, 95% CI: 0.82−0.93). Temporal patterns for secondary outcomes were broadly similar to the primary analysis, with heart failure again showing the largest early benefit, followed by attenuation (*P* for trend = 0.002) (Extended Data Fig. 3).

Finally, results were also consistent after excluding trials terminated early for benefit (Supplementary Fig. 1), excluding trials comparing different antihypertensive drug classes (Supplementary Fig. 2), analyzing treatment effects without BP reduction standardization (Supplementary Fig. 3), standardization using within-interval BP differences (Supplementary Fig. 4) and standardization to a 3-mmHg reduction in diastolic BP (Supplementary Fig. 5). Across these analyses, there was no evidence of compounding treatment effects over time across outcomes.

### Subgroup analyses

**Fig. 3:**
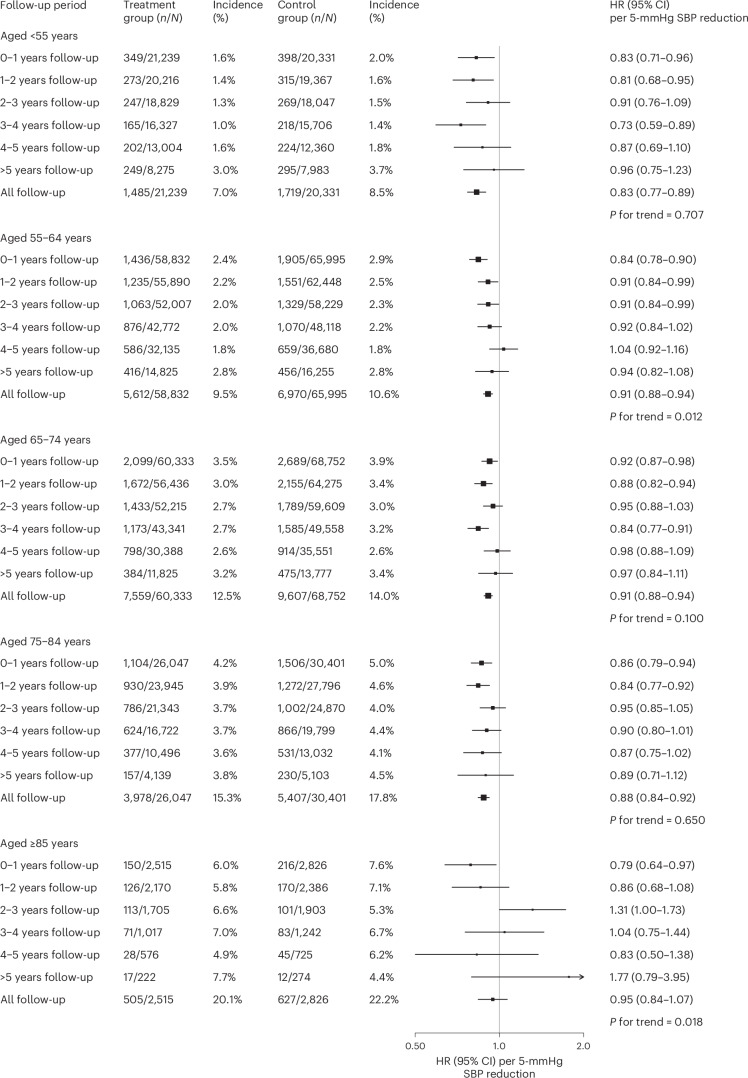
Effects of BP-lowering treatment on MACE, stratified by follow-up duration and baseline age groups. HRs and 95% CIs for MACE are shown across successive follow-up intervals (0−1, 1−2, 2−3, 3−4, 4−5 and >5 years) and overall within baseline age groups <55, 55−64, 65−74, 75−84 and ≥85 years. Estimates were derived from trial-stratified Cox proportional hazards models and standardized to a 5-mmHg reduction in SBP. Squares indicate HR point estimates; horizontal lines indicate 95% CIs. *P* values for trend were derived within each age group using two-sided Wald tests to assess linear changes in the treatment log-HR across ordered follow-up intervals. SBP, systolic blood pressure.

Subgroup analyses by age category (<55, 55−64, 65−74, 75−84 and ≥85 years) showed broadly similar patterns across all groups. Relative treatment effects for MACE remained stable or tended to decline across follow-up intervals, with no evidence of compounding effects (Fig. 3). Broadly similar patterns were observed for secondary outcomes (Supplementary Figs. 6−10) as well as in analyses stratified by sex (Supplementary Figs. 11 and 12) and baseline diabetes status (Supplementary Figs. 13 and 14).

### Effects of major antihypertensive drug classes on major cardiovascular disease and death across successive follow-up intervals

**Fig. 4:**
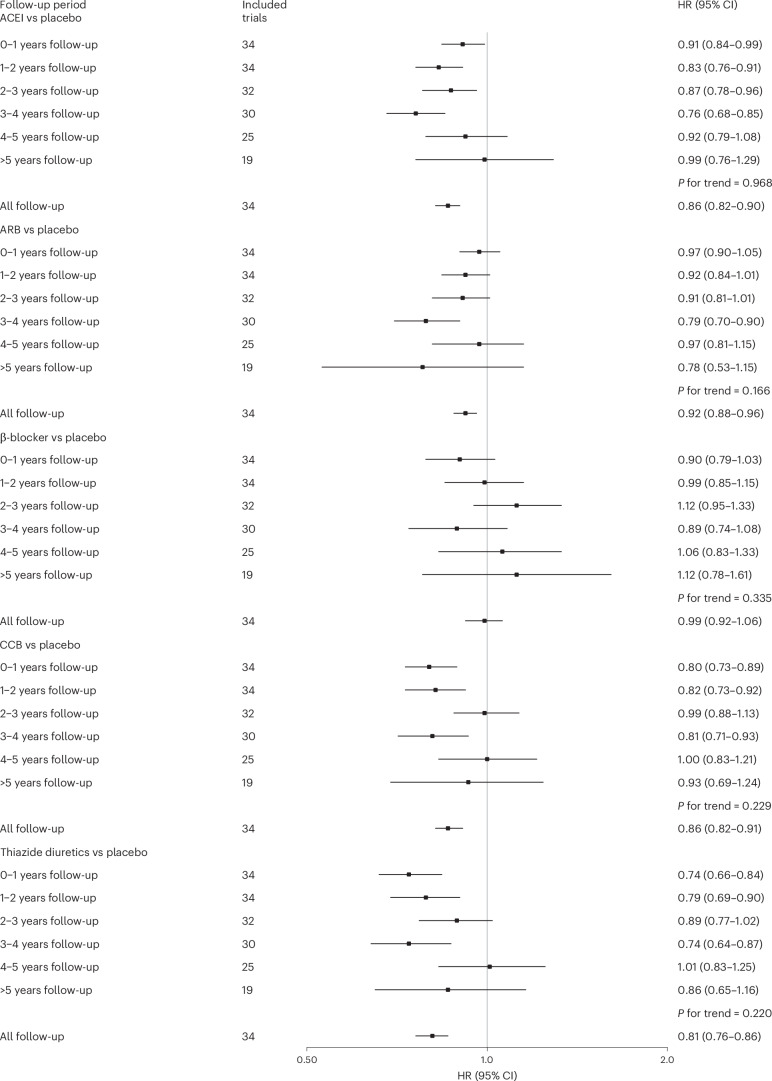
Effects of antihypertensive drug classes on MACE, stratified by follow-up duration. HRs and 95% CIs for MACE are shown for the major antihypertensive drug classes relative to placebo across successive follow-up intervals (0−1, 1−2, 2−3, 3−4, 4−5 and >5 years). Estimates were derived from network meta-analyses combining two-arm and multi-arm randomized trials, using log-HRs and corresponding standard errors from trial-level Cox models. Squares represent point estimates; horizontal lines indicate 95% CIs. *P* values for trend were calculated using meta-regression of interval-specific log-HRs to assess evidence for a linear change in treatment effects across ordered follow-up intervals within each drug class, with two-sided Knapp−Hartung tests for linear trend across ordered follow-up intervals. ACEI, ACE inhibitor.

To examine whether the temporal pattern of treatment effects differed by antihypertensive drug class, we conducted an IPD network meta-analysis across the drug classes included in the eligible trials. The network meta-analysis included 34 trials comparing five major antihypertensive drug classes. Across all drug classes, there was no evidence of a compounding benefit for MACE across follow-up intervals (all *P* for trend > 0.05; Fig. 4), and a similar pattern was observed for most individual cardiovascular outcomes and all-cause death (Supplementary Figs. 15−19). However, angiotensin II receptor blockers (ARBs) demonstrated a non-significant effect in preventing stroke during the first year (HR = 0.92, 95% CI: 0.84−1.02), with a greater reduction emerging over time (*P* for trend = 0.046; Supplementary Fig. 16). By contrast, the early protective effects of thiazide diuretics on heart failure peaked in the first year (HR = 0.40, 95% CI: 0.30−0.54) and declined soon thereafter (*P* for trend = 0.034; Supplementary Fig. 17), which may explain the attenuation of heart failure risk reduction observed in the primary analysis. Additionally, diminishing effects were observed for heart failure with calcium channel blockers (CCBs) (*P* for trend = 0.010) and for all-cause death with thiazide diuretics (*P* for trend = 0.012).

### Cumulative absolute risk reduction analyses

To complement the primary analyses on the relative scale, we estimated fixed-horizon cumulative absolute risk reductions from randomization. In these analyses, which were anchored to the original randomized population, cumulative absolute risk reductions standardized to a 5-mmHg lower systolic BP increased over time for MACE and several secondary outcomes (Extended Data Table 3). For MACE, the absolute risk reduction increased from 0.30% (95% CI: 0.20−0.40) at 1 year to 1.25% (0.94−1.57) at 5 years, corresponding to numbers needed to treat of 337 and 80, respectively. For stroke, the corresponding estimates were 0.07% (0.02−0.12) at 1 year and 0.52% (0.36−0.68) at 5 years, corresponding to numbers needed to treat of 1,535 and 192, respectively. Absolute benefits also accumulated for death outcomes: for cardiovascular death, from 0.04% (−0.00 to 0.08) at 1 year to 0.29% (0.12−0.47) at 5 years; and for all-cause death, from 0.10% (0.04−0.17) to 0.36% (0.11−0.62). For ischemic heart disease, the benefit became more apparent after 1 year, whereas estimates for heart failure were smaller and less consistent. These findings indicate that cumulative absolute benefit accrues over time even though interval-specific proportional effects do not progressively amplify.

## Discussion

This IPD meta-analysis of 51 randomized trials provides robust evidence of rapid-onset cardiovascular benefits after initiation of BP-lowering treatment, with benefits sustained but without evidence of compounding. Across most outcomes, relative risk reductions were largely established soon after treatment initiation and then remained stable or modestly attenuated over time, with later interval-specific estimates conditional on the evolving risk set. For heart failure prevention, relative risk reductions were most pronounced in the first year and decreased thereafter, a pattern consistent with the rapid hemodynamic effects of antihypertensive therapy. Our network meta-analysis further confirmed that this pattern was broadly consistent across the major antihypertensive drug classes, including ACE inhibitors, ARBs, β-blockers, CCBs and thiazide diuretics. Complementary fixed-horizon analyses on the absolute scale showed that cumulative absolute benefit continued to accrue with longer treatment exposure.

Studies of BP lowering typically report average treatment effects across the entire follow-up period. Owing to limited sample sizes, few studies have evaluated how relative risk reductions may vary with treatment duration. Thus, it has remained uncertain whether cardiovascular benefits from BP-lowering therapy continue to accrue or plateau after initial gains. Mendelian randomization studies, often interpreted as proxies for lifelong BP reduction, tend to show lower HRs than short-term trials^8,9,15^, fueling the hypothesis of compounding benefits over time and motivating recent calls for early cardiovascular prevention strategies^19^; however, differences in study design and assumptions limit direct comparability^20^. Analysis of long-term treatment effects from individual clinical trials after the end of randomized therapy can provide another perspective on potential compounding effects. So far, some studies have provided supporting evidence for the existence of ‘legacy effects’^7,21^, implying that longer duration of antihypertensive therapy may lead to cumulative vascular protection that persists even after treatment cessation. For example, in the Anglo-Scandinavian Cardiac Outcomes Trial (ASCOT) Legacy Study, participants originally assigned to the amlodipine-based regimen had lower long-term cardiovascular death^21^. However, these findings have not been confirmed in long-term follow-up studies of large trials, such as the Systolic Blood Pressure Intervention Trial (SPRINT) and the Antihypertensive and Lipid-Lowering Treatment to Prevent Heart Attack Trial (ALLHAT)^22,23^.

By leveraging the largest IPD resource available to date, our study directly addresses this evidence gap by estimating treatment effects across successive annual follow-up intervals. Notably, each interval included large numbers of participants, ranging from over 350,000 in the first year to over 80,000 at more than 5 years. Across these intervals, we observed no evidence of compounding relative benefits over time for MACE or all-cause death. Treatment benefits were evident within the first months of follow-up and did not increase with longer treatment duration; instead, they tended to plateau or attenuate modestly over time. When treatment effects were modeled as continuous functions of follow-up time, estimates derived at finer temporal resolution demonstrated patterns consistent with the primary time-stratified analyses, providing no evidence of progressive amplification with longer treatment duration.

For individual components of MACE, broadly similar results were observed for ischemic heart disease and cardiovascular death, whereas variations have been found in stroke and heart failure. Regarding stroke prevention, ARBs demonstrated a gradual increase in relative risk reduction over time, consistent with previous observations of delayed stroke benefit in trials such as the PRoFESS trial^24,25^. By contrast, for heart failure, we observed a pronounced initial reduction in incidence during the first year, which diminished significantly in subsequent years. Our network meta-analysis indicates that this pattern is largely driven by diuretics, which exhibit a similar early benefit in year 1 that declines soon afterwards. This may be attributed to the fact that diuretics rapidly relieve heart failure symptoms associated with fluid overload, thereby preventing the clinical manifestation of heart failure events^26^. A directionally similar temporal pattern was also observed for CCBs; however, none of the estimates was statistically significant and should be interpreted cautiously.

Notably, the absence of progressively increasing proportional effects should not be interpreted as evidence of no cumulative absolute treatment benefit. In complementary fixed-horizon analyses anchored to the original randomized population, cumulative absolute risk reductions increased steadily over time for MACE and several secondary outcomes. These analyses provide an important complement to the interval-specific HRs because they are estimated on the absolute scale and do not condition on event-free survival within successive follow-up intervals. Together, the relative-scale and absolute-scale results suggest that BP-lowering treatment confers protection early and that continued exposure allows this benefit to accumulate over time in absolute terms but without progressive amplification of proportional effects.

These findings have potentially major implications for cardiovascular prevention and clinical decision-making. Our results do not support the assumption that the relative effects of BP lowering necessarily amplify with longer treatment duration during the period of available randomized follow-up. Rather, the rationale for early and sustained treatment lies in the early establishment of relative benefit and the continued accrual of absolute risk reduction with ongoing exposure. As a result, treatment decisions should remain guided primarily by baseline cardiovascular risk and expected absolute benefit^13^ rather than by an expectation of progressively larger proportional effects over time. Combined with previous BPLTTC findings demonstrating consistent relative risk reductions across age groups^2^, and age-stratified analyses in this study, our findings do not support changing treatment thresholds solely on the assumption that earlier exposure yields progressively larger proportional benefits.

The non-compounding benefit observed with BP-lowering treatment contrasts markedly with that of cholesterol-lowering therapies. Meta-analyses from the Cholesterol Treatment Trialists’ (CTT) Collaboration found that reducing low-density lipoprotein cholesterol (LDL-C) lowers cardiovascular risk by about 14% in the first year but by 20−30% in each subsequent year up to more than 5 years (*P* for interaction < 0.001)^12^. Similarly, Wang et al.^11^ demonstrated a compounding effect of LDL-C lowering, with meta-regression analyses showing that cholesterol lowering leads to progressively larger reductions in cardiovascular risk with longer follow-up. Although similar compounding effects might have been anticipated for antihypertensive treatment, our results do not support this expectation. These contrasting efficacy profiles may reflect differences in underlying mechanisms: continued exposure to LDL-C leads to the development of atherosclerotic plaque^27,28^, and cholesterol-lowering therapies (such as statins) act on atherosclerotic plaques, promoting gradual stabilization and regression and resulting in accumulating benefit over time^29,30^, whereas antihypertensive therapies primarily alleviate mechanical arterial stress, which may explain their rapid and stable risk reductions^31^. Although BP reduction may also favorably modify arterial structure^32^, it may not contribute to a compounding effect.

Key strengths of our study include access to the largest IPD resource from randomized trials to date, facilitating robust estimates for each year of treatment duration. Notably, consistent findings across multiple complementary analytical approaches strengthened confidence in the robustness of the results. Additionally, the inclusion of multiple drug classes and comparison types (placebo and active-controlled trials) enabled, to our knowledge, the first comprehensive network meta-analyses investigating drug-class-specific effects over time.

This study also has some limitations. First, trials included in this study had a median follow-up of only 4.2 years, which is potentially insufficient to detect longer-term benefits. Considering that BP-lowering therapy may prevent or delay hypertension-induced pathological changes such as atherosclerosis^33^, left ventricular hypertrophy^34^ and carotid intima-media thickening^35^, longer-term evidence is needed to fully elucidate any potential compounding effects. Because trials typically end early once benefit is proven, post-trial extensions are necessary to assess long-term outcomes. Second, the interpretation of the time-stratified estimates is limited because participants contributing to later intervals had survived and remained event free and uncensored in earlier intervals. Interval-specific HRs, therefore, reflect evolving risk sets rather than a fixed baseline population, and IPCW cannot fully account for unmeasured factors related to censoring. However, the overall pattern was similar across sensitivity analyses and was not materially altered by alternative modeling of follow-up time, changes in cohort composition, attenuation of BP separation or declining adherence. These findings make it less likely that the absence of progressively stronger effects was explained solely by informative censoring. Third, the present analysis did not evaluate the effects of treatment on other outcomes, such as cognitive function and chronic kidney disease, over time, and further studies will be essential to clarify these patterns. Fourth, because the mean age of the trial population was over 60 years, and even the youngest subgroup (<55 years) represented midlife rather than early adulthood, these findings should not be extrapolated directly to treatment initiated in early adulthood. However, to our knowledge, trials conducted in low-risk younger individuals are lacking.

In conclusion, this study challenges the widely held assumption that the benefits from antihypertensive treatment compound over a longer duration of therapy. Instead, our findings show that antihypertensive therapy provides substantial cardiovascular benefits early, which are maintained over time without progressive amplification, although later interval estimates should be interpreted in the context of changing risk sets over time. Sustaining these benefits likely requires continued adherence over the long term. The early emergence of relative treatment effects suggests that clinically meaningful benefit can be achieved soon after antihypertensive treatment is initiated. On the other hand, the absence of compounding effects suggests that targeting individuals at higher cardiovascular risk may be more efficient than expanding treatment to lower-risk individuals on the assumption that longer treatment duration yields increasingly larger proportional benefits.

## Methods

This IPD meta-analysis follows the current recommendations of the PRISMA-IPD statement^36^. The PRISMA-IPD checklist was completed (Supplementary Table 1).

### Study design

We conducted a time-stratified IPD meta-analysis of eligible BP-lowering trials using the resource provided by the BPLTTC^37,38^. A study protocol was developed before releasing a dataset for statistical analysis and was finalized with extensive feedback from international collaborators and the BPLTTC steering committee.

To date, this resource provides IPD from 52 randomized trials and 363,684 participants^2,3,37^. Participants in each study were randomly assigned to intervention or control groups, and analyses followed the intention-to-treat principle. Characteristics at trial baseline, including age, sex, systolic and diastolic BP, body mass index, history of cardiovascular disease, diabetes, prior use of antihypertensive medication and other relevant clinical variables, were summarized according to different follow-up durations. In addition, we reported the median follow-up time of participants included in the time-stratified analyses for each follow-up interval.

### Eligibility criteria

We used the most current BPLTTC dataset, which comprised data contributed by participating trials, including BP values at baseline and during follow-up, primary and secondary outcomes with event timing and baseline characteristics of interest. Participants with a known diagnosis of heart failure at trial baseline were excluded from this analysis, because established heart failure defines a clinically distinct population, with different treatment goals, risk profiles and outcome definitions from the broader hypertensive population, which is commonly excluded from individual antihypertension trials^2,37^.

### Outcomes

The primary outcome was MACE, defined as a composite of fatal or non-fatal stroke, myocardial infarction or ischemic heart disease or heart failure causing death or requiring admission to hospital. Secondary outcomes were all-cause death and each component of the primary outcome.

### Comparison groups

Individual trials were categorized into two groups: intervention and comparator. For placebo-controlled trials, the placebo group was regarded as the comparator, and the active drug group served as the intervention. In trials with two or more active groups, including those comparing different drug classes, the group with the greatest BP reduction was designated the intervention, and the other treatment group(s) were the comparators. Trials that compared more intense versus less intense treatments were classified into intervention and comparator groups, respectively. Detailed information concerning the comparison groups, trial design, participant characteristics and the extent of BP reduction in each trial was reported previously^2,3,37^.

### Statistical analyses

We analyzed the data on an intention-to-treat basis, according to the groups to which participants were originally randomized (intervention versus comparator). IPD from each trial were harmonized before statistical analysis and merged into a single dataset, enabling a one-stage IPD meta-analysis. Participants contributed data from randomization until the earliest occurrence of the outcome of interest or censoring.

### Primary time-stratified analysis

To evaluate how treatment effects evolved over time, the median intervention duration of 4.2 years (interquartile interval: 3.0−5.0 years) was divided into discrete 1-year intervals: 0−1, 1−2, 2−3, 3−4, 4−5 and >5 years^39,40^. Participants contributed follow-up time to each interval until they developed the outcome, died or were otherwise censored. All participants were included in the 0−1-year interval, but only event-free participants continued to contribute in subsequent intervals. For each interval, follow-up was truncated at the end of the interval, so that only events occurring within that interval were counted in its analyses.

Separate datasets were created for each interval using a consistent censoring and truncation rule. Within each interval’s dataset, a Cox proportional hazards model was employed, stratified by trial, to estimate HRs for primary and secondary outcomes over time. Effect sizes of each interval were standardized to a 5-mmHg reduction in systolic BP using trial-specific mean between-group BP differences estimated from follow-up measurements, excluding the first year^2,3^. These differences were estimated using a linear mixed-effects model and incorporated into the Cox model as a continuous trial-level variable interacting with treatment allocation, allowing treatment effects to be compared on a common, clinically interpretable scale while accounting for between-trial differences in treatment intensity^40^.

### Formal assessment of temporal trend in treatment effects

To formally assess whether treatment effects changed across follow-up periods, follow-up time was modeled using a time-split approach, with successive 1-year intervals represented by a numeric time period index. In the survival analysis, a stratified Cox proportional hazards model including an interaction term between treatment allocation and this time variable was fitted, and the interaction term was assessed using a Wald test to evaluate a linear trend in treatment effects with increasing follow-up duration^38,41^.

### Continuous-time modeling of treatment effects

To complement the primary time-split analyses and provide a smooth graphical description of how treatment effects evolved over time without relying on arbitrary interval cutpoints, we additionally modeled treatment effects using a complementary continuous-time approach based on flexible parametric survival models. Specifically, for each outcome, we fitted Royston−Parmar spline-based survival models with three degrees of freedom for the baseline log cumulative hazard^42^, allowing the treatment effect to vary smoothly over time by including a time-varying coefficient treatment allocation. Models were stratified by trial, and effect estimates were standardized to a 5-mmHg difference in systolic BP. Time-varying HRs were estimated across follow-up by predicting model-based treatment effects at 30-day intervals from 30 days after randomization up to 6 years of follow-up.

### Sensitivity analyses and subgroup assessment

Several sensitivity analyses were conducted to assess the robustness of the primary findings. First, to evaluate whether the results were sensitive to the modeling of follow-up time, we fitted an alternative time-dependent Cox proportional hazards model using a counting process framework, in which follow-up time was treated as a continuous variable and the treatment effect was allowed to vary with time^43^, complementary to the primary time-split analysis. Second, to account for potential selection bias due to informative censoring across follow-up intervals, estimates were adjusted using IPCW^44^. Stabilized weights were derived from logistic regression models estimating the probability of remaining uncensored beyond each follow-up interval, conditioned on randomized treatment allocation, age, sex, baseline systolic BP, body mass index, history of cardiovascular disease, history of diabetes and prior use of antihypertensive drugs. To assess covariate balance, the distributions of these variables were examined before and after weighting across follow-up cohorts. The IPCW-adjusted HRs were estimated using weighted Cox proportional hazards models stratified by trial and were standardized to a 5-mmHg reduction in systolic BP. In addition, to account for potential informative censoring related to time-varying treatment adherence, we repeated the IPCW analyses in the subset of nine trials with repeated adherence assessments. Stabilized weights were estimated using the same baseline covariates as above, plus the most recently observed adherence status at each landmark, and weighted Cox models additionally adjusted for interval-specific adherence status. Third, to examine the potential influence of declining treatment adherence over time, analyses were restricted to participants with high adherence (defined as taking at least 80% of the prescribed medication) across 12 trials with available adherence data. Fourth, trials comparing two antihypertensive drug classes were excluded, as they typically achieve similar BP control across treatment groups. Fifth, analyses were repeated after excluding trials that were terminated early because of clear evidence of benefit in the treatment groups. Finally, additional sensitivity analyses were performed to evaluate the robustness of standardization methods. These included estimating unstandardized treatment effects without accounting for BP reduction, as well as standardizing treatment effects using within-interval BP differences, by calculating the mean BP difference between treatment groups separately for each follow-up interval rather than using a single overall BP difference as in the primary analysis, and standardized to a 3-mmHg reduction in diastolic BP, which was the mean diastolic BP reduction between randomized groups, excluding the first 12 months across all trials^2^.

We examined the time-stratified effects of BP-lowering treatment on cardiovascular disease risk by baseline age groups (<55, 55−64, 65−74, 75−84 and ≥85 years), sex and diabetes status. Potential variations in treatment effects across follow-up periods were then evaluated within each subgroup.

### Network meta-analysis

We used IPD network meta-analysis to compare the effects of five major antihypertensive drug classes across different treatment intervals: ACE inhibitors, ARBs, β-blockers, CCBs and thiazide diuretics. Unlike the primary analyses, this network meta-analysis estimated total class effects, capturing both BP-mediated and non-BP-mediated effects, rather than standardizing effects to a fixed BP reduction magnitude. Because BP-lowering intensity trials did not compare specific antihypertensive drug classes, only placebo-controlled and drug class comparison trials were included in this analysis. The network meta-analysis was conducted at the level of randomized drug class allocation, following the intention-to-treat principle. Estimated effects, therefore, reflected assignment to a given drug class strategy, including any protocol-permitted background or add-on therapy. Trial arms in which the randomized intervention consisted of fixed combination therapies were excluded to ensure that each comparison could be attributed to a single drug class. A Cox proportional hazards model was used to estimate the HRs for each pairwise comparison using IPD from each trial, with censoring applied at the upper boundary of each follow-up interval. To run the network meta-analysis, we used the Markov chain Monte Carlo simulation approach with four chains and 100,000 iterations after an initial burn-in of 10,000 (ref. ^45^). To assess whether treatment effects changed across follow-up, we performed a meta-regression of the interval-specific treatment estimates derived from the IPD network meta-analysis, with the Knapp−Hartung adjustment applied^46^.

### Complementary analysis

As a complementary analysis, we estimated cumulative absolute risk reductions from randomization at 1, 2, 3, 4 and 5 years. Within each trial, fixed-horizon risks were estimated separately by randomized group using Kaplan−Meier methods for all-cause death and cumulative incidence functions for non-fatal outcomes, with death treated as a competing event where applicable. Trial-specific absolute risk reductions were defined as the difference in cumulative risk between control and treatment groups at each horizon and were pooled using two-stage fixed-effect meta-analysis. To standardize estimates across trials, absolute risk reductions were additionally rescaled to a 5-mmHg lower systolic BP on the basis of the achieved trial-level between-group systolic BP difference. Numbers needed to treat were derived as the inverse of the pooled absolute risk reduction when positive. Statistical analyses were performed using R (v.4.2.2).

### Reporting summary

Further information on research design is available in the Nature Portfolio Reporting Summary linked to this article.

## Online content

Any methods, additional references, Nature Portfolio reporting summaries, source data, extended data, supplementary information, acknowledgements, peer review information; details of author contributions and competing interests; and statements of data and code availability are available at 10.1038/s41591-026-04514-3.

## Supplementary information

Supplementary Information Figs. 1−19 and Table 1.

Reporting Summary

Peer Review File

## Data Availability

The governance of the BPLTTC was reported previously^37^. Individual participant data used in this study were contributed to the BPLTTC by the original trial custodians under data-sharing agreements and cannot be deposited in a public repository or redistributed by the BPLTTC because they are subject to confidentiality and data governance restrictions. Scientific activities based on the BPLTTC dataset are overseen by the BPLTTC steering committee. Requests for data should be made directly to the data custodians for each trial. Information about individual projects is posted at https://www.wrh.ox.ac.uk/research/Blood_Pressure_Lowering_Treatment_Trialists_Collaboration_BPLTTC.

## Extended data

**Extended Data Table 1:** Time-dependent hazard ratios per one-unit increase in log-transformed follow-up time (year) for outcomes

Outcome	Events (n)	Time-Varying HR (95% CI)	P-value for Trend
**Major cardiovascular events**	43,476	1.014 (0.998–1.030)	0.095
**Ischaemic heart disease**	21,548	1.004 (0.982–1.027)	0.704
**Stroke**	14,872	0.992 (0.966–1.018)	0.524
**Heart failure**	8,086	1.108 (1.064–1.153)	1.21 × 10^−5^
**Cardiovascular death**	12,311	1.000 (0.965–1.037)	0.992
**All-cause death**	30,264	1.015 (0.990–1.041)	0.236

Time-dependent hazard ratios (HRs) and 95% confidence intervals are shown for each outcome per one-unit increase in log-transformed follow-up time (years). Estimates were derived from trial-stratified Cox proportional hazards models in which the treatment effect was allowed to vary according to log-transformed follow-up time. The reported HR describes the change in the treatment effect for each one-unit increase in log-transformed follow-up time: values > 1 indicate attenuation of relative treatment benefit over time, whereas values < 1 indicate progressively greater relative treatment benefit over time. Events denote the number of observed outcome events included in each model. *P* values for trend were calculated using two-sided Wald tests; for heart failure, *P* = 1.21 × 10^−5^. No adjustment was made for multiple comparisons.

**Extended Data Table 2:** Baseline characteristics before and after inverse probability of censoring weighting, by follow-up interval

Variable	0–1 year	1–2 years	2–3 years	3–4 years	4–5 years	>5 years
**N**	335,299	314,372	289,732	244,794	182,542	81,759
**Unweighted (Before IPCW)**						
**Age (years)**	65.0 (9.8)	64.9 (9.7)	64.7 (9.7)	64.4 (9.6)	63.7 (9.6)	62.1 (10.4)
**Women**	40.7% (136,632)	40.8% (128,128)	40.8% (118,137)	40.3% (98,673)	39.4% (71,937)	41.4% (33,876)
**Systolic blood pressure (mm Hg)**	152.4 (21.5)	152.2 (21.4)	152.1 (21.4)	152.4 (21.6)	152.0 (21.9)	155.5 (21.9)
**Body-mass index (kg/m** ^**2**^ **)**	28.1 (7.3)	28.1 (7.4)	28.1 (7.5)	28.1 (5.1)	28.3 (5.1)	28.6 (5.1)
**History of cardiovascular disease**	45.3% (151,827)	45.3% (142,462)	44.7% (129,644)	41.1% (100,661)	40.5% (73,911)	27.2% (22,276)
**History of diabetes**	28.0% (94,006)	27.8% (87,482)	27.7% (80,151)	27.8% (68,010)	28.5% (51,996)	24.6% (20,084)
**Prior antihypertensive use**	64.6% (216,649)	64.8% (203,610)	64.9% (188,080)	64.8% (158,715)	66.4% (121,137)	59.7% (48,811)
**Weighted (After IPCW)**						
**Age (years)**	65.0 (9.8)	65.0 (9.7)	65.0 (9.6)	65.0 (9.6)	65.0 (9.5)	65.3 (9.7)
**Female**	40.7% (136,632)	40.7% (136,618)	40.7% (136,593)	40.7% (136,222)	40.5% (135,632)	40.6% (137,228)
**Systolic blood pressure (mm Hg)**	152.4 (21.5)	152.4 (21.5)	152.4 (21.5)	152.4 (21.8)	152.5 (22.3)	152.6 (22.3)
**Body mass index (kg/m** ^**2**^ **)**	28.1 (7.3)	28.1 (7.3)	28.1 (7.4)	28.1 (5.1)	28.1 (5.0)	28.2 (4.9)
**History of cardiovascular disease**	45.3% (151,827)	45.3% (151,810)	45.3% (151,766)	45.3% (151,738)	45.3% (151,700)	46.5% (157,156)
**History of diabetes**	28.0% (94,006)	28.0% (93,979)	28.0% (93,941)	28.0% (93,711)	28.0% (93,695)	28.3% (95,743)
**Prior antihypertensive use**	64.6% (216,649)	64.6% (216,631)	64.6% (216,581)	64.7% (216,679)	64.8% (216,956)	66.5% (224,829)

Values are mean (SD) for continuous variables and percentage (n) for categorical variables before weighting. After inverse probability of censoring weighting (IPCW), values are weighted means (SDs) or weighted percentages, with weighted pseudo-counts shown in parentheses. Weighted pseudo-counts do not represent observed participant counts. N denotes the complete-case sample available for weighting in each follow-up interval.

**Extended Data Table 3:** Fixed-horizon absolute risk reduction and time-specific number needed to treat, standardised to a 5 mm Hg lower systolic blood pressure

Outcome	Time horizon	Standardised absolute risk reduction, % (95% CI)	Number needed to treat	Included trials
**Major cardiovascular events**	0–1 year	0.30 (0.20 to 0.40)	337	50
	0–2 years	0.59 (0.45 to 0.74)	169	49
	0–3 years	0.74 (0.57 to 0.91)	135	47
	0–4 years	1.11 (0.88 to 1.33)	91	46
	0–5 years	1.25 (0.94 to 1.57)	80	36
**Stroke**	0–1 year	0.07 (0.02 to 0.12)	1535	50
	0–2 years	0.17 (0.10 to 0.24)	596	49
	0–3 years	0.26 (0.17 to 0.35)	385	47
	0–4 years	0.38 (0.27 to 0.50)	261	46
	0–5 years	0.52 (0.36 to 0.68)	192	36
**Ischaemic heart disease**	0–1 year	0.05 (−0.02 to 0.11)	—	48
	0–2 years	0.16 (0.07 to 0.24)	642	47
	0–3 years	0.19 (0.08 to 0.29)	531	46
	0–4 years	0.25 (0.12 to 0.38)	394	45
	0–5 years	0.20 (0.03 to 0.38)	497	35
**Heart failure**	0–1 year	0.08 (0.04 to 0.12)	1312	39
	0–2 years	0.07 (0.02 to 0.12)	1530	40
	0–3 years	0.06 (−0.00 to 0.11)	—	39
	0–4 years	0.10 (0.02 to 0.17)	1057	38
	0–5 years	0.05 (−0.03 to 0.13)	—	30
**Cardiovascular death**	0–1 year	0.04 (−0.00 to 0.08)	—	47
	0–2 years	0.11 (0.04 to 0.17)	925	46
	0–3 years	0.16 (0.07 to 0.24)	633	45
	0–4 years	0.20 (0.09 to 0.32)	493	44
	0–5 years	0.29 (0.12 to 0.47)	342	34
**All-cause death**	0–1 year	0.10 (0.04 to 0.17)	987	49
	0–2 years	0.17 (0.07 to 0.27)	578	48
	0–3 years	0.27 (0.13 to 0.40)	375	46
	0–4 years	0.38 (0.20 to 0.55)	266	45
	0–5 years	0.36 (0.11 to 0.62)	275	35

Absolute risk reductions are shown as percentage points with 95% confidence intervals. Fixed-horizon risks were estimated at 1, 2, 3, 4 and 5 years. Kaplan–Meier estimates were used for all-cause death, and cumulative incidence functions accounting for death as a competing event were used for non-fatal outcomes. Trial-specific absolute risk reductions were pooled using two-stage fixed-effect meta-analysis and standardised to a 5 mm Hg lower systolic blood pressure using trial-level between-group systolic blood pressure differences. The number needed to treat was calculated as the inverse of the pooled absolute risk reduction when the estimate and its lower 95% confidence limit were greater than zero; otherwise it is shown as not calculated. Included trials denotes the number of trials contributing to each estimate.

## Code availability

Code used for the analyses is available upon reasonable request.
