# Supplementary material for: A meta-analysis of the long-term effects of antihypertensive therapy on the risk of major cardiovascular disease across 51 randomized trials
Source: Nat Med. 2026 Jul 1;32(8):3082–92. doi: 10.1038/s41591-026-04514-3 (PMC13472958; doi:10.1038/s41591-026-04514-3)
Supplement: Supplementary file 1 [file 41591_2026_4514_MOESM1_ESM.pdf]

nature medicine

Supplementary information https://doi.org/10.1038/s41591-026-04514-3

A meta-analysis of the long-term effects of antihypertensive therapy on the risk of major cardiovascular disease across 51 randomized trials

In the format provided by the authors and unedited

Supplementary Fig. 1. Standardised effects of systolic blood pressure-lowering treatment on outcomes, stratified by follow-up duration (excluding trials terminated early for benefit)

Follow–up period	Treatment group (n/N)	Incidence rate (%)	Control group (n/N)	Incidence rate (%)	HR (95% CI) per 5 mm Hg SBP reduction
**Major cardiovascular events**					
0–1 years follow–up	4,521 /143,681	3.1%	5,988 /163,176	3.7%	0.88 (0.85, 0.92)
1–2 years follow–up	3,730 /135,559	2.8%	4,805 /153,441	3.1%	0.89 (0.85, 0.94)
2–3 years follow–up	3,183 /125,076	2.5%	3,912 /141,958	2.8%	0.97 (0.92, 1.02)
3–4 years follow–up	2,521 /102,273	2.5%	3,331 /117,030	2.8%	0.89 (0.84, 0.94)
4–5 years follow–up	1,708 /71,977	2.4%	2,053 /84,199	2.4%	0.99 (0.93, 1.06)
>5 years follow–up	1,094 /30,311	3.6%	1,306 /34,740	3.8%	0.96 (0.89, 1.04)
All follow–up	16,757 /143,681	11.7%	21,395 /163,176	13.1%	0.92 (0.90, 0.94)
					P for trend < 0.001
**Ischaemic heart disease**					
0–1 years follow–up	1,940 /143,806	1.3%	2,644 /163,299	1.6%	0.92 (0.86, 0.98)
1–2 years follow–up	1,658 /137,573	1.2%	2,305 /156,118	1.5%	0.91 (0.84, 0.97)
2–3 years follow–up	1,521 /128,220	1.2%	1,907 /146,059	1.3%	1.02 (0.95, 1.10)
3–4 years follow–up	1,188 /105,753	1.1%	1,650 /121,626	1.4%	0.89 (0.82, 0.97)
4–5 years follow–up	844 /75,112	1.1%	1,049 /88,577	1.2%	1.02 (0.92, 1.12)
>5 years follow–up	681 /31,726	2.1%	842 /36,757	2.3%	0.93 (0.84, 1.03)
All follow–up	7,832 /143,806	5.4%	10,397 /163,299	6.4%	0.94 (0.91, 0.97)
					P for trend = 0.183
**Stroke**					
0–1 years follow–up	1,761 /143,794	1.2%	2,213 /163,281	1.4%	0.89 (0.83, 0.95)
1–2 years follow–up	1,329 /137,570	1.0%	1,663 /156,308	1.1%	0.85 (0.79, 0.93)
2–3 years follow–up	1,000 /128,501	0.8%	1,312 /146,764	0.9%	0.84 (0.76, 0.92)
3–4 years follow–up	797 /106,669	0.7%	1,066 /122,969	0.9%	0.85 (0.77, 0.95)
4–5 years follow–up	569 /76,499	0.7%	723 /90,441	0.8%	0.93 (0.83, 1.04)
>5 years follow–up	379 /32,802	1.2%	437 /38,167	1.1%	1.02 (0.89, 1.18)
All follow–up	5,835 /143,794	4.1%	7,414 /163,281	4.5%	0.88 (0.84, 0.91)
					P for trend = 0.077
**Heart failure**					
0–1 years follow–up	708 /128,956	0.5%	1,148 /148,848	0.8%	0.76 (0.66, 0.87)
1–2 years follow–up	673 /123,778	0.5%	866 /142,931	0.6%	0.91 (0.79, 1.05)
2–3 years follow–up	639 /115,503	0.6%	832 /134,270	0.6%	1.00 (0.87, 1.14)
3–4 years follow–up	586 /94,666	0.6%	768 /111,442	0.7%	0.93 (0.81, 1.06)
4–5 years follow–up	355 /67,111	0.5%	488 /81,565	0.6%	0.95 (0.81, 1.12)
>5 years follow–up	194 /26,370	0.7%	230 /32,100	0.7%	1.07 (0.87, 1.31)
All follow–up	3,155 /128,956	2.4%	4,332 /148,848	2.9%	0.91 (0.86, 0.96)
					P for trend < 0.001
**Cardiovascular death**					
0–1 years follow–up	919 /142,526	0.6%	1,128 /162,380	0.7%	0.92 (0.83, 1.02)
1–2 years follow–up	978 /137,713	0.7%	1,231 /157,202	0.8%	0.91 (0.82, 1.01)
2–3 years follow–up	963 /129,647	0.7%	1,171 /148,924	0.8%	1.01 (0.91, 1.12)
3–4 years follow–up	862 /108,252	0.8%	1,084 /125,659	0.9%	0.96 (0.87, 1.07)
4–5 years follow–up	612 /77,742	0.8%	770 /92,862	0.8%	0.98 (0.87, 1.10)
>5 years follow–up	485 /33,174	1.5%	572 /39,316	1.5%	1.05 (0.93, 1.18)
All follow–up	4,819 /142,526	3.4%	5,956 /162,380	3.7%	0.96 (0.92, 1.00)
					P for trend = 0.062
**All–cause death**					
0–1 years follow–up	1,977 /143,811	1.4%	2,427 /163,284	1.5%	0.93 (0.86, 1.00)
1–2 years follow–up	2,488 /138,979	1.8%	2,971 /158,094	1.9%	0.98 (0.91, 1.05)
2–3 years follow–up	2,534 /130,689	1.9%	3,086 /149,566	2.1%	1.00 (0.94, 1.07)
3–4 years follow–up	2,293 /108,980	2.1%	2,831 /126,026	2.2%	0.97 (0.91, 1.04)
4–5 years follow–up	1,568 /78,449	2.0%	1,978 /93,222	2.1%	0.99 (0.92, 1.07)
>5 years follow–up	1,149 /33,863	3.4%	1,302 /39,662	3.3%	1.04 (0.96, 1.13)
All follow–up	12,009 /143,811	8.4%	14,595 /163,284	8.9%	0.98 (0.95, 1.00)
					P for trend = 0.026

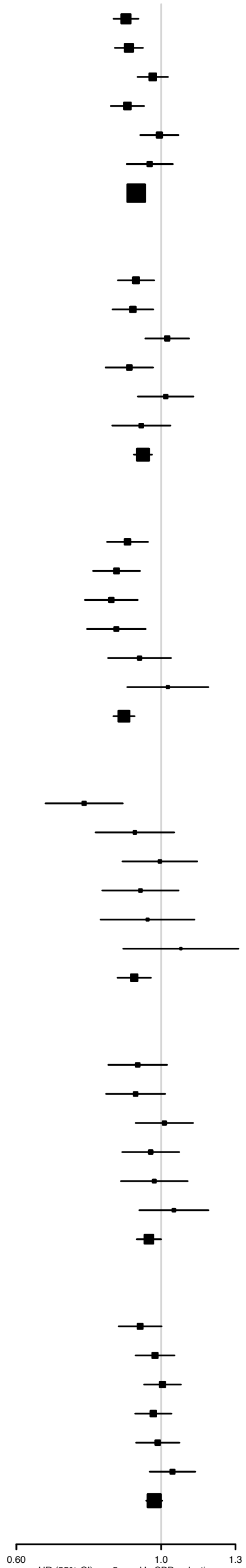

ANBP, ASCOT-BPLA, EWPHE, HOPE, HYVET, Syst-Eur, and SPRINT have been excluded from this analysis.

Supplementary Fig. 2. Standardised effects of systolic blood pressure-lowering treatment on outcomes, stratified by follow-up duration (excluding drug-comparison trials)

Follow−up period	Treatment group (n/N)	Incidence rate (%)	Control group (n/N)	Incidence rate (%)	HR (95% CI) per 5 mm Hg SBP reduction
**Major cardiovascular events**					
0−1 years follow−up	2,688 /73,786	3.6%	2,907 /71,438	4.1%	0.91 (0.86, 0.96)
1−2 years follow−up	2,011 /68,240	2.9%	2,305 /65,911	3.5%	0.86 (0.80, 0.91)
2−3 years follow−up	1,613 /60,684	2.7%	1,733 /58,579	3.0%	0.91 (0.84, 0.97)
3−4 years follow−up	1,258 /49,473	2.5%	1,464 /47,604	3.1%	0.83 (0.77, 0.90)
4−5 years follow−up	832 /35,551	2.3%	859 /34,051	2.5%	0.98 (0.88, 1.09)
>5 years follow−up	582 /14,203	4.1%	633 /13,356	4.7%	0.90 (0.75, 1.08)
All follow−up	8,984 /73,786	12.2%	9,901 /71,438	13.9%	0.89 (0.86, 0.92)
					P for trend = 0.876
**Ischaemic heart disease**					
0−1 years follow−up	1,046 /73,869	1.4%	1,066 /71,515	1.5%	0.96 (0.87, 1.05)
1−2 years follow−up	796 /69,458	1.1%	895 /67,315	1.3%	0.85 (0.77, 0.94)
2−3 years follow−up	660 /62,462	1.1%	703 /60,675	1.2%	0.90 (0.81, 1.02)
3−4 years follow−up	536 /51,323	1.0%	659 /49,779	1.3%	0.75 (0.66, 0.85)
4−5 years follow−up	334 /37,169	0.9%	350 /35,927	1.0%	0.90 (0.76, 1.07)
>5 years follow−up	338 /14,843	2.3%	368 /14,115	2.6%	0.68 (0.50, 0.93)
All follow−up	3,710 /73,869	5.0%	4,041 /71,515	5.7%	0.87 (0.83, 0.91)
					P for trend = 0.100
**Stroke**					
0−1 years follow−up	1,139 /73,863	1.5%	1,258 /71,508	1.8%	0.90 (0.83, 0.98)
1−2 years follow−up	735 /69,292	1.1%	876 /67,062	1.3%	0.86 (0.77, 0.95)
2−3 years follow−up	522 /62,411	0.8%	633 /60,519	1.0%	0.81 (0.72, 0.91)
3−4 years follow−up	353 /51,621	0.7%	445 /49,946	0.9%	0.79 (0.68, 0.92)
4−5 years follow−up	295 /37,679	0.8%	351 /36,412	1.0%	0.83 (0.68, 1.01)
>5 years follow−up	216 /15,176	1.4%	246 /14,376	1.7%	1.10 (0.78, 1.55)
All follow−up	3,260 /73,863	4.4%	3,809 /71,508	5.3%	0.86 (0.82, 0.90)
					P for trend = 0.621
**Heart failure**					
0−1 years follow−up	364 /69,886	0.5%	418 /67,851	0.6%	0.82 (0.71, 0.94)
1−2 years follow−up	324 /65,999	0.5%	368 /64,154	0.6%	0.83 (0.71, 0.97)
2−3 years follow−up	298 /59,530	0.5%	312 /58,068	0.5%	0.93 (0.79, 1.09)
3−4 years follow−up	257 /49,066	0.5%	272 /47,867	0.6%	0.94 (0.79, 1.12)
4−5 years follow−up	136 /35,955	0.4%	148 /35,134	0.4%	0.92 (0.73, 1.18)
>5 years follow−up	77 /14,239	0.5%	82 /13,856	0.6%	1.08 (0.73, 1.60)
All follow−up	1,456 /69,886	2.1%	1,600 /67,851	2.4%	0.88 (0.82, 0.95)
					P for trend = 0.074
**Cardiovascular death**					
0−1 years follow−up	558 /72,493	0.8%	575 /70,480	0.8%	0.98 (0.87, 1.10)
1−2 years follow−up	542 /68,868	0.8%	590 /67,105	0.9%	0.90 (0.80, 1.02)
2−3 years follow−up	504 /62,592	0.8%	527 /61,294	0.9%	0.92 (0.81, 1.04)
3−4 years follow−up	468 /52,046	0.9%	515 /50,986	1.0%	0.89 (0.79, 1.02)
4−5 years follow−up	296 /37,872	0.8%	299 /37,155	0.8%	1.05 (0.88, 1.24)
>5 years follow−up	235 /14,959	1.6%	270 /14,608	1.8%	0.90 (0.68, 1.20)
All follow−up	2,603 /72,493	3.6%	2,776 /70,480	3.9%	0.94 (0.89, 0.99)
					P for trend = 0.903
**All−cause death**					
0−1 years follow−up	1,065 /73,681	1.4%	1,120 /71,269	1.6%	0.94 (0.86, 1.02)
1−2 years follow−up	1,256 /70,071	1.8%	1,260 /67,925	1.9%	0.98 (0.91, 1.06)
2−3 years follow−up	1,205 /63,612	1.9%	1,288 /61,895	2.1%	0.91 (0.84, 0.99)
3−4 years follow−up	1,081 /52,765	2.0%	1,202 /51,331	2.3%	0.88 (0.80, 0.95)
4−5 years follow−up	677 /38,575	1.8%	653 /37,501	1.7%	1.05 (0.93, 1.17)
>5 years follow−up	614 /15,648	3.9%	627 /14,950	4.2%	0.91 (0.76, 1.08)
All follow−up	5,898 /73,681	8.0%	6,150 /71,269	8.6%	0.94 (0.91, 0.98)
					P for trend = 0.793

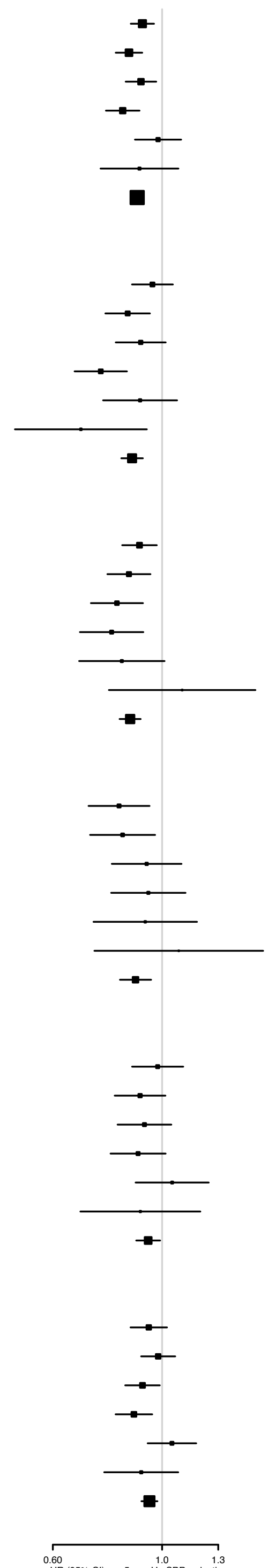

Supplementary Fig. 3. Unstandardised effects of systolic blood pressure-lowering treatment on outcomes, stratified by follow-up duration

Follow–up period	Treatment group (n/N)	Incidence rate (%)	Control group (n/N)	Incidence rate (%)	HR (95% CI)
**Major cardiovascular events**					
0–1 years follow–up	5,139 /168,980	3.0%	6,717 /188,334	3.6%	0.89 (0.85, 0.92)
1–2 years follow–up	4,238 /158,669	2.7%	5,464 /176,296	3.1%	0.91 (0.87, 0.95)
2–3 years follow–up	3,642 /146,108	2.5%	4,490 /162,675	2.8%	0.95 (0.91, 1.00)
3–4 years follow–up	2,909 /120,181	2.4%	3,822 /134,429	2.8%	0.90 (0.85, 0.94)
4–5 years follow–up	1,991 /86,599	2.3%	2,373 /98,349	2.4%	0.99 (0.94, 1.06)
>5 years follow–up	1,223 /39,286	3.1%	1,468 /43,392	3.4%	0.93 (0.86, 1.00)
All follow–up	19,142 /168,980	11.3%	24,334 /188,334	12.9%	0.92 (0.90, 0.94)
					P for trend = 0.018
**Ischaemic heart disease**					
0–1 years follow–up	2,339 /169,163	1.4%	3,056 /188,516	1.6%	0.93 (0.88, 0.98)
1–2 years follow–up	1,977 /160,844	1.2%	2,689 /179,195	1.5%	0.91 (0.85, 0.96)
2–3 years follow–up	1,806 /149,494	1.2%	2,270 /167,121	1.4%	0.99 (0.93, 1.05)
3–4 years follow–up	1,456 /123,965	1.2%	1,977 /139,429	1.4%	0.89 (0.84, 0.96)
4–5 years follow–up	1,029 /90,027	1.1%	1,253 /103,136	1.2%	1.01 (0.92, 1.09)
>5 years follow–up	756 /40,936	1.8%	940 /45,706	2.1%	0.90 (0.82, 0.99)
All follow–up	9,363 /169,163	5.5%	12,185 /188,516	6.5%	0.93 (0.91, 0.96)
					P for trend = 0.650
**Stroke**					
0–1 years follow–up	1,932 /169,160	1.1%	2,447 /188,507	1.3%	0.88 (0.82, 0.93)
1–2 years follow–up	1,472 /161,012	0.9%	1,884 /179,532	1.0%	0.89 (0.83, 0.95)
2–3 years follow–up	1,150 /150,045	0.8%	1,493 /168,084	0.9%	0.87 (0.81, 0.94)
3–4 years follow–up	899 /125,199	0.7%	1,211 /141,116	0.9%	0.87 (0.80, 0.95)
4–5 years follow–up	648 /91,790	0.7%	817 /105,395	0.8%	0.92 (0.83, 1.02)
>5 years follow–up	425 /42,299	1.0%	494 /47,394	1.0%	0.96 (0.84, 1.09)
All follow–up	6,526 /169,160	3.9%	8,346 /188,507	4.4%	0.89 (0.86, 0.92)
					P for trend = 0.314
**Heart failure**					
0–1 years follow–up	764 /154,167	0.5%	1,239 /173,894	0.7%	0.74 (0.68, 0.81)
1–2 years follow–up	731 /147,135	0.5%	933 /166,069	0.6%	0.96 (0.87, 1.05)
2–3 years follow–up	682 /137,036	0.5%	900 /155,600	0.6%	0.94 (0.85, 1.03)
3–4 years follow–up	628 /113,277	0.6%	820 /129,700	0.6%	0.95 (0.85, 1.05)
4–5 years follow–up	380 /82,518	0.5%	528 /96,707	0.5%	0.92 (0.80, 1.05)
>5 years follow–up	218 /36,019	0.6%	263 /41,515	0.6%	1.04 (0.86, 1.24)
All follow–up	3,403 /154,167	2.2%	4,683 /173,894	2.7%	0.89 (0.85, 0.93)
					P for trend < 0.001
**Cardiovascular death**					
0–1 years follow–up	1,064 /167,790	0.6%	1,286 /187,485	0.7%	0.95 (0.87, 1.03)
1–2 years follow–up	1,116 /161,210	0.7%	1,410 /180,514	0.8%	0.92 (0.85, 1.00)
2–3 years follow–up	1,090 /151,359	0.7%	1,336 /170,493	0.8%	0.97 (0.89, 1.05)
3–4 years follow–up	992 /127,055	0.8%	1,252 /144,169	0.9%	0.95 (0.87, 1.03)
4–5 years follow–up	694 /93,331	0.7%	903 /108,219	0.8%	0.95 (0.86, 1.05)
>5 years follow–up	526 /42,910	1.2%	642 /48,834	1.3%	0.98 (0.87, 1.10)
All follow–up	5,482 /167,790	3.3%	6,829 /187,485	3.6%	0.95 (0.92, 0.98)
					P for trend = 0.563
**All–cause death**					
0–1 years follow–up	2,231 /168,978	1.3%	2,714 /188,274	1.4%	0.95 (0.90, 1.00)
1–2 years follow–up	2,812 /162,413	1.7%	3,328 /181,334	1.8%	0.99 (0.94, 1.04)
2–3 years follow–up	2,854 /152,379	1.9%	3,515 /171,094	2.1%	0.96 (0.92, 1.01)
3–4 years follow–up	2,644 /127,774	2.1%	3,234 /144,514	2.2%	0.99 (0.94, 1.04)
4–5 years follow–up	1,844 /94,034	2.0%	2,275 /108,565	2.1%	1.01 (0.95, 1.07)
>5 years follow–up	1,329 /43,599	3.0%	1,484 /49,176	3.0%	1.02 (0.95, 1.10)
All follow–up	13,714 /168,978	8.1%	16,550 /188,274	8.8%	0.98 (0.96, 1.01)
					P for trend = 0.108

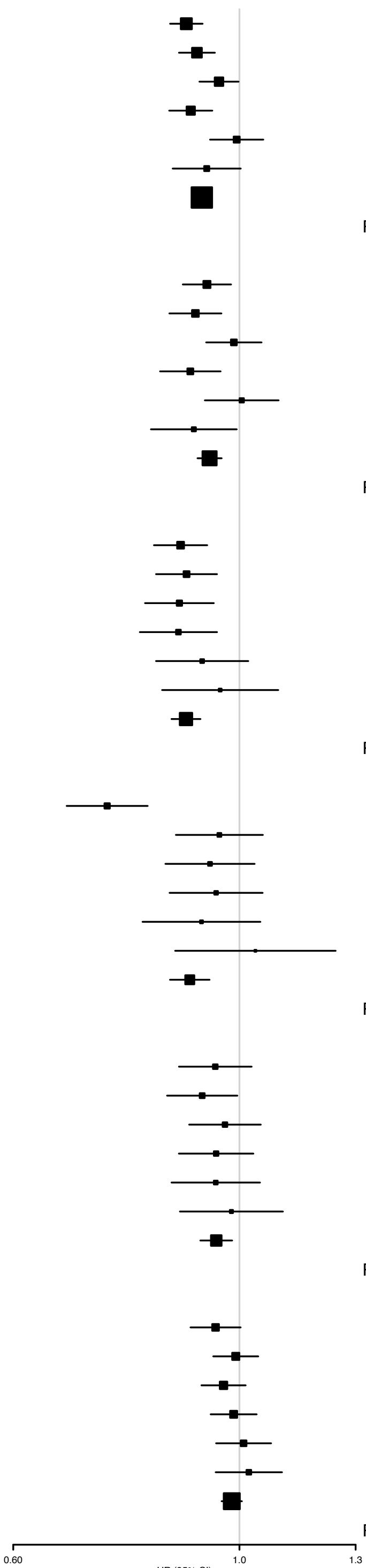

Supplementary Fig. 4. Standardised effects of systolic blood pressure-lowering treatment on outcomes, stratified by follow-up duration (standardised by blood pressure reduction in each time interval)

Follow−up period	Treatment group (n/N)	Incidence rate (%)	Control group (n/N)	Incidence rate (%)	HR (95% CI) per 5 mm Hg SBP reduction
**Major cardiovascular events**					
0−1 years follow−up	5,139 /168,980	3.0%	6,717 /188,334	3.6%	0.90 (0.86, 0.95)
1−2 years follow−up	4,238 /158,669	2.7%	5,464 /176,296	3.1%	0.86 (0.81, 0.92)
2−3 years follow−up	3,642 /146,108	2.5%	4,490 /162,675	2.8%	0.91 (0.84, 0.98)
3−4 years follow−up	2,909 /120,181	2.4%	3,822 /134,429	2.8%	0.83 (0.77, 0.90)
4−5 years follow−up	1,991 /86,599	2.3%	2,373 /98,349	2.4%	0.96 (0.87, 1.07)
>5 years follow−up	1,223 /39,286	3.1%	1,468 /43,392	3.4%	0.87 (0.77, 0.97)
All follow−up	19,142 /168,980	11.3%	24,334 /188,334	12.9%	0.90 (0.88, 0.92)
					Beta=−0.001, P=0.940
**Ischaemic heart disease**					
0−1 years follow−up	2,339 /169,163	1.4%	3,056 /188,516	1.6%	0.94 (0.86, 1.02)
1−2 years follow−up	1,977 /160,844	1.2%	2,689 /179,195	1.5%	0.85 (0.76, 0.95)
2−3 years follow−up	1,806 /149,494	1.2%	2,270 /167,121	1.4%	0.90 (0.80, 1.01)
3−4 years follow−up	1,456 /123,965	1.2%	1,977 /139,429	1.4%	0.76 (0.67, 0.85)
4−5 years follow−up	1,029 /90,027	1.1%	1,253 /103,136	1.2%	0.90 (0.76, 1.06)
>5 years follow−up	756 /40,936	1.8%	940 /45,706	2.1%	0.80 (0.68, 0.94)
All follow−up	9,363 /169,163	5.5%	12,185 /188,516	6.5%	0.92 (0.90, 0.95)
					Beta=−0.028, P=0.197
**Stroke**					
0−1 years follow−up	1,932 /169,160	1.1%	2,447 /188,507	1.3%	0.90 (0.83, 0.97)
1−2 years follow−up	1,472 /161,012	0.9%	1,884 /179,532	1.0%	0.87 (0.78, 0.96)
2−3 years follow−up	1,150 /150,045	0.8%	1,493 /168,084	0.9%	0.82 (0.72, 0.92)
3−4 years follow−up	899 /125,199	0.7%	1,211 /141,116	0.9%	0.79 (0.68, 0.91)
4−5 years follow−up	648 /91,790	0.7%	817 /105,395	0.8%	0.81 (0.67, 0.99)
>5 years follow−up	425 /42,299	1.0%	494 /47,394	1.0%	0.90 (0.74, 1.09)
All follow−up	6,526 /169,160	3.9%	8,346 /188,507	4.4%	0.87 (0.84, 0.90)
					Beta=−0.018, P=0.243
**Heart failure**					
0−1 years follow−up	764 /154,167	0.5%	1,239 /173,894	0.7%	0.82 (0.71, 0.95)
1−2 years follow−up	731 /147,135	0.5%	933 /166,069	0.6%	0.83 (0.72, 0.97)
2−3 years follow−up	682 /137,036	0.5%	900 /155,600	0.6%	0.93 (0.79, 1.10)
3−4 years follow−up	628 /113,277	0.6%	820 /129,700	0.6%	0.93 (0.78, 1.11)
4−5 years follow−up	380 /82,518	0.5%	528 /96,707	0.5%	0.91 (0.71, 1.15)
>5 years follow−up	218 /36,019	0.6%	263 /41,515	0.6%	1.02 (0.74, 1.42)
All follow−up	3,403 /154,167	2.2%	4,683 /173,894	2.7%	0.87 (0.83, 0.91)
					Beta=0.040, P=0.019
**Cardiovascular death**					
0−1 years follow−up	1,064 /167,790	0.6%	1,286 /187,485	0.7%	0.98 (0.87, 1.10)
1−2 years follow−up	1,116 /161,210	0.7%	1,410 /180,514	0.8%	0.91 (0.81, 1.03)
2−3 years follow−up	1,090 /151,359	0.7%	1,336 /170,493	0.8%	0.92 (0.81, 1.05)
3−4 years follow−up	992 /127,055	0.8%	1,252 /144,169	0.9%	0.89 (0.79, 1.01)
4−5 years follow−up	694 /93,331	0.7%	903 /108,219	0.8%	1.02 (0.87, 1.21)
>5 years follow−up	526 /42,910	1.2%	642 /48,834	1.3%	0.86 (0.72, 1.04)
All follow−up	5,482 /167,790	3.3%	6,829 /187,485	3.6%	0.93 (0.89, 0.96)
					Beta=−0.010, P=0.552
**All−cause death**					
0−1 years follow−up	2,231 /168,978	1.3%	2,714 /188,274	1.4%	0.94 (0.86, 1.03)
1−2 years follow−up	2,812 /162,413	1.7%	3,328 /181,334	1.8%	0.99 (0.91, 1.07)
2−3 years follow−up	2,854 /152,379	1.9%	3,515 /171,094	2.1%	0.91 (0.84, 0.99)
3−4 years follow−up	2,644 /127,774	2.1%	3,234 /144,514	2.2%	0.88 (0.81, 0.95)
4−5 years follow−up	1,844 /94,034	2.0%	2,275 /108,565	2.1%	1.04 (0.93, 1.16)
>5 years follow−up	1,329 /43,599	3.0%	1,484 /49,176	3.0%	0.90 (0.80, 1.01)
All follow−up	13,714 /168,978	8.1%	16,550 /188,274	8.8%	0.96 (0.94, 0.99)
					Beta=−0.005, P=0.792

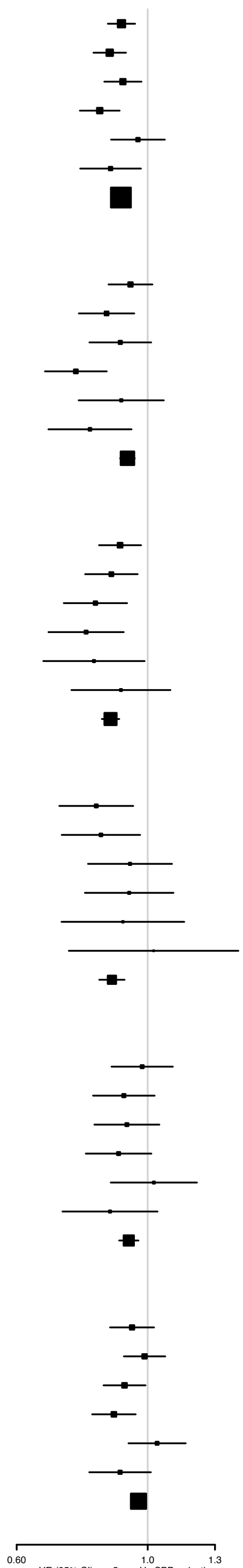

P for trend was calculated using meta-regression. For intervals in which fewer than 5% of participants had blood pressure measurements, we substituted the blood pressure reduction with that from the previous interval. A positive Beta derived from inverse-variance weighted meta-regression implies that the estimated effect (logHR) is higher over time, i.e., the protective effect of blood pressure lowering diminishes over time.

Supplementary Fig. 5. Standardised effects of diastolic blood pressure lowering per 3 mmHg reduction on outcomes, stratified by follow-up duration

Follow-up period	Treatment group (n/N)	Incidence rate (%)	Control group (n/N)	Incidence rate (%)	HR (95% CI) per 3 mm Hg DBP reduction
**Major cardiovascular events**					
0-1 years follow-up	5,139 /161,462	3.2%	6,717 /180,661	3.7%	0.88 (0.84, 0.92)
1-2 years follow-up	4,238 /151,314	2.8%	5,464 /168,769	3.2%	0.86 (0.82, 0.90)
2-3 years follow-up	3,642 /138,913	2.6%	4,490 /155,317	2.9%	0.91 (0.86, 0.97)
3-4 years follow-up	2,909 /117,006	2.5%	3,822 /131,115	2.9%	0.83 (0.78, 0.89)
4-5 years follow-up	1,991 /86,377	2.3%	2,373 /98,124	2.4%	0.92 (0.85, 1.00)
>5 years follow-up	1,223 /39,286	3.1%	1,468 /43,392	3.4%	0.90 (0.83, 0.98)
					P for trend = 0.021
All follow-up	19,142 /161,462	11.9%	24,334 /180,661	13.5%	0.88 (0.86, 0.90)
**Ischaemic heart disease**					
0-1 years follow-up	2,339 /161,459	1.4%	3,056 /180,691	1.7%	0.92 (0.86, 0.99)
1-2 years follow-up	1,977 /153,303	1.3%	2,689 /171,518	1.6%	0.87 (0.80, 0.94)
2-3 years follow-up	1,806 /142,116	1.3%	2,270 /159,615	1.4%	0.95 (0.87, 1.03)
3-4 years follow-up	1,456 /120,676	1.2%	1,977 /136,028	1.5%	0.84 (0.76, 0.93)
4-5 years follow-up	1,029 /89,790	1.1%	1,253 /102,907	1.2%	0.92 (0.81, 1.04)
>5 years follow-up	756 /40,936	1.8%	940 /45,706	2.1%	0.88 (0.79, 0.98)
					P for trend = 0.677
All follow-up	9,363 /161,459	5.8%	12,185 /180,691	6.7%	0.90 (0.87, 0.94)
**Stroke**					
0-1 years follow-up	1,932 /161,461	1.2%	2,447 /180,638	1.4%	0.87 (0.82, 0.93)
1-2 years follow-up	1,472 /153,476	1.0%	1,884 /171,811	1.1%	0.82 (0.76, 0.89)
2-3 years follow-up	1,150 /142,671	0.8%	1,493 /160,535	0.9%	0.83 (0.76, 0.91)
3-4 years follow-up	899 /121,910	0.7%	1,211 /137,689	0.9%	0.80 (0.71, 0.90)
4-5 years follow-up	648 /91,558	0.7%	817 /105,160	0.8%	0.88 (0.78, 0.99)
>5 years follow-up	425 /42,299	1.0%	494 /47,394	1.0%	0.92 (0.81, 1.06)
					P for trend = 0.305
All follow-up	6,526 /161,461	4.0%	8,346 /180,638	4.6%	0.85 (0.82, 0.88)
**Heart failure**					
0-1 years follow-up	764 /146,468	0.5%	1,239 /166,018	0.7%	0.78 (0.69, 0.88)
1-2 years follow-up	731 /139,599	0.5%	933 /158,341	0.6%	0.91 (0.80, 1.03)
2-3 years follow-up	682 /129,663	0.5%	900 /148,042	0.6%	0.93 (0.82, 1.07)
3-4 years follow-up	628 /109,992	0.6%	820 /126,271	0.6%	0.87 (0.74, 1.02)
4-5 years follow-up	380 /82,287	0.5%	528 /96,470	0.5%	0.90 (0.74, 1.10)
>5 years follow-up	218 /36,019	0.6%	263 /41,515	0.6%	0.98 (0.79, 1.21)
					P for trend < 0.001
All follow-up	3,403 /146,468	2.3%	4,683 /166,018	2.8%	0.88 (0.83, 0.93)
**Cardiovascular death**					
0-1 years follow-up	1,064 /159,988	0.7%	1,286 /179,530	0.7%	0.91 (0.82, 1.00)
1-2 years follow-up	1,116 /153,571	0.7%	1,410 /172,708	0.8%	0.89 (0.80, 0.98)
2-3 years follow-up	1,090 /143,884	0.8%	1,336 /162,859	0.8%	0.95 (0.86, 1.06)
3-4 years follow-up	992 /123,704	0.8%	1,252 /140,691	0.9%	0.90 (0.80, 1.00)
4-5 years follow-up	694 /93,092	0.7%	903 /107,981	0.8%	0.87 (0.76, 1.00)
>5 years follow-up	526 /42,910	1.2%	642 /48,834	1.3%	0.93 (0.82, 1.05)
					P for trend = 0.495
All follow-up	5,482 /159,988	3.4%	6,829 /179,530	3.8%	0.91 (0.87, 0.95)
**All-cause death**					
0-1 years follow-up	2,231 /161,176	1.4%	2,714 /180,319	1.5%	0.92 (0.86, 0.98)
1-2 years follow-up	2,812 /154,774	1.8%	3,328 /173,528	1.9%	0.96 (0.90, 1.02)
2-3 years follow-up	2,854 /144,904	2.0%	3,515 /163,460	2.2%	0.92 (0.86, 0.99)
3-4 years follow-up	2,644 /124,423	2.1%	3,234 /141,036	2.3%	0.91 (0.84, 0.97)
4-5 years follow-up	1,844 /93,795	2.0%	2,275 /108,327	2.1%	0.97 (0.88, 1.06)
>5 years follow-up	1,329 /43,599	3.0%	1,484 /49,176	3.0%	0.99 (0.91, 1.07)
					P for trend = 0.090
All follow-up	13,714 /161,176	8.5%	16,550 /180,319	9.2%	0.94 (0.91, 0.97)

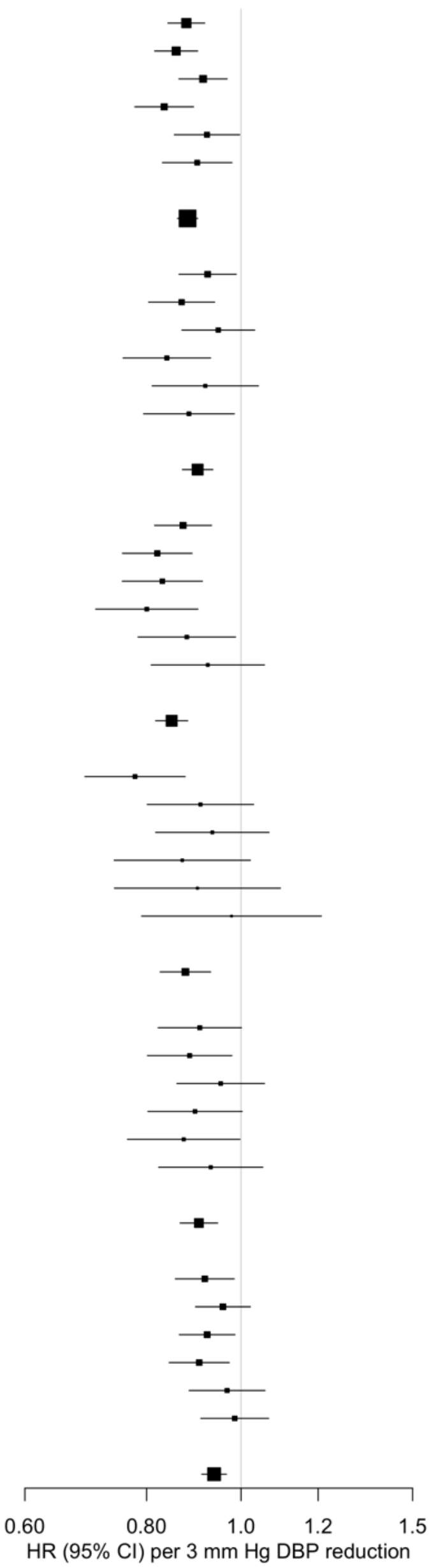

Supplementary Fig. 6. Standardised effects of systolic blood pressure-lowering treatment on ischaemic heart disease, stratified by follow-up duration and age

Follow−up period	Treatment group (n/N)	Incidence (%)	Control group (n/N)	Incidence (%)	HR (95% CI) per 5 mm Hg SBP reduction
**Aged <55 years**					
0−1 years follow−up	222/21,243	1.0%	230/20,331	1.1%	0.90 (0.74, 1.10)
1−2 years follow−up	165/20,312	0.8%	187/19,495	1.0%	0.81 (0.65, 1.01)
2−3 years follow−up	157/18,972	0.8%	164/18,246	0.9%	0.90 (0.71, 1.14)
3−4 years follow−up	99/16,489	0.6%	136/15,909	0.9%	0.71 (0.54, 0.92)
4−5 years follow−up	91/13,146	0.7%	98/12,566	0.8%	0.91 (0.66, 1.23)
>5 years follow−up	164/8,442	1.9%	163/8,195	2.0%	1.04 (0.76, 1.44)
All follow−up	898/21,243	4.2%	978/20,331	4.8%	0.86 (0.78, 0.95)
					P for trend = 0.817
**Aged 55−64 years**					
0−1 years follow−up	739/58,862	1.3%	969/66,018	1.5%	0.89 (0.81, 0.98)
1−2 years follow−up	658/56,449	1.2%	834/63,202	1.3%	0.98 (0.88, 1.10)
2−3 years follow−up	593/52,909	1.1%	749/59,436	1.3%	0.97 (0.86, 1.09)
3−4 years follow−up	464/43,798	1.1%	615/49,501	1.2%	0.89 (0.78, 1.01)
4−5 years follow−up	334/33,154	1.0%	406/38,031	1.1%	0.98 (0.84, 1.15)
>5 years follow−up	291/15,331	1.9%	317/16,862	1.9%	0.97 (0.83, 1.15)
All follow−up	3,079/58,862	5.2%	3,890/66,018	5.9%	0.94 (0.90, 0.99)
					P for trend = 0.551
**Aged 65−74 years**					
0−1 years follow−up	946/60,382	1.6%	1,209/68,799	1.8%	1.00 (0.91, 1.10)
1−2 years follow−up	769/57,345	1.3%	1,076/65,459	1.6%	0.88 (0.79, 0.97)
2−3 years follow−up	705/53,641	1.3%	923/61,446	1.5%	0.93 (0.83, 1.04)
3−4 years follow−up	599/44,931	1.3%	824/51,669	1.6%	0.88 (0.78, 0.99)
4−5 years follow−up	426/31,831	1.3%	500/37,615	1.3%	1.01 (0.87, 1.18)
>5 years follow−up	222/12,431	1.8%	327/14,712	2.2%	0.82 (0.68, 0.97)
All follow−up	3,667/60,382	6.1%	4,859/68,799	7.1%	0.93 (0.88, 0.97)
					P for trend = 0.804
**Aged 75−84 years**					
0−1 years follow−up	391/26,123	1.5%	578/30,480	1.9%	0.87 (0.76, 1.00)
1−2 years follow−up	353/24,480	1.4%	527/28,518	1.8%	0.83 (0.72, 0.97)
2−3 years follow−up	318/22,170	1.4%	404/25,953	1.6%	0.99 (0.82, 1.18)
3−4 years follow−up	263/17,650	1.5%	380/21,009	1.8%	0.93 (0.77, 1.11)
4−5 years follow−up	168/11,265	1.5%	229/14,124	1.6%	0.99 (0.76, 1.29)
>5 years follow−up	74/4,485	1.6%	130/5,623	2.3%	0.66 (0.45, 0.97)
All follow−up	1,567/26,123	6.0%	2,248/30,480	7.4%	0.89 (0.83, 0.96)
					P for trend = 0.839
**Aged >=85 years**					
0−1 years follow−up	41/2,539	1.6%	69/2,859	2.4%	0.73 (0.49, 1.08)
1−2 years follow−up	32/2,245	1.4%	65/2,496	2.6%	0.60 (0.37, 0.97)
2−3 years follow−up	33/1,793	1.8%	30/2,022	1.5%	1.29 (0.76, 2.20)
3−4 years follow−up	31/1,095	2.8%	22/1,335	1.6%	1.81 (1.01, 3.25)
4−5 years follow−up	10/631	1.6%	20/799	2.5%	4.57 (0.25, 84.68)
All follow−up	152/2,539	6.0%	209/2,859	7.3%	0.92 (0.73, 1.15)
					P for trend = 0.013

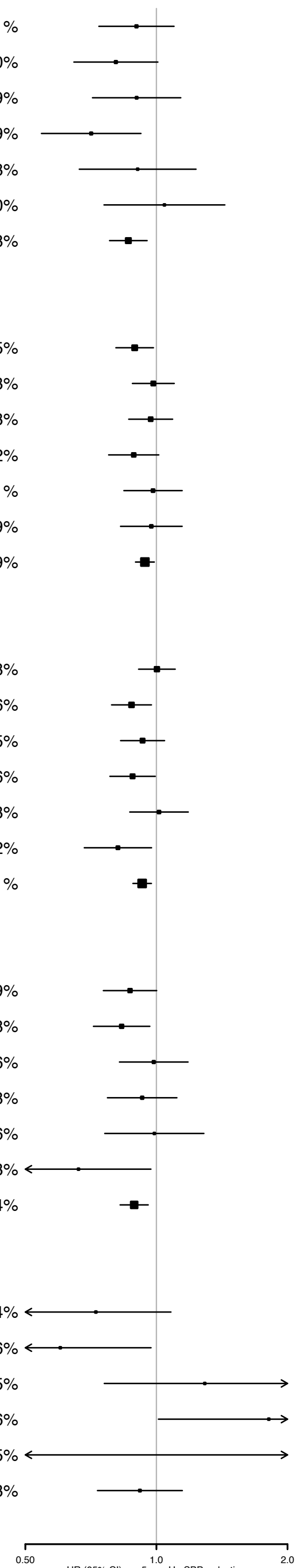

Supplementary Fig. 7. Standardised effects of systolic blood pressure-lowering treatment on stroke, stratified by follow-up duration and age

Follow−up period	Treatment group (n/N)	Incidence (%)	Control group (n/N)	Incidence (%)	HR (95% CI) per 5 mm Hg SBP reduction
**Aged <55 years**					
0−1 years follow−up	102/21,245	0.5%	140/20,330	0.7%	0.71 (0.55, 0.91)
1−2 years follow−up	74/20,401	0.4%	99/19,546	0.5%	0.72 (0.53, 0.97)
2−3 years follow−up	58/19,139	0.3%	81/18,375	0.4%	0.73 (0.52, 1.04)
3−4 years follow−up	47/16,745	0.3%	65/16,131	0.4%	0.67 (0.45, 0.98)
4−5 years follow−up	93/13,407	0.7%	126/12,815	1.0%	0.60 (0.38, 0.94)
>5 years follow−up	93/8,605	1.1%	125/8,323	1.5%	0.87 (0.57, 1.32)
All follow−up	467/21,245	2.2%	636/20,330	3.1%	0.70 (0.62, 0.80)
					P for trend = 0.844
**Aged 55−64 years**					
0−1 years follow−up	518/58,861	0.9%	638/66,014	1.0%	0.88 (0.78, 1.00)
1−2 years follow−up	361/56,576	0.6%	479/63,465	0.8%	0.77 (0.67, 0.90)
2−3 years follow−up	300/53,257	0.6%	388/59,982	0.6%	0.79 (0.67, 0.93)
3−4 years follow−up	258/44,441	0.6%	297/50,392	0.6%	0.95 (0.79, 1.13)
4−5 years follow−up	171/33,967	0.5%	189/39,105	0.5%	1.03 (0.84, 1.27)
>5 years follow−up	129/15,902	0.8%	150/17,644	0.9%	0.88 (0.69, 1.13)
All follow−up	1,737/58,861	3.0%	2,141/66,014	3.2%	0.86 (0.81, 0.92)
					P for trend = 0.159
**Aged 65−74 years**					
0−1 years follow−up	765/60,383	1.3%	991/68,798	1.4%	0.85 (0.76, 0.94)
1−2 years follow−up	595/57,417	1.0%	745/65,574	1.1%	0.86 (0.76, 0.97)
2−3 years follow−up	473/53,826	0.9%	587/61,800	0.9%	0.93 (0.82, 1.07)
3−4 years follow−up	358/45,372	0.8%	504/52,278	1.0%	0.79 (0.68, 0.92)
4−5 years follow−up	243/32,477	0.7%	314/38,476	0.8%	0.87 (0.72, 1.04)
>5 years follow−up	146/13,000	1.1%	137/15,372	0.9%	1.34 (1.05, 1.70)
All follow−up	2,580/60,383	4.3%	3,278/68,798	4.8%	0.88 (0.83, 0.94)
					P for trend = 0.033
**Aged 75−84 years**					
0−1 years follow−up	483/26,119	1.8%	600/30,478	2.0%	0.91 (0.80, 1.04)
1−2 years follow−up	397/24,373	1.6%	495/28,439	1.7%	0.89 (0.77, 1.03)
2−3 years follow−up	288/22,044	1.3%	402/25,902	1.6%	0.86 (0.73, 1.01)
3−4 years follow−up	220/17,557	1.3%	317/20,974	1.5%	0.80 (0.66, 0.98)
4−5 years follow−up	134/11,303	1.2%	178/14,196	1.3%	0.94 (0.73, 1.21)
>5 years follow−up	54/4,543	1.2%	76/5,739	1.3%	1.04 (0.68, 1.60)
All follow−up	1,576/26,119	6.0%	2,068/30,478	6.8%	0.89 (0.83, 0.95)
					P for trend = 0.791
**Aged >=85 years**					
0−1 years follow−up	63/2,538	2.5%	77/2,858	2.7%	0.89 (0.63, 1.24)
1−2 years follow−up	45/2,233	2.0%	66/2,484	2.7%	0.77 (0.53, 1.13)
2−3 years follow−up	31/1,770	1.8%	35/2,008	1.7%	0.96 (0.59, 1.57)
3−4 years follow−up	16/1,082	1.5%	28/1,335	2.1%	0.64 (0.34, 1.20)
4−5 years follow−up	7/636	1.1%	10/802	1.2%	0.99 (0.33, 2.91)
All follow−up	165/2,538	6.5%	222/2,858	7.8%	0.83 (0.68, 1.02)
					P for trend = 0.653

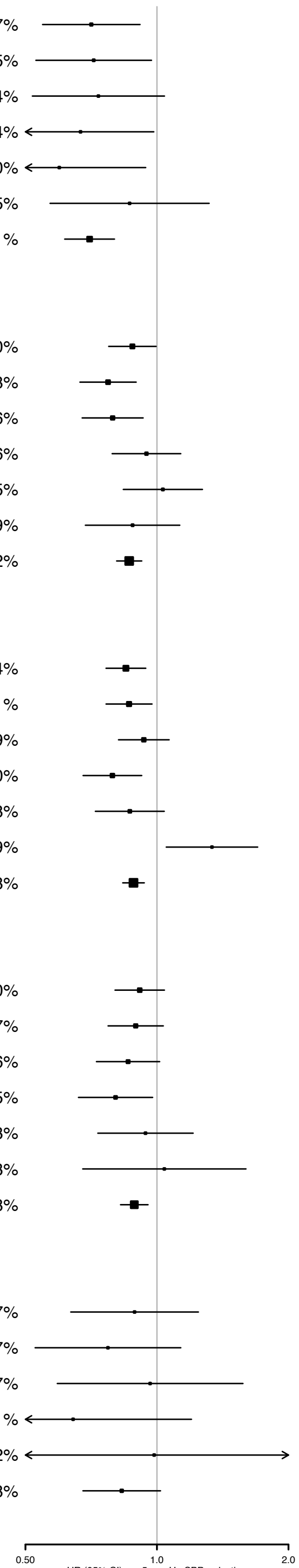

Supplementary Fig. 8. Standardised effects of systolic blood pressure-lowering treatment on heart failure, stratified by follow-up duration and age

Follow-up period	Treatment group (n/N)	Incidence (%)	Control group (n/N)	Incidence (%)	HR (95% CI) per 5 mm Hg SBP reduction
**Aged <55 years**					
0–1 years follow–up	27/16,292	0.2%	23/15,484	0.1%	1.08 (0.61, 1.92)
1–2 years follow–up	28/15,561	0.2%	29/14,872	0.2%	0.85 (0.50, 1.43)
2–3 years follow–up	22/14,425	0.2%	20/13,838	0.1%	1.03 (0.56, 1.91)
3–4 years follow–up	13/12,276	0.1%	17/11,829	0.1%	0.67 (0.30, 1.51)
4–5 years follow–up	16/9,582	0.2%	15/9,235	0.2%	0.97 (0.46, 2.06)
>5 years follow–up	15/5,755	0.3%	17/5,634	0.3%	1.21 (0.44, 3.28)
All follow–up	121/16,292	0.7%	121/15,484	0.8%	0.95 (0.74, 1.23)
					P for trend = 0.961
**Aged 55–64 years**					
0–1 years follow–up	156/53,385	0.3%	284/60,750	0.5%	0.63 (0.51, 0.79)
1–2 years follow–up	193/51,489	0.4%	199/58,543	0.3%	1.20 (0.96, 1.52)
2–3 years follow–up	166/48,429	0.3%	215/55,363	0.4%	0.97 (0.78, 1.21)
3–4 years follow–up	144/39,936	0.4%	197/46,094	0.4%	0.92 (0.72, 1.18)
4–5 years follow–up	98/30,392	0.3%	132/35,719	0.4%	1.01 (0.76, 1.34)
>5 years follow–up	50/13,402	0.4%	71/15,273	0.5%	0.80 (0.55, 1.16)
All follow–up	807/53,385	1.5%	1,098/60,750	1.8%	0.91 (0.82, 1.00)
					P for trend = 0.267
**Aged 65–74 years**					
0–1 years follow–up	342/56,821	0.6%	514/65,312	0.8%	0.84 (0.71, 0.99)
1–2 years follow–up	277/54,245	0.5%	390/62,524	0.6%	0.80 (0.65, 0.98)
2–3 years follow–up	252/50,990	0.5%	382/59,119	0.6%	0.84 (0.70, 1.01)
3–4 years follow–up	267/42,945	0.6%	355/49,992	0.7%	0.90 (0.75, 1.08)
4–5 years follow–up	175/30,960	0.6%	210/37,076	0.6%	0.95 (0.75, 1.22)
>5 years follow–up	91/12,166	0.7%	111/14,674	0.8%	1.07 (0.80, 1.42)
All follow–up	1,404/56,821	2.5%	1,962/65,312	3.0%	0.88 (0.81, 0.95)
					P for trend = 0.017
**Aged 75–84 years**					
0–1 years follow–up	194/25,211	0.8%	355/29,555	1.2%	0.62 (0.51, 0.75)
1–2 years follow–up	197/23,687	0.8%	280/27,715	1.0%	0.78 (0.64, 0.95)
2–3 years follow–up	207/21,484	1.0%	250/25,321	1.0%	0.92 (0.75, 1.14)
3–4 years follow–up	180/17,103	1.1%	228/20,514	1.1%	0.91 (0.71, 1.16)
4–5 years follow–up	83/11,001	0.8%	154/13,917	1.1%	0.68 (0.49, 0.96)
>5 years follow–up	54/4,474	1.2%	57/5,644	1.0%	1.07 (0.69, 1.64)
All follow–up	915/25,211	3.6%	1,324/29,555	4.5%	0.78 (0.71, 0.86)
					P for trend = 0.015
**Aged >=85 years**					
0–1 years follow–up	45/2,445	1.8%	63/2,765	2.3%	0.88 (0.59, 1.30)
1–2 years follow–up	36/2,141	1.7%	34/2,391	1.4%	1.21 (0.74, 1.96)
2–3 years follow–up	35/1,700	2.1%	33/1,942	1.7%	1.31 (0.81, 2.12)
3–4 years follow–up	24/1,016	2.4%	23/1,266	1.8%	1.35 (0.74, 2.47)
4–5 years follow–up	8/583	1.4%	17/760	2.2%	0.04 (0.00, 25.81)
>5 years follow–up	8/222	3.6%	7/290	2.4%	1.48 (0.53, 4.14)
All follow–up	156/2,445	6.4%	177/2,765	6.4%	1.10 (0.88, 1.37)
					P for trend = 0.506

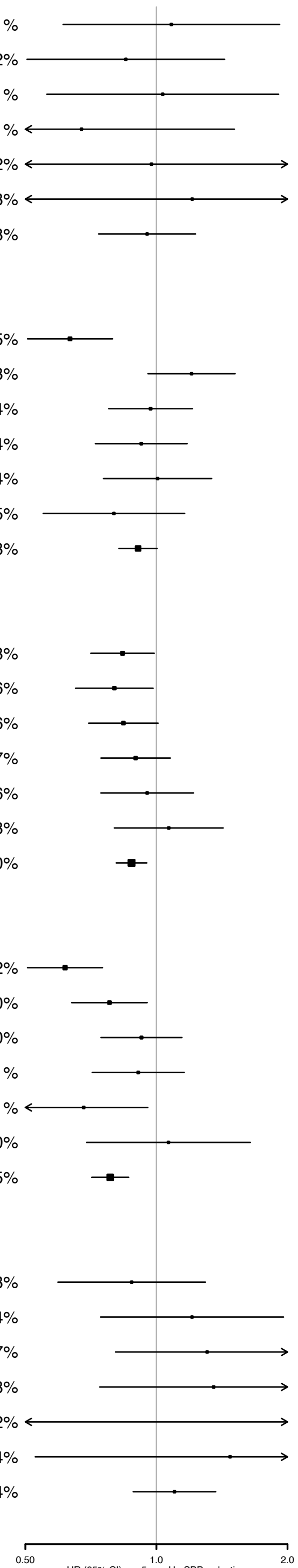

Supplementary Fig. 9. Standardised effects of systolic blood pressure-lowering treatment on cardiovascular death, stratified by follow-up duration and age

Follow−up period	Treatment group (n/N)	Incidence (%)	Control group (n/N)	Incidence (%)	HR (95% CI) per 5 mm Hg SBP reduction
**Aged <55 years**					
0−1 years follow−up	44/20,956	0.2%	55/20,190	0.3%	0.78 (0.52, 1.15)
1−2 years follow−up	47/20,195	0.2%	44/19,529	0.2%	1.01 (0.67, 1.52)
2−3 years follow−up	50/18,974	0.3%	60/18,421	0.3%	0.82 (0.56, 1.20)
3−4 years follow−up	53/16,592	0.3%	52/16,180	0.3%	1.08 (0.71, 1.64)
4−5 years follow−up	41/13,250	0.3%	38/12,880	0.3%	0.99 (0.61, 1.59)
>5 years follow−up	65/8,490	0.8%	94/8,425	1.1%	0.80 (0.45, 1.44)
All follow−up	300/20,956	1.4%	343/20,190	1.7%	0.88 (0.75, 1.04)
					P for trend = 0.910
**Aged 55−64 years**					
0−1 years follow−up	254/58,307	0.4%	283/65,623	0.4%	0.96 (0.80, 1.15)
1−2 years follow−up	261/56,458	0.5%	308/63,595	0.5%	0.88 (0.73, 1.06)
2−3 years follow−up	241/53,464	0.5%	285/60,532	0.5%	0.93 (0.77, 1.12)
3−4 years follow−up	216/44,848	0.5%	270/51,152	0.5%	0.88 (0.73, 1.06)
4−5 years follow−up	151/34,332	0.4%	193/39,806	0.5%	0.91 (0.73, 1.13)
>5 years follow−up	147/16,013	0.9%	167/17,952	0.9%	0.96 (0.76, 1.22)
All follow−up	1,270/58,307	2.2%	1,506/65,623	2.3%	0.93 (0.86, 1.01)
					P for trend = 0.805
**Aged 65−74 years**					
0−1 years follow−up	437/60,050	0.7%	496/68,512	0.7%	0.95 (0.83, 1.10)
1−2 years follow−up	439/57,693	0.8%	541/66,097	0.8%	0.87 (0.76, 1.00)
2−3 years follow−up	427/54,566	0.8%	521/62,905	0.8%	1.01 (0.88, 1.17)
3−4 years follow−up	403/46,314	0.9%	487/53,665	0.9%	0.96 (0.83, 1.11)
4−5 years follow−up	265/33,294	0.8%	341/39,764	0.9%	0.88 (0.73, 1.05)
>5 years follow−up	196/13,355	1.5%	229/16,024	1.4%	1.04 (0.86, 1.27)
All follow−up	2,167/60,050	3.6%	2,615/68,512	3.8%	0.95 (0.89, 1.01)
					P for trend = 0.451
**Aged 75−84 years**					
0−1 years follow−up	281/25,967	1.1%	380/30,326	1.3%	0.87 (0.73, 1.03)
1−2 years follow−up	295/24,607	1.2%	424/28,773	1.5%	0.84 (0.72, 1.00)
2−3 years follow−up	301/22,534	1.3%	403/26,561	1.5%	0.95 (0.80, 1.11)
3−4 years follow−up	278/18,176	1.5%	386/21,784	1.8%	1.05 (0.86, 1.27)
4−5 years follow−up	213/11,791	1.8%	305/14,934	2.0%	0.87 (0.71, 1.06)
>5 years follow−up	105/4,794	2.2%	142/6,104	2.3%	1.12 (0.85, 1.48)
All follow−up	1,473/25,967	5.7%	2,040/30,326	6.7%	0.91 (0.85, 0.98)
					P for trend = 0.336
**Aged >=85 years**					
0−1 years follow−up	48/2,496	1.9%	70/2,805	2.5%	0.75 (0.52, 1.09)
1−2 years follow−up	72/2,244	3.2%	93/2,495	3.7%	0.93 (0.67, 1.28)
2−3 years follow−up	71/1,812	3.9%	67/2,056	3.3%	1.29 (0.92, 1.81)
3−4 years follow−up	42/1,123	3.7%	57/1,382	4.1%	0.88 (0.57, 1.34)
4−5 years follow−up	24/664	3.6%	26/834	3.1%	1.22 (0.69, 2.15)
>5 years follow−up	13/258	5.0%	10/329	3.0%	1.66 (0.64, 4.35)
All follow−up	270/2,496	10.8%	323/2,805	11.5%	0.99 (0.84, 1.17)
					P for trend = 0.044

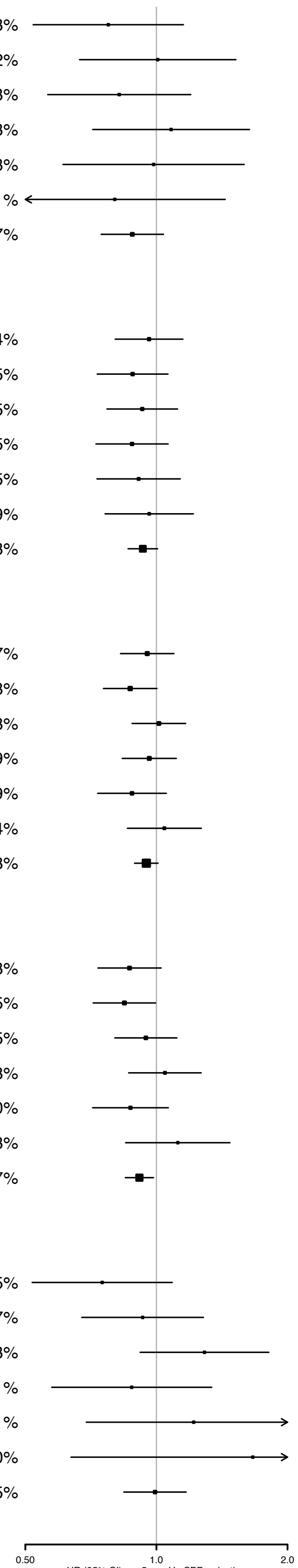

Supplementary Fig. 10. Standardised effects of systolic blood pressure-lowering treatment on all-cause death, stratified by follow-up duration and age

Follow–up period	Treatment group (n/N)	Incidence (%)	Control group (n/N)	Incidence (%)	HR (95% CI) per 5 mm Hg SBP reduction
**Aged <55 years**					
0–1 years follow–up	87/21,250	0.4%	122/20,334	0.6%	0.69 (0.52, 0.91)
1–2 years follow–up	116/20,489	0.6%	111/19,669	0.6%	1.00 (0.77, 1.30)
2–3 years follow–up	113/19,267	0.6%	127/18,561	0.7%	0.88 (0.68, 1.14)
3–4 years follow–up	120/16,882	0.7%	134/16,319	0.8%	0.88 (0.68, 1.14)
4–5 years follow–up	90/13,536	0.7%	95/13,015	0.7%	0.94 (0.69, 1.27)
>5 years follow–up	184/8,771	2.1%	221/8,555	2.6%	0.90 (0.65, 1.24)
All follow–up	710/21,250	3.3%	810/20,334	4.0%	0.87 (0.78, 0.97)
					P for trend = 0.462
**Aged 55–64 years**					
0–1 years follow–up	511/58,868	0.9%	628/66,018	1.0%	0.92 (0.81, 1.05)
1–2 years follow–up	632/57,013	1.1%	735/63,986	1.1%	0.97 (0.86, 1.10)
2–3 years follow–up	645/53,925	1.2%	764/60,818	1.3%	0.97 (0.86, 1.09)
3–4 years follow–up	555/45,167	1.2%	688/51,320	1.3%	0.90 (0.80, 1.02)
4–5 years follow–up	430/34,636	1.2%	510/39,971	1.3%	1.01 (0.88, 1.16)
>5 years follow–up	390/16,308	2.4%	401/18,111	2.2%	1.02 (0.88, 1.18)
All follow–up	3,163/58,868	5.4%	3,726/66,018	5.6%	0.96 (0.92, 1.01)
					P for trend = 0.287
**Aged 65–74 years**					
0–1 years follow–up	886/60,384	1.5%	1,034/68,797	1.5%	0.95 (0.86, 1.06)
1–2 years follow–up	1,101/58,019	1.9%	1,314/66,378	2.0%	0.94 (0.86, 1.04)
2–3 years follow–up	1,113/54,795	2.0%	1,448/63,076	2.3%	0.91 (0.83, 0.99)
3–4 years follow–up	1,094/46,433	2.4%	1,347/53,725	2.5%	0.96 (0.87, 1.05)
4–5 years follow–up	719/33,411	2.2%	890/39,824	2.2%	0.99 (0.88, 1.11)
>5 years follow–up	478/13,468	3.5%	518/16,081	3.2%	1.11 (0.97, 1.26)
All follow–up	5,391/60,384	8.9%	6,551/68,797	9.5%	0.96 (0.92, 1.00)
					P for trend = 0.145
**Aged 75–84 years**					
0–1 years follow–up	641/26,003	2.5%	779/30,346	2.6%	0.95 (0.84, 1.06)
1–2 years follow–up	806/24,653	3.3%	977/28,823	3.4%	0.98 (0.88, 1.08)
2–3 years follow–up	822/22,573	3.6%	1,004/26,588	3.8%	1.02 (0.92, 1.13)
3–4 years follow–up	765/18,170	4.2%	933/21,773	4.3%	1.10 (0.98, 1.23)
4–5 years follow–up	544/11,789	4.6%	699/14,928	4.7%	0.95 (0.83, 1.09)
>5 years follow–up	250/4,794	5.2%	314/6,101	5.1%	1.13 (0.94, 1.36)
All follow–up	3,828/26,003	14.7%	4,706/30,346	15.5%	1.01 (0.96, 1.06)
					P for trend = 0.260
**Aged >=85 years**					
0–1 years follow–up	105/2,459	4.3%	147/2,750	5.3%	0.80 (0.62, 1.03)
1–2 years follow–up	154/2,226	6.9%	188/2,453	7.7%	0.98 (0.78, 1.22)
2–3 years follow–up	160/1,810	8.8%	172/2,033	8.5%	1.13 (0.91, 1.41)
3–4 years follow–up	110/1,120	9.8%	132/1,371	9.6%	1.01 (0.78, 1.32)
4–5 years follow–up	61/662	9.2%	81/826	9.8%	1.12 (0.78, 1.61)
>5 years follow–up	27/258	10.5%	30/328	9.1%	1.03 (0.59, 1.81)
All follow–up	617/2,459	25.1%	750/2,750	27.3%	0.99 (0.89, 1.11)
					P for trend = 0.132

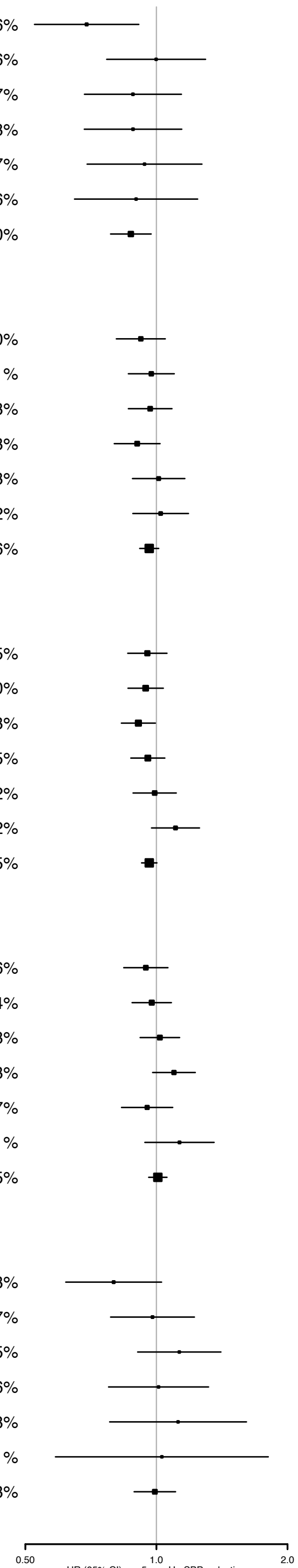

Supplementary Fig. 11. Standardised effects of systolic blood pressure treatment on outcomes among men, stratified by follow-up duration

Follow–up period	Treatment group (n/N)	Incidence (%)	Control group (n/N)	Incidence (%)	HR (95% CI) per 5 mm Hg SBP reduction
**Major cardiovascular events**					
0–1 years follow–up	3,383/98,484	3.4%	4,331/110,290	3.9%	0.89 (0.85, 0.94)
1–2 years follow–up	2,748/92,388	3.0%	3,490/103,239	3.4%	0.90 (0.85, 0.95)
2–3 years follow–up	2,321/84,977	2.7%	2,976/95,168	3.1%	0.89 (0.84, 0.95)
3–4 years follow–up	1,924/70,852	2.7%	2,495/79,790	3.1%	0.87 (0.82, 0.93)
4–5 years follow–up	1,255/52,054	2.4%	1,489/59,600	2.5%	0.96 (0.89, 1.04)
>5 years follow–up	757/22,887	3.3%	922/25,359	3.6%	0.93 (0.85, 1.03)
					P for trend = 0.111
All follow–up	12,388/98,484	12.6%	15,703/110,290	14.2%	0.90 (0.88, 0.92)
**Ischaemic heart disease**					
0–1 years follow–up	1,638/98,582	1.7%	2,097/110,383	1.9%	0.93 (0.87, 1.00)
1–2 years follow–up	1,346/93,758	1.4%	1,816/105,021	1.7%	0.91 (0.84, 0.98)
2–3 years follow–up	1,243/87,098	1.4%	1,597/97,881	1.6%	0.93 (0.85, 1.00)
3–4 years follow–up	1,023/73,197	1.4%	1,373/82,864	1.7%	0.89 (0.81, 0.97)
4–5 years follow–up	703/54,190	1.3%	836/62,542	1.3%	0.98 (0.88, 1.10)
>5 years follow–up	503/23,874	2.1%	627/26,687	2.3%	0.91 (0.81, 1.03)
					P for trend = 0.684
All follow–up	6,456/98,582	6.5%	8,346/110,383	7.6%	0.92 (0.89, 0.95)
**Stroke**					
0–1 years follow–up	1,229/98,585	1.2%	1,528/110,374	1.4%	0.89 (0.82, 0.96)
1–2 years follow–up	894/93,971	1.0%	1,145/105,341	1.1%	0.83 (0.75, 0.91)
2–3 years follow–up	679/87,641	0.8%	928/98,712	0.9%	0.81 (0.73, 0.90)
3–4 years follow–up	555/74,249	0.7%	731/84,264	0.9%	0.84 (0.74, 0.95)
4–5 years follow–up	360/55,602	0.6%	450/64,356	0.7%	0.91 (0.79, 1.05)
>5 years follow–up	248/24,891	1.0%	276/27,997	1.0%	1.03 (0.86, 1.22)
					P for trend = 0.155
All follow–up	3,965/98,585	4.0%	5,058/110,374	4.6%	0.86 (0.83, 0.90)
**Heart failure**					
0–1 years follow–up	460/91,009	0.5%	737/102,898	0.7%	0.72 (0.62, 0.83)
1–2 years follow–up	447/87,141	0.5%	570/98,626	0.6%	0.92 (0.80, 1.06)
2–3 years follow–up	412/81,326	0.5%	548/92,605	0.6%	0.92 (0.80, 1.06)
3–4 years follow–up	398/68,515	0.6%	512/78,718	0.7%	0.90 (0.77, 1.05)
4–5 years follow–up	229/51,149	0.4%	312/60,126	0.5%	0.93 (0.75, 1.15)
>5 years follow–up	121/21,815	0.6%	164/25,037	0.7%	0.93 (0.73, 1.19)
					P for trend = 0.021
All follow–up	2,067/91,009	2.3%	2,843/102,898	2.8%	0.87 (0.81, 0.93)
**Cardiovascular death**					
0–1 years follow–up	672/97,925	0.7%	808/109,888	0.7%	0.90 (0.81, 1.01)
1–2 years follow–up	698/94,322	0.7%	865/106,127	0.8%	0.88 (0.79, 0.99)
2–3 years follow–up	698/88,610	0.8%	866/100,322	0.9%	0.94 (0.84, 1.05)
3–4 years follow–up	632/75,477	0.8%	802/86,226	0.9%	0.92 (0.82, 1.02)
4–5 years follow–up	432/56,629	0.8%	577/66,178	0.9%	0.90 (0.78, 1.03)
>5 years follow–up	303/25,292	1.2%	400/28,856	1.4%	0.89 (0.77, 1.04)
					P for trend = 0.710
All follow–up	3,435/97,925	3.5%	4,318/109,888	3.9%	0.91 (0.86, 0.95)
**All–cause death**					
0–1 years follow–up	1,467/98,509	1.5%	1,773/110,287	1.6%	0.93 (0.86, 1.00)
1–2 years follow–up	1,812/94,909	1.9%	2,138/106,533	2.0%	0.95 (0.89, 1.02)
2–3 years follow–up	1,841/89,128	2.1%	2,326/100,636	2.3%	0.92 (0.86, 0.99)
3–4 years follow–up	1,747/75,867	2.3%	2,133/86,425	2.5%	0.96 (0.89, 1.02)
4–5 years follow–up	1,212/57,008	2.1%	1,454/66,377	2.2%	1.01 (0.92, 1.10)
>5 years follow–up	825/25,661	3.2%	931/29,053	3.2%	1.00 (0.91, 1.10)
					P for trend = 0.101
All follow–up	8,904/98,509	9.0%	10,755/110,287	9.8%	0.96 (0.93, 0.99)

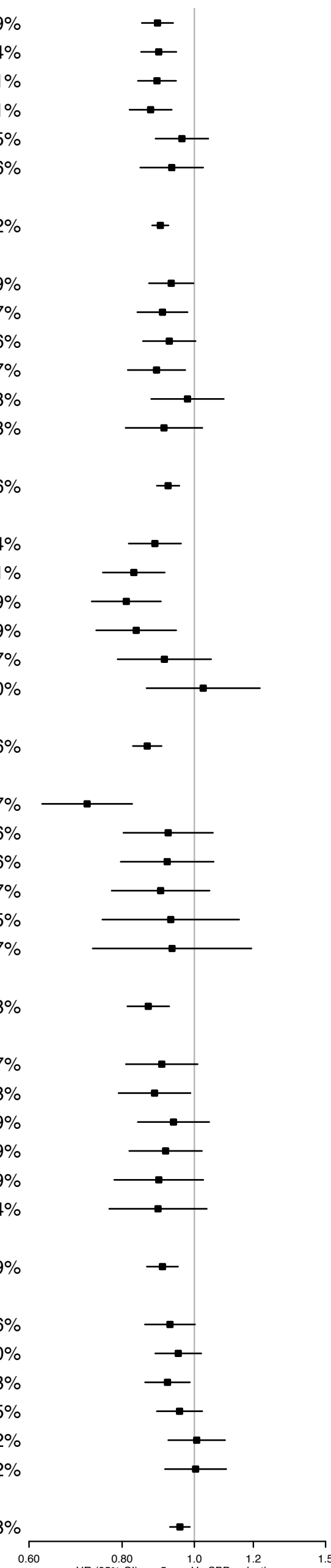

Supplementary Fig. 12. Standardised effects of systolic blood pressure treatment on outcomes among women, stratified by follow-up duration

Follow–up period	Treatment group (n/N)	Incidence (%)	Control group (n/N)	Incidence (%)	HR (95% CI) per 5 mm Hg SBP reduction
**Major cardiovascular events**					
0–1 years follow–up	1,756/66,205	2.7%	2,386/73,725	3.2%	0.85 (0.79, 0.90)
1–2 years follow–up	1,490/62,084	2.4%	1,974/68,817	2.9%	0.86 (0.81, 0.93)
2–3 years follow–up	1,321/57,029	2.3%	1,514/63,364	2.4%	1.03 (0.96, 1.12)
3–4 years follow–up	985/47,598	2.1%	1,327/52,805	2.5%	0.87 (0.79, 0.95)
4–5 years follow–up	736/34,434	2.1%	884/38,617	2.3%	0.98 (0.88, 1.08)
>5 years follow–up	466/16,399	2.8%	546/18,033	3.0%	0.94 (0.83, 1.07)
					P for trend = 0.014
All follow–up	6,754/66,205	10.2%	8,631/73,725	11.7%	0.90 (0.87, 0.93)
**Ischaemic heart disease**					
0–1 years follow–up	701/66,193	1.1%	959/73,735	1.3%	0.91 (0.82, 1.01)
1–2 years follow–up	631/62,792	1.0%	873/69,855	1.2%	0.89 (0.80, 0.99)
2–3 years follow–up	563/58,198	1.0%	673/65,020	1.0%	1.09 (0.96, 1.23)
3–4 years follow–up	433/48,980	0.9%	604/54,691	1.1%	0.87 (0.75, 0.99)
4–5 years follow–up	326/35,717	0.9%	417/40,462	1.0%	0.99 (0.84, 1.15)
>5 years follow–up	253/17,062	1.5%	313/19,019	1.6%	0.90 (0.76, 1.07)
					P for trend = 0.681
All follow–up	2,907/66,193	4.4%	3,839/73,735	5.2%	0.93 (0.89, 0.98)
**Stroke**					
0–1 years follow–up	703/66,208	1.1%	919/73,720	1.2%	0.84 (0.76, 0.93)
1–2 years follow–up	578/62,768	0.9%	739/69,857	1.1%	0.86 (0.77, 0.97)
2–3 years follow–up	471/58,227	0.8%	565/65,137	0.9%	0.93 (0.81, 1.05)
3–4 years follow–up	344/49,175	0.7%	480/54,971	0.9%	0.82 (0.71, 0.95)
4–5 years follow–up	288/36,071	0.8%	367/40,904	0.9%	0.90 (0.77, 1.05)
>5 years follow–up	177/17,408	1.0%	218/19,397	1.1%	0.95 (0.78, 1.17)
					P for trend = 0.416
All follow–up	2,561/66,208	3.9%	3,288/73,720	4.5%	0.87 (0.82, 0.92)
**Heart failure**					
0–1 years follow–up	304/58,785	0.5%	502/66,584	0.8%	0.72 (0.62, 0.84)
1–2 years follow–up	284/55,715	0.5%	363/63,110	0.6%	0.91 (0.76, 1.08)
2–3 years follow–up	270/51,526	0.5%	352/58,759	0.6%	0.93 (0.78, 1.10)
3–4 years follow–up	230/42,985	0.5%	308/49,099	0.6%	0.91 (0.75, 1.11)
4–5 years follow–up	151/31,253	0.5%	216/36,446	0.6%	0.86 (0.69, 1.08)
>5 years follow–up	97/14,204	0.7%	99/16,478	0.6%	1.15 (0.86, 1.53)
					P for trend = 0.007
All follow–up	1,336/58,785	2.3%	1,840/66,584	2.8%	0.87 (0.81, 0.94)
**Cardiovascular death**					
0–1 years follow–up	392/65,442	0.6%	478/73,142	0.7%	0.93 (0.81, 1.07)
1–2 years follow–up	418/62,559	0.7%	545/70,011	0.8%	0.90 (0.79, 1.03)
2–3 years follow–up	392/58,516	0.7%	470/65,894	0.7%	1.02 (0.89, 1.18)
3–4 years follow–up	360/49,771	0.7%	450/56,038	0.8%	0.96 (0.83, 1.12)
4–5 years follow–up	262/36,581	0.7%	326/41,906	0.8%	0.98 (0.83, 1.16)
>5 years follow–up	223/17,618	1.3%	242/19,978	1.2%	1.15 (0.95, 1.38)
					P for trend = 0.065
All follow–up	2,047/65,442	3.1%	2,511/73,142	3.4%	0.97 (0.91, 1.03)
**All–cause death**					
0–1 years follow–up	764/66,046	1.2%	941/73,532	1.3%	0.92 (0.83, 1.02)
1–2 years follow–up	1,000/63,175	1.6%	1,190/70,425	1.7%	0.99 (0.91, 1.09)
2–3 years follow–up	1,013/59,018	1.7%	1,189/66,181	1.8%	1.02 (0.93, 1.12)
3–4 years follow–up	897/50,100	1.8%	1,101/56,184	2.0%	0.97 (0.88, 1.07)
4–5 years follow–up	632/36,905	1.7%	821/42,053	2.0%	0.95 (0.85, 1.06)
>5 years follow–up	504/17,938	2.8%	553/20,123	2.7%	1.08 (0.95, 1.22)
					P for trend = 0.280
All follow–up	4,810/66,046	7.3%	5,795/73,532	7.9%	0.98 (0.94, 1.02)

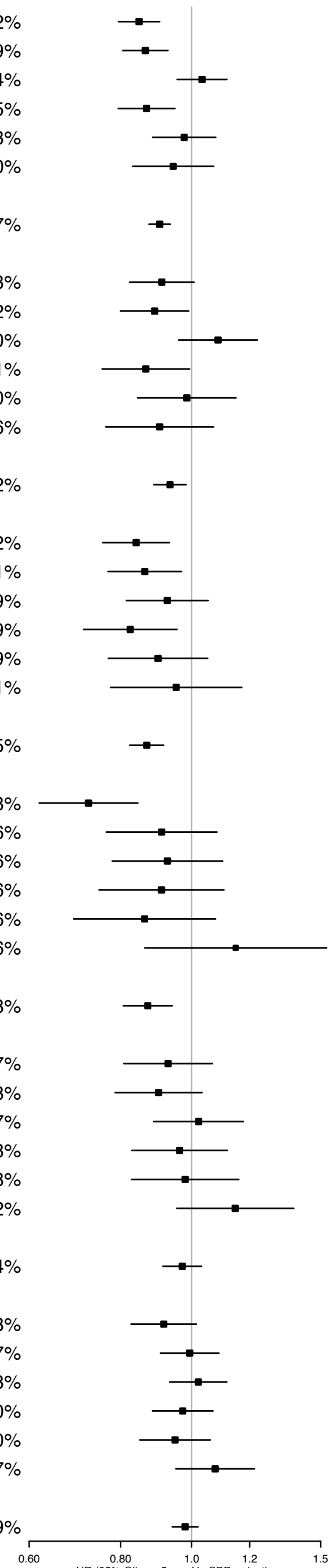

Supplementary Fig. 13. Standardised effects of systolic blood pressure treatment on outcomes among participants with diabetes, stratified by follow-up duration

Follow−up period	Treatment group (n/N)	Incidence (%)	Control group (n/N)	Incidence (%)	HR (95% CI) per 5 mm Hg SBP reduction
**Major cardiovascular events**					
0−1 years follow−up	2,122/46,772	4.5%	2,589/53,075	4.9%	0.94 (0.88, 1.00)
1−2 years follow−up	1,742/43,391	4.0%	2,169/49,173	4.4%	0.91 (0.85, 0.98)
2−3 years follow−up	1,484/39,537	3.8%	1,816/45,077	4.0%	0.97 (0.89, 1.04)
3−4 years follow−up	1,233/33,577	3.7%	1,564/38,211	4.1%	0.90 (0.83, 0.97)
4−5 years follow−up	756/25,443	3.0%	873/29,236	3.0%	1.02 (0.92, 1.13)
>5 years follow−up	379/9,973	3.8%	476/11,315	4.2%	0.90 (0.78, 1.03)
					P for trend = 0.665
All follow−up	7,716/46,772	16.5%	9,487/53,075	17.9%	0.93 (0.90, 0.97)
**Ischaemic heart disease**					
0−1 years follow−up	948/46,744	2.0%	1,130/53,075	2.1%	1.03 (0.94, 1.13)
1−2 years follow−up	771/44,202	1.7%	1,054/50,302	2.1%	0.90 (0.81, 1.00)
2−3 years follow−up	716/40,908	1.8%	889/46,869	1.9%	1.01 (0.90, 1.13)
3−4 years follow−up	574/35,192	1.6%	796/40,334	2.0%	0.87 (0.77, 0.97)
4−5 years follow−up	430/27,067	1.6%	503/31,428	1.6%	1.07 (0.93, 1.22)
>5 years follow−up	262/10,546	2.5%	327/12,161	2.7%	0.91 (0.77, 1.07)
					P for trend = 0.475
All follow−up	3,701/46,744	7.9%	4,699/53,075	8.9%	0.96 (0.92, 1.01)
**Stroke**					
0−1 years follow−up	699/46,756	1.5%	859/53,052	1.6%	0.86 (0.76, 0.97)
1−2 years follow−up	535/44,312	1.2%	659/50,428	1.3%	0.87 (0.76, 0.99)
2−3 years follow−up	412/41,145	1.0%	524/47,269	1.1%	0.88 (0.76, 1.02)
3−4 years follow−up	346/35,709	1.0%	451/41,041	1.1%	0.84 (0.72, 0.99)
4−5 years follow−up	179/27,726	0.6%	250/32,340	0.8%	0.83 (0.67, 1.02)
>5 years follow−up	111/11,185	1.0%	152/12,897	1.2%	0.84 (0.65, 1.08)
					P for trend = 0.736
All follow−up	2,282/46,756	4.9%	2,895/53,052	5.5%	0.85 (0.80, 0.91)
**Heart failure**					
0−1 years follow−up	414/44,969	0.9%	612/51,591	1.2%	0.83 (0.70, 0.98)
1−2 years follow−up	387/42,798	0.9%	481/49,174	1.0%	0.96 (0.81, 1.14)
2−3 years follow−up	353/39,786	0.9%	478/46,168	1.0%	0.92 (0.79, 1.08)
3−4 years follow−up	332/34,391	1.0%	425/39,937	1.1%	0.93 (0.79, 1.09)
4−5 years follow−up	197/26,564	0.7%	248/31,347	0.8%	0.96 (0.77, 1.19)
>5 years follow−up	99/10,173	1.0%	130/12,144	1.1%	1.02 (0.78, 1.35)
					P for trend = 0.035
All follow−up	1,782/44,969	4.0%	2,374/51,591	4.6%	0.92 (0.85, 0.99)
**Cardiovascular death**					
0−1 years follow−up	475/45,955	1.0%	478/52,634	0.9%	1.08 (0.93, 1.25)
1−2 years follow−up	475/44,057	1.1%	545/50,701	1.1%	0.93 (0.79, 1.08)
2−3 years follow−up	432/41,225	1.0%	530/47,941	1.1%	0.95 (0.82, 1.11)
3−4 years follow−up	438/35,884	1.2%	517/41,852	1.2%	1.01 (0.87, 1.18)
4−5 years follow−up	253/27,883	0.9%	364/33,138	1.1%	0.82 (0.68, 0.98)
>5 years follow−up	185/10,888	1.7%	213/13,120	1.6%	1.13 (0.92, 1.38)
					P for trend = 0.330
All follow−up	2,258/45,955	4.9%	2,647/52,634	5.0%	0.98 (0.92, 1.05)
**All−cause death**					
0−1 years follow−up	873/46,697	1.9%	1,022/53,001	1.9%	0.95 (0.86, 1.07)
1−2 years follow−up	1,120/44,794	2.5%	1,272/51,064	2.5%	0.99 (0.89, 1.09)
2−3 years follow−up	1,118/41,963	2.7%	1,337/48,303	2.8%	0.98 (0.89, 1.08)
3−4 years follow−up	1,038/36,613	2.8%	1,250/42,215	3.0%	1.01 (0.91, 1.11)
4−5 years follow−up	691/28,590	2.4%	899/33,495	2.7%	0.94 (0.84, 1.05)
>5 years follow−up	468/11,577	4.0%	497/13,465	3.7%	1.07 (0.94, 1.22)
					P for trend = 0.736
All follow−up	5,308/46,697	11.4%	6,277/53,001	11.8%	0.99 (0.95, 1.03)

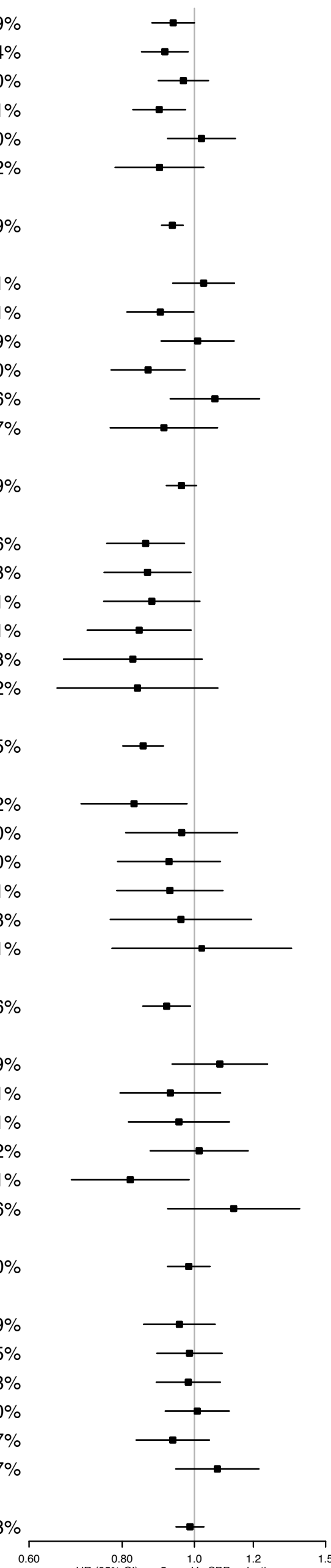

Supplementary Fig. 14. Standardised effects of systolic blood pressure treatment on outcomes among participants without diabetes, stratified by follow-up duration

Follow–up period	Treatment group (n/N)	Incidence (%)	Control group (n/N)	Incidence (%)	HR (95% CI) per 5 mm Hg SBP reduction
**Major cardiovascular events**					
0–1 years follow–up	3,014/114,621	2.6%	4,123/127,515	3.2%	0.84 (0.80, 0.88)
1–2 years follow–up	2,491/107,859	2.3%	3,292/119,534	2.8%	0.86 (0.82, 0.91)
2–3 years follow–up	2,158/99,324	2.2%	2,674/110,186	2.4%	0.92 (0.87, 0.98)
3–4 years follow–up	1,676/83,384	2.0%	2,257/92,861	2.4%	0.85 (0.79, 0.91)
4–5 years follow–up	1,235/60,894	2.0%	1,499/68,854	2.2%	0.94 (0.87, 1.02)
>5 years follow–up	842/29,281	2.9%	992/32,052	3.1%	0.96 (0.87, 1.05)
					P for trend = 0.001
All follow–up	11,416/114,621	10.0%	14,837/127,515	11.6%	0.88 (0.86, 0.90)
**Ischaemic heart disease**					
0–1 years follow–up	1,390/114,646	1.2%	1,924/127,546	1.5%	0.87 (0.81, 0.93)
1–2 years follow–up	1,204/109,037	1.1%	1,632/121,154	1.3%	0.90 (0.83, 0.97)
2–3 years follow–up	1,090/101,156	1.1%	1,381/112,692	1.2%	0.95 (0.87, 1.03)
3–4 years follow–up	882/85,439	1.0%	1,181/95,651	1.2%	0.89 (0.80, 0.98)
4–5 years follow–up	599/62,683	1.0%	750/71,445	1.0%	0.92 (0.81, 1.04)
>5 years follow–up	493/30,358	1.6%	613/33,519	1.8%	0.91 (0.80, 1.02)
					P for trend = 0.221
All follow–up	5,658/114,646	4.9%	7,481/127,546	5.9%	0.90 (0.87, 0.94)
**Stroke**					
0–1 years follow–up	1,231/114,636	1.1%	1,587/127,517	1.2%	0.86 (0.79, 0.93)
1–2 years follow–up	937/109,100	0.9%	1,225/121,322	1.0%	0.83 (0.76, 0.91)
2–3 years follow–up	738/101,474	0.7%	969/113,211	0.9%	0.84 (0.76, 0.93)
3–4 years follow–up	553/86,156	0.6%	760/96,604	0.8%	0.82 (0.73, 0.93)
4–5 years follow–up	469/63,792	0.7%	566/72,785	0.8%	0.95 (0.84, 1.07)
>5 years follow–up	314/31,082	1.0%	342/34,471	1.0%	1.05 (0.90, 1.23)
					P for trend = 0.025
All follow–up	4,242/114,636	3.7%	5,449/127,517	4.3%	0.86 (0.83, 0.90)
**Heart failure**					
0–1 years follow–up	350/101,442	0.3%	627/114,368	0.5%	0.66 (0.57, 0.76)
1–2 years follow–up	344/96,748	0.4%	451/109,112	0.4%	0.90 (0.78, 1.04)
2–3 years follow–up	329/89,832	0.4%	422/101,825	0.4%	0.93 (0.79, 1.08)
3–4 years follow–up	296/75,563	0.4%	395/86,296	0.5%	0.87 (0.73, 1.04)
4–5 years follow–up	183/55,688	0.3%	280/65,092	0.4%	0.83 (0.67, 1.02)
>5 years follow–up	118/25,816	0.5%	133/29,346	0.5%	1.06 (0.82, 1.36)
					P for trend = 0.004
All follow–up	1,620/101,442	1.6%	2,308/114,368	2.0%	0.83 (0.78, 0.89)
**Cardiovascular death**					
0–1 years follow–up	586/113,964	0.5%	805/126,828	0.6%	0.82 (0.73, 0.91)
1–2 years follow–up	636/109,450	0.6%	864/121,946	0.7%	0.84 (0.76, 0.94)
2–3 years follow–up	658/102,607	0.6%	806/114,863	0.7%	0.97 (0.88, 1.08)
3–4 years follow–up	554/87,775	0.6%	734/98,795	0.7%	0.89 (0.79, 1.00)
4–5 years follow–up	441/65,169	0.7%	539/74,808	0.7%	0.98 (0.86, 1.11)
>5 years follow–up	340/31,990	1.1%	429/35,687	1.2%	0.94 (0.82, 1.09)
					P for trend = 0.038
All follow–up	3,215/113,964	2.8%	4,177/126,828	3.3%	0.90 (0.85, 0.94)
**All–cause death**					
0–1 years follow–up	1,354/114,410	1.2%	1,688/127,250	1.3%	0.90 (0.84, 0.97)
1–2 years follow–up	1,686/109,916	1.5%	2,053/122,403	1.7%	0.95 (0.89, 1.02)
2–3 years follow–up	1,735/102,889	1.7%	2,178/115,102	1.9%	0.94 (0.88, 1.01)
3–4 years follow–up	1,606/87,765	1.8%	1,983/98,777	2.0%	0.94 (0.88, 1.01)
4–5 years follow–up	1,152/65,165	1.8%	1,376/74,797	1.8%	1.02 (0.93, 1.11)
>5 years follow–up	860/31,990	2.7%	987/35,684	2.8%	1.01 (0.92, 1.11)
					P for trend = 0.016
All follow–up	8,393/114,410	7.3%	10,265/127,250	8.1%	0.95 (0.92, 0.98)

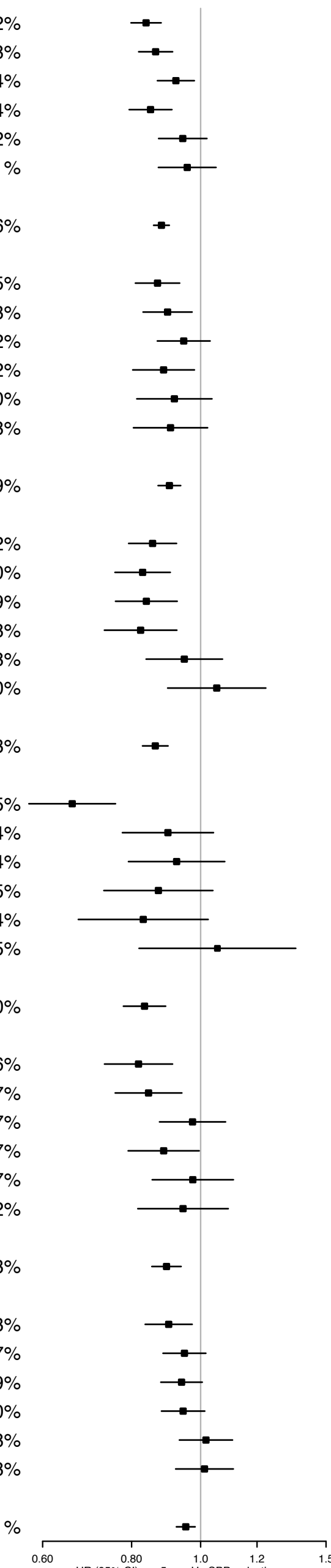

Supplementary Fig. 15. Effects of antihypertensive drug classes on ischaemic heart disease, stratified by follow-up duration

Follow–up period	Included trials	HR (95% CI)
**ACEI vs placebo**		
0–1 years follow–up	31	0.99 (0.87, 1.12)
1–2 years follow–up	30	0.84 (0.74, 0.97)
2–3 years follow–up	30	0.82 (0.71, 0.96)
3–4 years follow–up	25	0.67 (0.57, 0.78)
4–5 years follow–up	23	0.83 (0.66, 1.04)
>5 years follow–up	17	0.77 (0.49, 1.19)
		P for trend = 0.098
All follow–up	34	0.83 (0.78, 0.89)
**ARB vs placebo**		
0–1 years follow–up	31	1.06 (0.90, 1.24)
1–2 years follow–up	30	0.93 (0.78, 1.11)
2–3 years follow–up	30	1.02 (0.84, 1.25)
3–4 years follow–up	25	0.74 (0.59, 0.92)
4–5 years follow–up	23	0.94 (0.70, 1.26)
>5 years follow–up	17	0.76 (0.40, 1.45)
		P for trend = 0.152
All follow–up	34	0.94 (0.87, 1.03)
**Beta blocker vs placebo**		
0–1 years follow–up	31	0.87 (0.71, 1.06)
1–2 years follow–up	30	0.97 (0.78, 1.20)
2–3 years follow–up	30	1.11 (0.88, 1.41)
3–4 years follow–up	25	0.69 (0.53, 0.90)
4–5 years follow–up	23	0.94 (0.67, 1.31)
>5 years follow–up	17	0.94 (0.55, 1.61)
		P for trend = 0.831
All follow–up	34	0.92 (0.83, 1.01)
**Calcium channel blocker vs placebo**		
0–1 years follow–up	31	0.86 (0.74, 1.00)
1–2 years follow–up	30	0.79 (0.67, 0.93)
2–3 years follow–up	30	0.95 (0.79, 1.13)
3–4 years follow–up	25	0.70 (0.58, 0.86)
4–5 years follow–up	23	0.92 (0.70, 1.20)
>5 years follow–up	17	0.74 (0.46, 1.17)
		P for trend = 0.735
All follow–up	34	0.83 (0.77, 0.90)
**Thiazide diuretics vs placebo**		
0–1 years follow–up	31	0.86 (0.73, 1.03)
1–2 years follow–up	30	0.79 (0.67, 0.95)
2–3 years follow–up	30	0.90 (0.74, 1.09)
3–4 years follow–up	25	0.67 (0.55, 0.82)
4–5 years follow–up	23	0.97 (0.73, 1.28)
>5 years follow–up	17	0.66 (0.41, 1.06)
		P for trend = 0.654
All follow–up	34	0.81 (0.75, 0.88)

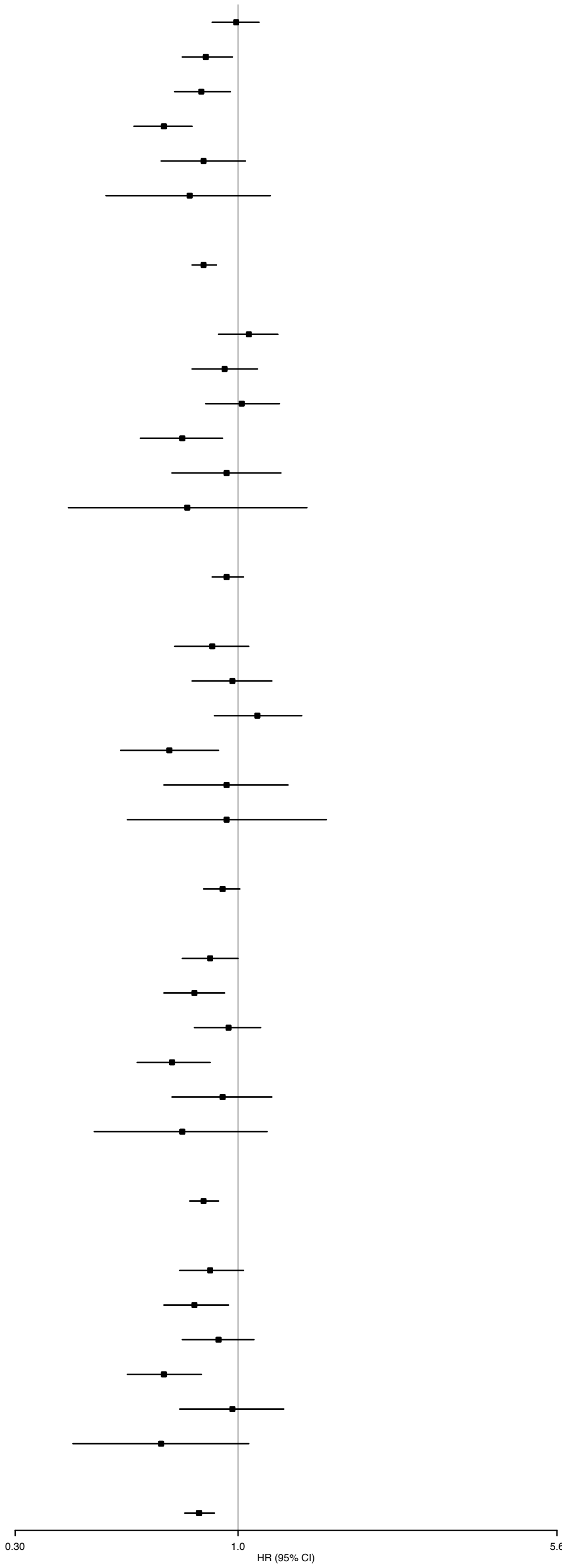

Supplementary Fig. 16. Effects of antihypertensive drug classes on stroke, stratified by follow-up duration

Follow–up period	Included trials	HR (95% CI)
**ACEI vs placebo**		
0–1 years follow–up	32	0.96 (0.82, 1.13)
1–2 years follow–up	31	0.82 (0.69, 0.98)
2–3 years follow–up	29	0.86 (0.71, 1.05)
3–4 years follow–up	28	0.79 (0.64, 0.98)
4–5 years follow–up	21	0.89 (0.66, 1.20)
>5 years follow–up	14	1.06 (0.61, 1.85)
		P for trend = 0.486
All follow–up	34	0.88 (0.81, 0.96)
**ARB vs placebo**		
0–1 years follow–up	32	0.92 (0.84, 1.02)
1–2 years follow–up	31	0.93 (0.81, 1.07)
2–3 years follow–up	29	0.85 (0.71, 1.00)
3–4 years follow–up	28	0.71 (0.57, 0.89)
4–5 years follow–up	21	0.82 (0.60, 1.12)
>5 years follow–up	14	0.68 (0.32, 1.46)
		P for trend = 0.046
All follow–up	34	0.88 (0.82, 0.94)
**Beta blocker vs placebo**		
0–1 years follow–up	32	0.97 (0.78, 1.20)
1–2 years follow–up	31	1.01 (0.79, 1.29)
2–3 years follow–up	29	1.07 (0.81, 1.40)
3–4 years follow–up	28	0.91 (0.66, 1.27)
4–5 years follow–up	21	1.11 (0.74, 1.68)
>5 years follow–up	14	0.90 (0.45, 1.80)
		P for trend = 0.795
All follow–up	34	1.02 (0.91, 1.14)
**Calcium channel blocker vs placebo**		
0–1 years follow–up	32	0.69 (0.58, 0.81)
1–2 years follow–up	31	0.79 (0.65, 0.96)
2–3 years follow–up	29	0.89 (0.71, 1.11)
3–4 years follow–up	28	0.67 (0.52, 0.87)
4–5 years follow–up	21	0.94 (0.66, 1.33)
>5 years follow–up	14	0.80 (0.44, 1.48)
		P for trend = 0.375
All follow–up	34	0.78 (0.71, 0.85)
**Thiazide diuretics vs placebo**		
0–1 years follow–up	32	0.78 (0.62, 0.97)
1–2 years follow–up	31	0.70 (0.54, 0.90)
2–3 years follow–up	29	0.75 (0.57, 0.99)
3–4 years follow–up	28	0.75 (0.55, 1.02)
4–5 years follow–up	21	0.85 (0.56, 1.28)
>5 years follow–up	14	0.97 (0.51, 1.81)
		P for trend = 0.343
All follow–up	34	0.77 (0.69, 0.87)

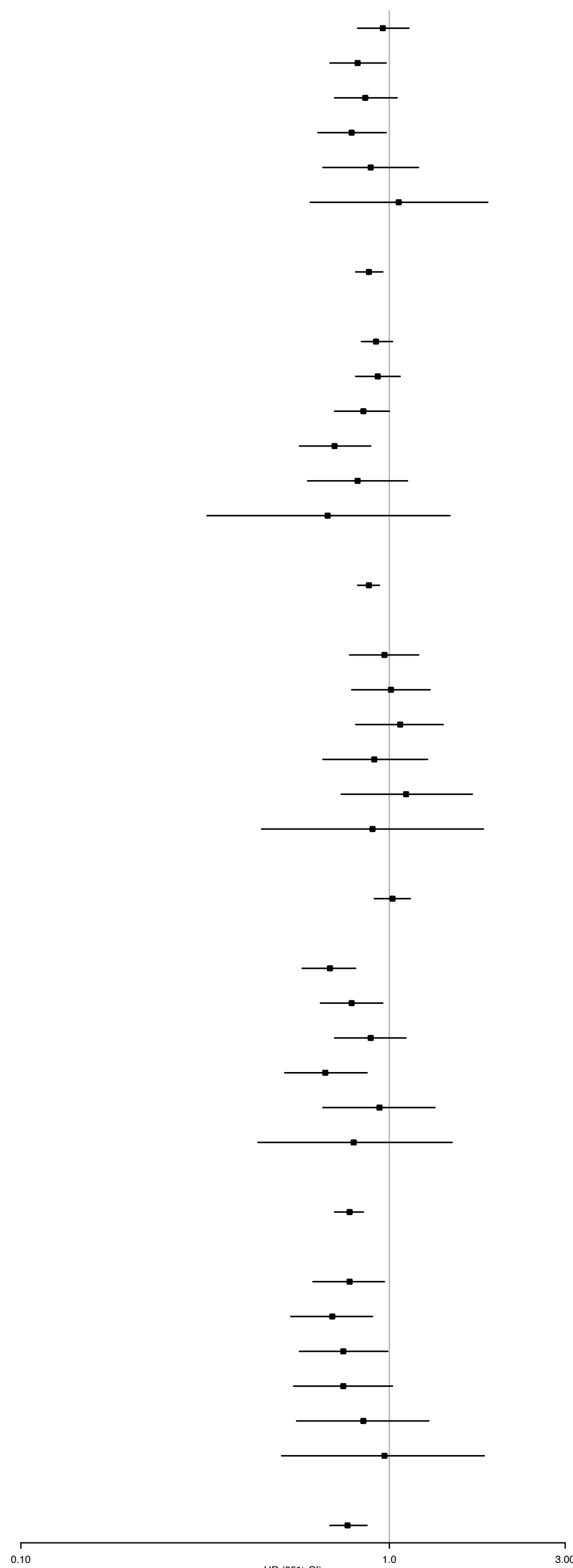

Supplementary Fig. 17. Effects of antihypertensive drug classes on heart failure, stratified by follow-up duration

Follow–up period	Included trials	HR (95% CI)
**ACEI vs placebo**		
0–1 years follow–up	22	0.73 (0.59, 0.91)
1–2 years follow–up	24	0.77 (0.61, 0.97)
2–3 years follow–up	22	0.88 (0.69, 1.11)
3–4 years follow–up	21	0.91 (0.71, 1.18)
4–5 years follow–up	18	0.79 (0.56, 1.10)
>5 years follow–up	16	0.99 (0.56, 1.74)
		P for trend = 0.079
All follow–up	29	0.79 (0.71, 0.88)
**ARB vs placebo**		
0–1 years follow–up	22	0.92 (0.78, 1.10)
1–2 years follow–up	24	0.93 (0.77, 1.14)
2–3 years follow–up	22	0.93 (0.75, 1.15)
3–4 years follow–up	21	0.89 (0.69, 1.14)
4–5 years follow–up	18	1.00 (0.71, 1.41)
>5 years follow–up	16	1.08 (0.54, 2.14)
		P for trend = 0.402
All follow–up	29	0.91 (0.83, 1.01)
**Beta blocker vs placebo**		
0–1 years follow–up	22	1.14 (0.71, 1.85)
1–2 years follow–up	24	0.73 (0.41, 1.29)
2–3 years follow–up	22	1.17 (0.68, 2.02)
3–4 years follow–up	21	1.30 (0.75, 2.26)
4–5 years follow–up	18	1.49 (0.81, 2.72)
>5 years follow–up	16	1.34 (0.59, 3.07)
		P for trend = 0.205
All follow–up	29	1.12 (0.90, 1.40)
**Calcium channel blocker vs placebo**		
0–1 years follow–up	22	0.85 (0.68, 1.07)
1–2 years follow–up	24	0.85 (0.65, 1.10)
2–3 years follow–up	22	1.04 (0.79, 1.37)
3–4 years follow–up	21	1.23 (0.91, 1.65)
4–5 years follow–up	18	1.25 (0.84, 1.84)
>5 years follow–up	16	1.15 (0.61, 2.15)
		P for trend = 0.010
All follow–up	29	0.98 (0.87, 1.10)
**Thiazide diuretics vs placebo**		
0–1 years follow–up	22	0.40 (0.30, 0.54)
1–2 years follow–up	24	0.76 (0.55, 1.04)
2–3 years follow–up	22	0.81 (0.58, 1.13)
3–4 years follow–up	21	0.92 (0.64, 1.31)
4–5 years follow–up	18	1.01 (0.64, 1.58)
>5 years follow–up	16	0.95 (0.52, 1.72)
		P for trend = 0.034
All follow–up	29	0.71 (0.61, 0.82)

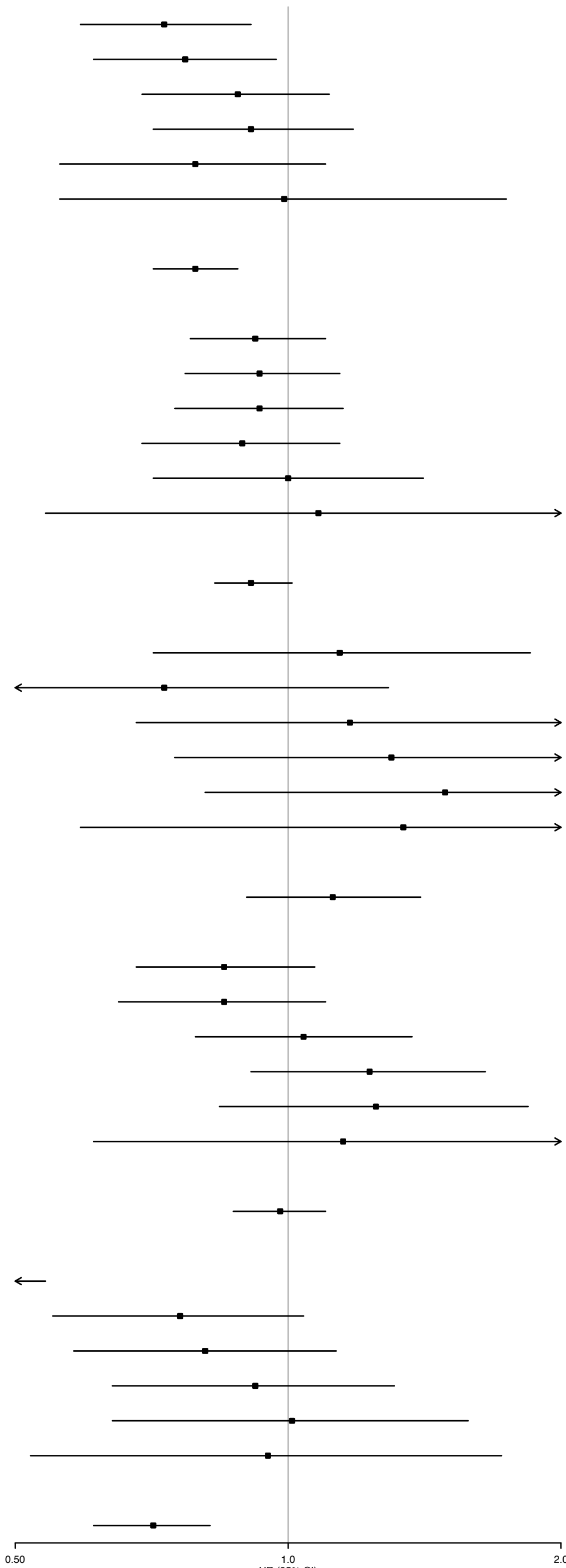

Supplementary Fig. 18. Effects of antihypertensive drug classes on cardiovascular death, stratified by follow-up duration

Follow−up period	Included trials	HR (95% CI)
**ACEI vs placebo**		
0−1 years follow−up	29	0.97 (0.80, 1.18)
1−2 years follow−up	30	0.85 (0.70, 1.02)
2−3 years follow−up	27	0.94 (0.78, 1.12)
3−4 years follow−up	24	0.86 (0.71, 1.03)
4−5 years follow−up	22	1.00 (0.78, 1.28)
>5 years follow−up	13	0.95 (0.61, 1.49)
		P for trend = 0.916
All follow−up	32	0.91 (0.83, 0.98)
**ARB vs placebo**		
0−1 years follow−up	29	1.08 (0.90, 1.29)
1−2 years follow−up	30	1.15 (0.96, 1.37)
2−3 years follow−up	27	0.99 (0.81, 1.21)
3−4 years follow−up	24	0.82 (0.66, 1.03)
4−5 years follow−up	22	1.24 (0.95, 1.62)
>5 years follow−up	13	0.99 (0.54, 1.84)
		P for trend = 0.735
All follow−up	32	1.03 (0.95, 1.13)
**Beta blocker vs placebo**		
0−1 years follow−up	29	0.91 (0.68, 1.21)
1−2 years follow−up	30	1.16 (0.87, 1.55)
2−3 years follow−up	27	1.16 (0.86, 1.56)
3−4 years follow−up	24	0.89 (0.63, 1.27)
4−5 years follow−up	22	1.54 (1.02, 2.32)
>5 years follow−up	13	1.53 (0.82, 2.87)
		P for trend = 0.195
All follow−up	32	1.10 (0.96, 1.25)
**Calcium channel blocker vs placebo**		
0−1 years follow−up	29	0.91 (0.73, 1.15)
1−2 years follow−up	30	0.92 (0.73, 1.15)
2−3 years follow−up	27	1.01 (0.81, 1.27)
3−4 years follow−up	24	0.84 (0.66, 1.07)
4−5 years follow−up	22	0.94 (0.70, 1.26)
>5 years follow−up	13	0.94 (0.58, 1.51)
		P for trend = 0.937
All follow−up	32	0.92 (0.83, 1.01)
**Thiazide diuretics vs placebo**		
0−1 years follow−up	29	0.83 (0.63, 1.09)
1−2 years follow−up	30	0.72 (0.55, 0.93)
2−3 years follow−up	27	0.98 (0.76, 1.27)
3−4 years follow−up	24	0.83 (0.64, 1.07)
4−5 years follow−up	22	0.99 (0.72, 1.37)
>5 years follow−up	13	0.96 (0.59, 1.56)
		P for trend = 0.245
All follow−up	32	0.86 (0.77, 0.96)

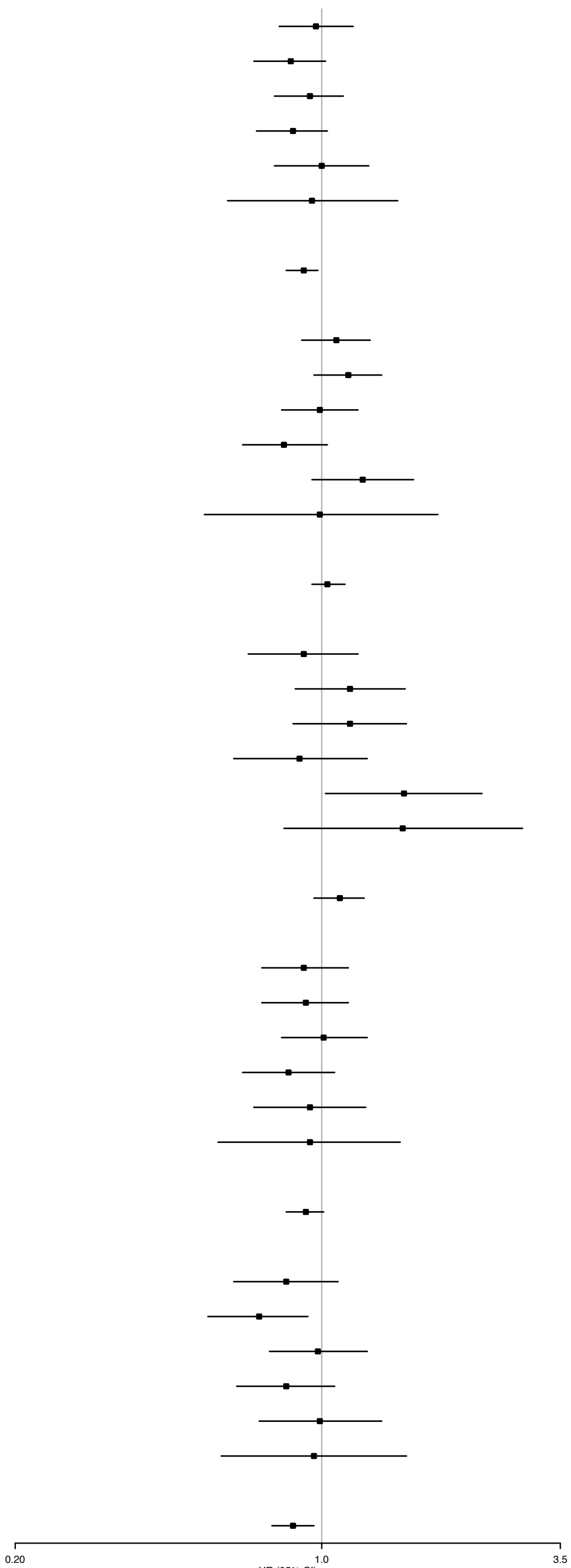

Supplementary Fig. 19. Effects of antihypertensive drug classes on all-cause death, stratified by follow-up duration

Follow–up period	Included trials	HR (95% CI)
**ACEI vs placebo**		
0–1 years follow–up	32	0.90 (0.79, 1.02)
1–2 years follow–up	32	0.99 (0.89, 1.11)
2–3 years follow–up	30	0.94 (0.84, 1.05)
3–4 years follow–up	26	0.87 (0.78, 0.98)
4–5 years follow–up	23	1.11 (0.96, 1.29)
>5 years follow–up	15	0.98 (0.76, 1.28)
		P for trend = 0.452
All follow–up	33	0.95 (0.90, 1.00)
**ARB vs placebo**		
0–1 years follow–up	32	1.03 (0.92, 1.15)
1–2 years follow–up	32	1.07 (0.96, 1.19)
2–3 years follow–up	30	0.91 (0.81, 1.02)
3–4 years follow–up	26	0.85 (0.75, 0.97)
4–5 years follow–up	23	1.14 (0.97, 1.35)
>5 years follow–up	15	0.96 (0.68, 1.36)
		P for trend = 0.793
All follow–up	33	0.98 (0.93, 1.04)
**Beta blocker vs placebo**		
0–1 years follow–up	32	0.87 (0.72, 1.06)
1–2 years follow–up	32	1.12 (0.93, 1.34)
2–3 years follow–up	30	1.09 (0.91, 1.31)
3–4 years follow–up	26	0.95 (0.77, 1.18)
4–5 years follow–up	23	1.36 (1.07, 1.73)
>5 years follow–up	15	1.05 (0.73, 1.50)
		P for trend = 0.271
All follow–up	33	1.06 (0.97, 1.15)
**Calcium channel blocker vs placebo**		
0–1 years follow–up	32	0.80 (0.69, 0.93)
1–2 years follow–up	32	1.00 (0.87, 1.14)
2–3 years follow–up	30	0.92 (0.80, 1.06)
3–4 years follow–up	26	0.90 (0.78, 1.04)
4–5 years follow–up	23	1.17 (0.97, 1.40)
>5 years follow–up	15	0.99 (0.74, 1.31)
		P for trend = 0.172
All follow–up	33	0.94 (0.89, 1.01)
**Thiazide diuretics vs placebo**		
0–1 years follow–up	32	0.84 (0.69, 1.03)
1–2 years follow–up	32	0.89 (0.75, 1.06)
2–3 years follow–up	30	0.96 (0.81, 1.13)
3–4 years follow–up	26	0.94 (0.79, 1.11)
4–5 years follow–up	23	1.11 (0.90, 1.36)
>5 years follow–up	15	1.06 (0.79, 1.41)
		P for trend = 0.012
All follow–up	33	0.95 (0.88, 1.02)

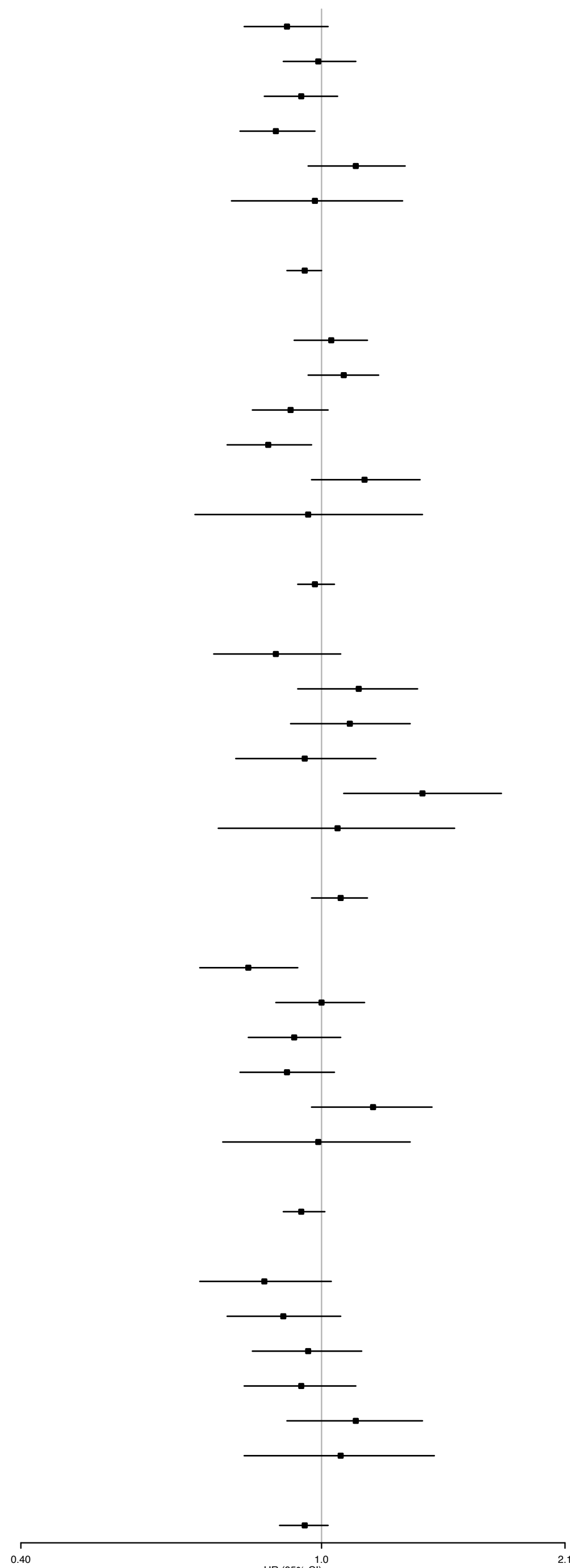

Supplementary Table 1. PRISMA-IPD Checklist of items to include when reporting a systematic review and meta-analysis of individual participant data (IPD)

PRISMA-IPD Section/topic	Item No	Checklist item	Reported on page
**Title**			
Title	1	Identify the report as a systematic review and meta-analysis of individual participant data.	1
**Abstract**			
Structured summary	2	Provide a structured summary including as applicable:	3-4
		**Background**: state research question and main objectives, with information on participants, interventions, comparators and outcomes.	
		**Methods**: report eligibility criteria; data sources including dates of last bibliographic search or elicitation, noting that IPD were sought; methods of assessing risk of bias.	
		**Results**: provide number and type of studies and participants identified and number (%) obtained; summary effect estimates for main outcomes (benefits and harms) with confidence intervals and measures of statistical heterogeneity. Describe the direction and size of summary effects in terms meaningful to those who would put findings into practice.	
		**Discussion:** state main strengths and limitations of the evidence, general interpretation of the results and any important implications.	
		**Other:** report primary funding source, registration number and registry name for the systematic review and IPD meta-analysis.	
**Introduction**			
Rationale	3	Describe the rationale for the review in the context of what is already known.	5
Objectives	4	Provide an explicit statement of the questions being addressed with reference, as applicable, to participants, interventions, comparisons, outcomes and study design (PICOS). Include any hypotheses that relate to particular types of participant-level subgroups.	5-7

**Methods**			
Protocol and registration	5	Indicate if a protocol exists and where it can be accessed. If available, provide registration information including registration number and registry name. Provide publication details, if applicable.	41
Eligibility criteria	6	Specify inclusion and exclusion criteria including those relating to participants, interventions, comparisons, outcomes, study design and characteristics (e.g. years when conducted, required minimum follow-up). Note whether these were applied at the study or individual level i.e. whether eligible participants were included (and ineligible participants excluded) from a study that included a wider population than specified by the review inclusion criteria. The rationale for criteria should be stated.	42
Identifying studies - information sources	7	Describe all methods of identifying published and unpublished studies including, as applicable: which bibliographic databases were searched with dates of coverage; details of any hand searching including of conference proceedings; use of study registers and agency or company databases; contact with the original research team and experts in the field; open adverts and surveys. Give the date of last search or elicitation.	42, reference 2
Identifying studies - search	8	Present the full electronic search strategy for at least one database, including any limits used, such that it could be repeated.	42, reference 2
Study selection processes	9	State the process for determining which studies were eligible for inclusion.	42, reference 2
Data collection processes	10	Describe how IPD were requested, collected and managed, including any processes for querying and confirming data with investigators. If IPD were not sought from any eligible study, the reason for this should be stated (for each such study).	43, reference 2
		If applicable, describe how any studies for which IPD were not available were dealt with. This should include whether, how and what aggregate data were sought or extracted from study reports and publications (such as extracting data independently in duplicate) and any processes for obtaining and confirming these data with investigators.	

Data items	11	Describe how the information and variables to be collected were chosen. List and define all study level and participant level data that were sought, including baseline and follow-up information. If applicable, describe methods of standardising or translating variables within the IPD datasets to ensure common scales or measurements across studies.	reference 2
IPD integrity	A1	Describe what aspects of IPD were subject to data checking (such as sequence generation, data consistency and completeness, baseline imbalance) and how this was done.	reference 2
Risk of bias assessment in individual studies.	12	Describe methods used to assess risk of bias in the individual studies and whether this was applied separately for each outcome. If applicable, describe how findings of IPD checking were used to inform the assessment. Report if and how risk of bias assessment was used in any data synthesis.	reference 2
Specification of outcomes and effect measures	13	State all treatment comparisons of interests. State all outcomes addressed and define them in detail. State whether they were pre-specified for the review and, if applicable, whether they were primary/main or secondary/additional outcomes. Give the principal measures of effect (such as risk ratio, hazard ratio, difference in means) used for each outcome.	42
Synthesis methods	14	Describe the meta-analysis methods used to synthesise IPD. Specify any statistical methods and models used. Issues should include (but are not restricted to):<br>• Use of a one-stage or two-stage approach.<br>• How effect estimates were generated separately within each study and combined across studies (where applicable).<br>• Specification of one-stage models (where applicable) including how clustering of patients within studies was accounted for.<br>• Use of fixed or random effects models and any other model assumptions, such as proportional hazards.<br>• How (summary) survival curves were generated (where applicable).<br>• Methods for quantifying statistical heterogeneity (such as I^2 and τ^2).<br>• How studies providing IPD and not providing IPD were analysed together (where applicable).<br>• How missing data within the IPD were dealt with (where applicable).	43-44

Exploration of variation in effects	A2	If applicable, describe any methods used to explore variation in effects by study or participant level characteristics (such as estimation of interactions between effect and covariates). State all participant-level characteristics that were analysed as potential effect modifiers, and whether these were pre-specified.	45-48
Risk of bias across studies	15	Specify any assessment of risk of bias relating to the accumulated body of evidence, including any pertaining to not obtaining IPD for particular studies, outcomes or other variables.	reference 2
Additional analyses	16	Describe methods of any additional analyses, including sensitivity analyses. State which of these were pre-specified.	48-50
**Results**			
Study selection and IPD obtained	17	Give numbers of studies screened, assessed for eligibility, and included in the systematic review with reasons for exclusions at each stage. Indicate the number of studies and participants for which IPD were sought and for which IPD were obtained. For those studies where IPD were not available, give the numbers of studies and participants for which aggregate data were available. Report reasons for non-availability of IPD. Include a flow diagram.	7-8
Study characteristic s	18	For each study, present information on key study and participant characteristics (such as description of interventions, numbers of participants, demographic data, unavailability of outcomes, funding source, and if applicable duration of follow-up). Provide (main) citations for each study. Where applicable, also report similar study characteristics for any studies not providing IPD.	8, Table 1
IPD integrity	A3	Report any important issues identified in checking IPD or state that there were none.	reference 2
Risk of bias within studies	19	Present data on risk of bias assessments. If applicable, describe whether data checking led to the up-weighting or down-weighting of these assessments. Consider how any potential bias impacts on the robustness of meta-analysis conclusions.	reference 2
Results of individual studies	20	For each comparison and for each main outcome (benefit or harm), for each individual study report the number of eligible participants for which data were obtained and show simple summary data for each intervention group (including, where applicable, the number of events), effect estimates and confidence intervals. These may be tabulated or included on a forest plot.	Figs 1-4, supplem entary

			information
Results of syntheses	21	Present summary effects for each meta-analysis undertaken, including confidence intervals and measures of statistical heterogeneity. State whether the analysis was pre-specified, and report the numbers of studies and participants and, where applicable, the number of events on which it is based.	9-11, Figs 1-4, supplementary information
		When exploring variation in effects due to patient or study characteristics, present summary interaction estimates for each characteristic examined, including confidence intervals and measures of statistical heterogeneity. State whether the analysis was pre-specified. State whether any interaction is consistent across trials.	
		Provide a description of the direction and size of effect in terms meaningful to those who would put findings into practice.	
Risk of bias across studies	22	Present results of any assessment of risk of bias relating to the accumulated body of evidence, including any pertaining to the availability and representativeness of available studies, outcomes or other variables.	reference 2
Additional analyses	23	Give results of any additional analyses (e.g. sensitivity analyses). If applicable, this should also include any analyses that incorporate aggregate data for studies that do not have IPD. If applicable, summarise the main meta-analysis results following the inclusion or exclusion of studies for which IPD were not available.	11-16, Extended Data, supplementary information
**Discussion**			
Summary of evidence	24	Summarise the main findings, including the strength of evidence for each main outcome.	17
Strengths and limitations	25	Discuss any important strengths and limitations of the evidence including the benefits of access to IPD and any limitations arising from IPD that were not available.	22-24
Conclusions	26	Provide a general interpretation of the findings in the context of other evidence.	17-22

Implications	A4	Consider relevance to key groups (such as policy makers, service providers and service users). Consider implications for future research.	24-25
**Funding**			
Funding	27	Describe sources of funding and other support (such as supply of IPD), and the role in the systematic review of those providing such support.	29

A1 – A3 denote new items that are additional to standard PRISMA items. A4 has been created as a result of re-arranging content of the standard PRISMA statement to suit the way that systematic review IPD meta-analyses are reported.

© Reproduced with permission of the PRISMA IPD Group, which encourages sharing and reuse for non-commercial purposes
