# Supplementary material for: A meta-analysis of the long-term effects of antihypertensive therapy on the risk of major cardiovascular disease across 51 randomized trials
Source: Nat Med. 2026 Jul 1;32(8):3082–92. doi: 10.1038/s41591-026-04514-3 (PMC13472958; doi:10.1038/s41591-026-04514-3)
Supplement: Supplementary file 2 [file 41591_2026_4514_MOESM2_ESM.pdf]

nature portfolio

Corresponding author(s): Kazem Rahimi

Last updated by author(s): Qianqian Yang

Reporting Summary

Nature Portfolio wishes to improve the reproducibility of the work that we publish. This form provides structure for consistency and transparency in reporting. For further information on Nature Portfolio policies, see our Editorial Policies and the Editorial Policy Checklist.

Statistics

For all statistical analyses, confirm that the following items are present in the figure legend, table legend, main text, or Methods section.

n/a	Confirmed	
☐	☒	The exact sample size (*n*) for each experimental group/condition, given as a discrete number and unit of measurement
☐	☒	A statement on whether measurements were taken from distinct samples or whether the same sample was measured repeatedly
☐	☒	The statistical test(s) used AND whether they are one- or two-sided<br>*Only common tests should be described solely by name; describe more complex techniques in the Methods section.*
☐	☒	A description of all covariates tested
☐	☒	A description of any assumptions or corrections, such as tests of normality and adjustment for multiple comparisons
☐	☒	A full description of the statistical parameters including central tendency (e.g. means) or other basic estimates (e.g. regression coefficient) AND variation (e.g. standard deviation) or associated estimates of uncertainty (e.g. confidence intervals)
☐	☒	For null hypothesis testing, the test statistic (e.g. *F*, *t*, *r*) with confidence intervals, effect sizes, degrees of freedom and *P* value noted<br>*Give P values as exact values whenever suitable.*
☒	☐	For Bayesian analysis, information on the choice of priors and Markov chain Monte Carlo settings
☐	☒	For hierarchical and complex designs, identification of the appropriate level for tests and full reporting of outcomes
☒	☐	Estimates of effect sizes (e.g. Cohen's *d*, Pearson's *r*), indicating how they were calculated

Our web collection on statistics for biologists contains articles on many of the points above.

Software and code

Policy information about availability of computer code

Data collection	Not applicable; no new data were collected for this study
Data analysis	All statistical analyses were conducted in R 4.4.2. The main packages used for data management, statistical modelling, and visualisation included tidyverse, readxl, openxlsx, survival, rstpm2, ipw, metafor, forestplot, survminer, and patchwork.

For manuscripts utilizing custom algorithms or software that are central to the research but not yet described in published literature, software must be made available to editors and reviewers. We strongly encourage code deposition in a community repository (e.g. GitHub). See the Nature Portfolio guidelines for submitting code & software for further information.

Data

Policy information about availability of data

All manuscripts must include a data availability statement. This statement should provide the following information, where applicable:

- Accession codes, unique identifiers, or web links for publicly available datasets
- A description of any restrictions on data availability
- For clinical datasets or third party data, please ensure that the statement adheres to our policy

The governance of the BPLTTC has been reported previously.2 Individual participant data used in this study were contributed to BPLTTC by the original trial custodians under data-sharing agreements and cannot be deposited in a public repository or redistributed by BPLTTC because they are subject to confidentiality and data governance restrictions. Scientific activities based on the BPLTTC dataset are overseen by the BPLTTC steering committee. Requests for data should be

April 2023

made directly to the data custodians for each trial. Information about individual projects is posted at https://www.wrh.ox.ac.uk/research/Blood_Pressure_Lowering_Treatment_Trialists_Collaboration_BPLTTC.

Research involving human participants, their data, or biological material

Policy information about studies with human participants or human data. See also policy information about sex, gender (identity/presentation), and sexual orientation and race, ethnicity and racism.

Reporting on sex and gender	Sex was recorded at baseline in the source trials and was available as male/female in the BPLTTC individual participant dataset. Gender identity was not collected. The analysis population comprised 42% women and 58% men. Sex-stratified analyses were performed, and the findings were consistent between women and men. The manuscript has been checked to ensure that the term "sex" is used consistently.
Reporting on race, ethnicity, or other socially relevant groupings	NA
Population characteristics	Baseline population characteristics and covariates are described in the main text and Table 1.
Recruitment	Participants were recruited in individual trials in the meta-analysis as per the protocol of that trial.
Ethics oversight	Ethics approval for this phase was obtained from the Oxford Tropical Research Ethics Committee (OxTREC reference 545–14), and the analysis plan was approved by the BPLTTC steering committee and collaborators before releasing the data for analysis

Note that full information on the approval of the study protocol must also be provided in the manuscript.

Field-specific reporting

Please select the one below that is the best fit for your research. If you are not sure, read the appropriate sections before making your selection.

☒ Life sciences ☐ Behavioural & social sciences ☐ Ecological, evolutionary & environmental sciences

For a reference copy of the document with all sections, see nature.com/documents/nr-reporting-summary-flat.pdf

Life sciences study design

All studies must disclose on these points even when the disclosure is negative.

Sample size	No formal sample size calculation was performed for this secondary IPD meta-analysis. The sample size was determined by the availability of eligible randomised trials and participants in the BPLTTC dataset, according to prespecified eligibility criteria.
Data exclusions	In accordance with the prespecified exclusion criteria in the BPLTTC protocol, one trial was excluded due to a lack of time-to-event data. Participants with a known diagnosis of heart failure at trial baseline were excluded from this analysis, because established heart failure defines a clinically distinct population, with different treatment goals and risk profiles and outcome definitions from the broader hypertensive population and are therefore commonly excluded from individual antihypertensive trials.
Replication	Reproducibility was supported by a prespecified protocol, harmonised IPD, intention-to-treat analyses, and multiple sensitivity analyses. The main findings were consistent across these checks, with no non-reproducible findings identified.
Randomization	Randomization was performed by the original investigators of the studies. We assessed randomization of participants within individual studies included in the meta-analysis.
Blinding	Blinding procedures varied across the original trials. At the IPD meta-analysis stage, blinding was not relevant: data had already been collected, and investigators were not blinded during analysis because treatment allocation defined the exposure of interest.

Reporting for specific materials, systems and methods

We require information from authors about some types of materials, experimental systems and methods used in many studies. Here, indicate whether each material, system or method listed is relevant to your study. If you are not sure if a list item applies to your research, read the appropriate section before selecting a response.

Materials & experimental systems

n/a	Involved in the study
☒	☐ Antibodies
☒	☐ Eukaryotic cell lines
☒	☐ Palaeontology and archaeology
☒	☐ Animals and other organisms
☐	☒ Clinical data
☒	☐ Dual use research of concern
☒	☐ Plants

Methods

n/a	Involved in the study
☒	☐ ChIP-seq
☒	☐ Flow cytometry
☒	☐ MRI-based neuroimaging

Clinical data

Policy information about clinical studies

All manuscripts should comply with the ICMJE guidelines for publication of clinical research and a completed CONSORT checklist must be included with all submissions.

Clinical trial registration	Registration details for contributing trials are reported in the original trial publications
Study protocol	The protocol for the current analyses was reviewed and approved by the BPLTTC Steering Committee prior to data analysis
Data collection	No new data were collected.
Outcomes	Outcomes were prespecified and harmonised across trials. The primary outcome in this study was major cardiovascular events; secondary outcomes included stroke, ischaemic heart disease, heart failure, cardiovascular death and all-cause death.

Plants

Seed stocks	NA
Novel plant genotypes	NA
Authentication	NA
