# Supplementary material for: A meta-analysis of the long-term effects of antihypertensive therapy on the risk of major cardiovascular disease across 51 randomized trials
Source: Nat Med. 2026 Jul 1;32(8):3082–92. doi: 10.1038/s41591-026-04514-3 (PMC13472958; doi:10.1038/s41591-026-04514-3)
Supplement: Supplementary file 3 [file 41591_2026_4514_MOESM3_ESM.pdf]

nature portfolio

Peer Review File

A meta-analysis of the long-term effects of antihypertensive therapy on the risk of major cardiovascular disease across 51 randomised trials

Corresponding Author: Professor Kazem Rahimi

This file contains all reviewer reports in order by version, followed by all author rebuttals in order by version.

Version 0:

Reviewer comments:

Reviewer #1

(Remarks to the Author)
This is an impressively large and methodologically sophisticated dataset, and the authors take a rigorous approach to addressing an important and clinically meaningful research question that is whether the cardiovascular benefits of blood pressure–lowering therapy compound over time, with implications for the "earlier is better" treatment paradigm. The scale of the BPLTTC resource and the careful time stratified modeling allow the authors to examine this question more directly than prior work, and the clinical relevance of establishing (or refuting) temporal amplification of BP lowering benefits is substantial and impactful. Overall, this is a well refined approach and a well written manuscript.
However, the main concern or methodological challenge in such time stratified analyses is informative censoring leading to progressive changes in the risk profile of remaining participants. And thus the study feels insufficient to address both the framing and interpretation of the results. The year over year comparisons are fundamentally limited by censoring, because each time a patient experiences an event (or is lost), the composition of the analytic population changes. As shown in the supplemental tables, participants remaining at ≥5 years are younger and have markedly fewer comorbidities, indicating that the analytic cohort becomes progressively healthier and more treatment tolerant over time. Because event free and uncensored survivors represent a physiologically selected subgroup, hazard ratios estimated in later years do not reflect the same population as in earlier intervals, undermining the biological interpretability of temporal trends.
I do not have a formal statistical background, and I may not fully understand the inverse probability of censoring weighting (IPCW) approach, but it seems that although IPCW improves balance on measured covariates, it cannot account for unmeasured drivers of dropout such as intolerance to BP lowering, frailty, or evolving hemodynamics. As a result, the reported attenuation and lack of compounding benefit may primarily reflect survivor bias rather than true stability or decline in pharmacologic efficacy. The conclusions would be strengthened by explicitly acknowledging that temporal patterns in treatment effect are confounded by shifting population composition and therefore cannot directly answer whether the biological benefit of BP lowering truly changes with longer exposure.
As an additional suggestion, the authors may consider complementing the time stratified hazard ratio approach with Poisson or negative binomial regression models comparing the total count of events across follow up years, accounting for person time. But that data will most likely not be available from these clinical trials which have clear definition of events and censoring after a single event. Regardless, such models could provide a population level summary of absolute event accumulation over time that is less sensitive to yearly changes in cohort composition. While not a substitute for causal time varying modeling, a count based analysis could offer a useful secondary perspective on whether the total burden of prevented events increases, stays stable, or diminishes with longer exposure. This would be another way to look at the "compounding benefit" hypothesis that may help triangulate the findings.

Reviewer #2

(Remarks to the Author)
Thank you very much for giving me the opportunity to read this excellent manuscript by Yang et al. This analysis has several strengths. It leverages the largest available individual participant–level dataset of antihypertensive trials, standardizes effects to a clinically relevant 5-mmHg SBP reduction, and confirms findings through multiple sensitivity analyses, including IPCW to account for potential selection bias. The methodology appears to be very rigorous and consistent. Within

approximately five years of FU, the proportional hazard reduction associated with BP lowering appears to be established early and does not progressively increase during annual FU intervals.

As highlighted by the authors, the topic of whether antihypertensive therapy confers progressively increasing benefit with longer duration has been inferred indirectly from observational or Mendelian data rather than tested directly in randomized trials. In that sense, this analysis addresses an important and previously unexplored question with clinical relevance.

However, the interpretation of these findings requires caution.

First, the analyses estimate treatment effects within successive conditional risk sets. Participants contribute to the 1–2 year interval only if they survive event-free through year 1 (same reasoning for later intervals). As a result, the participants entering > 3 years are not equivalent to the baseline population. This can be seen in Table 1, where the prevalence of prior CV disease declines from approximately 44% in the first year to 26% at ≥5 years. Same observation regarding for all reported comorbidities (except CKD). Age also changes during FU. These changes suggest "depletion" of higher-risk individuals earlier in follow-up.

Second, follow-up is split into annual intervals, and treatment effects estimated in later years are conditional on survival. My concern is that the comparison of HRs across intervals therefore compares effects across evolving risk strata rather than across a stable population (in terms of CV risk): participants most susceptible to events (and also those potentially most responsive to treatment) are removed from the dataset early, leaving a lower-risk cohort in later follow-up intervals. Even if the biological effect of sustained BP reduction increased over time, the observed HR could attenuate due to changes in the composition of the population. Could the authors comment on that point?
IPCW partially addresses imbalance in measured baseline covariates across annual follow-up groups. However, it can only aacount for measured characteristics included in the model; it does not fully account for dynamic risk evolution. Thus, in my opinion, caution is warranted when interpreting temporal changes in HR.

Another point of concern is the distinction between time-varying HRs and lifetime biological effect. The analysis evaluates whether the proportional hazard reduction increases over time, which reflects changes in instantaneous relative risk. In contrast, biological effect refers to the impact of sustained BP reduction on vascular structure and long-term disease processes — concepts that cannot really be assessed within the framework of this study. In my opinion, these may be different questions. A stable or decreasing HR over time does not exclude cumulative benefit from a biological standpoint. Furthermore, the median FU of 4.2 years limits interpretation of life-course effects. Conclusions regarding “vaccine-like” prevention strategies (that the authors referred to) or the absence of compounding benefit over the long term extend well beyond this time interval.

Reviewer #3

(Remarks to the Author)
Qianqian Yang and colleagues present a paper into the duration of antihypertensive treatment and the effect on major cardiovascular outcomes based in the BPLTTC dataset of randomised clinical trials. In essence, they show that the effect of blood pressure-lowering treatment on cardiovascular disease materialises early with no further risk reduction in subsequent years (until around 5 years or a bit more).

This paper, despite some limitations, is highly relevant for clinical practice and helps physicians and patients to make informed decisions. It will probably not change the approach to treat hypertension early and aggressively in most patients but the paper provides a scientific basis for this approach and also helps to explore other options with some patients.

The data are robust, analysis is sound and the sensitivity analyses are reassuring. I do not have specific concerns or suggestions here.

I would, however, like to flag up a few other issues and ask the authors for clarification of a few specific questions.

1. The analysis is entirely based on SBP reduction. Would a DBP-based approach lead to similar findings? An analysis of the effect of DBP-lowering on at least the major combined outcome should be provided as part of supplementary material.

2. I note the adjustments for relevant potential confounders but am surprised that males and females were not studied separately in subgroup analyses. There is also no discussion on any male/female differences (or absence of such differences) in the paper. Please provide this subgroup analysis as part of the supplementary material.

3. The study focusses on traditional cardiovascular outcomes (stroke, HF, IHD) whereas CKD has not been assessed. It could be interesting to see CKD as additional outcome.

4. I would also like to understand if there are differences between those with vs without diabetes. From the adjusted models this doesn’t seem to be the case but again, subgroup or sensitivity analyses could be provided.

5. More than a third of participants were on lipid-lowering therapy. Is there any confounding by these powerful medications that also reduce CV risk? It is unlikely that e.g. statins would interact with the effect of blood pressure reduction on risk reduction but again a sensitivity analysis of participants who were not on lipid-lowering therapy could be provided.

6. The analysis by drug classes is not entirely clear to me. Many participants will be on combination therapy either because of pre-existing therapy, add-on therapy or trial designs with combination therapy. Please elaborate on how the analysis by drug class was done.

7. A more general comment, and the authors acknowledge this in the limitations section, is that outcomes that will need more time to develop (e.g. cognitive impairment) will not be captured within the median follow-up time of 4 years. In fact, if lifecourse and lifetime risk play a prominent role for any outcome it is probably cognitive impairment – we just don't have robust data in this area yet.

8. Equally generally, the mean age of study participants wasn't exactly on the young side. Conclusions about lifetime risk cannot be easily drawn from the data and this should be acknowledged. Plasticity of the cardiovascular system and effects to modify disease progression will presumably be different in a 30-year-old compared to a 65-year-old person.

9. As far as I saw, the ASCOT Legacy trial was not mentioned. Wouldn't line 410 provide an opportunity to do so?

10. The discussion on sensitivity analyses (lines 444 to 463) can be shortened as these analyses have been introduced in detail in the methods section and reported in the results section. The discussion doesn't go much deeper.

11. Having read the paper in some detail I spotted a typo in line 34 (should read "a hypothesis") and the sentence in lines 244/345 will need revision/completion.

Version 1:

Reviewer comments:

Reviewer #1

(Remarks to the Author)
Appreciate the authors' thoughtful and substantive revision that addresses the primary methodological concerns raised in my initial review. The revised manuscript now clearly acknowledges that the time stratified hazard ratios are conditional on evolving risk sets and that participants contributing to later follow up intervals represent a progressively selected, event free subset of the original randomized cohort. The expanded discussion of informative censoring, the explicit recognition of the limitations of IPCW in the presence of unmeasured dropout drivers, and more careful tempering of causal interpretation helps strengthen the framing of the results. The rigor and addition of complementary fixed horizon absolute risk analyses anchored to the original randomized population provides an important population level perspective that helps reconcile the stable or attenuated relative effects with accumulating absolute benefit over time. Still, residual survivor bias cannot be fully excluded, the authors' revised interpretation appropriately reflects this limitation and avoids over attribution of temporal patterns to treatment alone. Overall, the revisions have significantly improved the methodological transparency and interpretability of the manuscript, and my central concerns have been satisfactorily addressed.

Reviewer #2

(Remarks to the Author)
Many thanks to the authors for addressing the comments. No further comment from my side.

Reviewer #3

(Remarks to the Author)
Thank you. My comments have been addressed appropriately and the paper has further improved. I have no additional questions.

Open Access This Peer Review File is licensed under a Creative Commons Attribution 4.0 International License, which permits use, sharing, adaptation, distribution and reproduction in any medium or format, as long as you give appropriate credit to the original author(s) and the source, provide a link to the Creative Commons license, and indicate if changes were made.
In cases where reviewers are anonymous, credit should be given to 'Anonymous Referee' and the source.
The images or other third party material in this Peer Review File are included in the article's Creative Commons license, unless indicated otherwise in a credit line to the material. If material is not included in the article's Creative Commons license and your intended use is not permitted by statutory regulation or exceeds the permitted use, you will need to obtain permission directly from the copyright holder.
To view a copy of this license, visit https://creativecommons.org/licenses/by/4.0/

Your Article, "Longer duration of antihypertensive therapy and risk of major cardiovascular disease: an individual participant-level data meta-analysis of 51 randomised controlled trials" has now been seen by 3 referees. You will see from their comments below that while they find your work of interest, they have raised some substantial concerns that would need to be addressed. We would accordingly be interested in seeing a revised manuscript taking into account all reviewer and editor comments.

We feel that the referees comments are generally constructive and ask you to address them as fully as possible. In addition, we ask that you clarify in the manuscript how you envisage that these findings would have actionable implications for hypertension treatment.

Please also address the following formatting instructions:.

1. The Methods should be placed at the end of the manuscript, after the Discussion. Please edit the beginning of the Results if necessary to take into account that the reader hasn't seen the Methods yet and may need additional orientation before presenting the results.

2. Please consider relabeling the most important Supplementary Figures and Tables as Extended Data Figures and Tables (a maximum of 10 in total). The advantage of Extended Data is that these display items are in the main html file of the manuscript, rather than being in a separate Supplementary Information file.

We therefore invite you to revise your manuscript taking into account all reviewer and editor comments. Please highlight all changes in the manuscript text file.

We are committed to providing a fair and constructive peer-review process. Do not hesitate to contact us if there are specific requests from the reviewers that you believe are technically impossible or unlikely to yield a meaningful outcome.

Response to Editors

Thank you for the opportunity to revise our manuscript and for the thoughtful and constructive comments from the editors and reviewers. We have substantially revised and extended the manuscript in response to these comments. A detailed point-by-point response to the reviewers is provided below; here we summarise the principal changes made to the manuscript.

1. Clarifying the interpretation of the primary analysis and addressing concerns about changing risk sets over time

The central concern raised by the reviewers was the interpretation of the time-stratified HRs, given that participants contributing to later follow-up intervals represent evolving conditional risk sets rather than the original baseline population. We provide a detailed response to this concern below, including the

reviewer-suggested triangulation analysis, and have revised the manuscript accordingly. Specifically, we made three related changes. First, we clarified the estimand more explicitly and now state that the interval-specific estimates should be interpreted as treatment effects within evolving risk sets during randomised follow-up, rather than as effects in a fixed baseline population. And we have explained why this limitation is unlikely to fully account for the current findings. Second, we expanded the inverse probability of censoring weighted analyses by incorporating time-updated adherence, where available, to partially capture time-varying factors related to treatment persistence and changing clinical status. Third, to complement the conditional interval-specific HRs, we added fixed-horizon cumulative absolute risk reductions from randomisation to 1, 2, 3, 4, and 5 years. These estimates are anchored to the original randomised population and therefore do not condition on event-free survival within each follow-up interval. In addition, in response to the reviewer's suggestion, we conducted an exploratory count-based Poisson analysis as supportive triangulation, and we present it here in the response letter only.

Taken together, the manuscript revisions and this additional supportive analysis strengthen the interpretation of the study and help address the concern that changes in risk-set composition over time are unlikely to have materially distorted the overall temporal pattern of treatment effects. Overall, our findings do not support progressively amplifying proportional effects over time during randomised follow-up, although cumulative absolute benefit continues to accrue with longer treatment exposure.

2. Additional subgroup and alternative-standardisation analyses

As suggested by the reviewers, we added subgroup analyses by sex and diabetes status, and repeated the primary analysis after standardising treatment effects to diastolic rather than systolic blood pressure reduction. These additional analyses showed patterns consistent with the primary results and further support the robustness of the overall conclusions.

3. Refining the clinical implications of the study

As requested by the editors, we revised the manuscript to clarify the clinical implications of our findings. Specifically, our study provides direct randomised evidence on whether the relative cardiovascular benefits of blood pressure-lowering treatment progressively increase over the course of follow-up. We have clarified these implications further in the Abstract and Discussion sections as follows:

"These findings indicate that the relative cardiovascular benefits of BP–lowering emerge within the first months after treatment and do not progressively increase over time. Therefore, pharmacological initiation should be guided by baseline cardiovascular risk and expected absolute benefit, and prioritising individuals at higher cardiovascular risk yields greater clinical utility than prolonged treatment in low-risk individuals."

"These findings have important implications for cardiovascular prevention and clinical decision-making. Our results do not support the assumption that the relative effects of BP-lowering necessarily amplify with longer treatment duration

during the period of available randomised follow-up. Rather, the rationale for early and sustained treatment lies in the early establishment of relative benefit and the continued accrual of absolute risk reduction with ongoing exposure. As a result, treatment decisions should remain guided primarily by baseline cardiovascular risk and expected absolute benefit,[13] rather than by an expectation of progressively larger proportional effects over time. Combined with previous BPLTTC findings demonstrating consistent relative risk reductions across age groups,[2] and age-stratified analyses in this study, our findings do not support changing treatment thresholds solely on the assumption that earlier exposure yields progressively larger proportional benefits."

We are grateful for the editors' and reviewers' thoughtful comments, and we believe that these revisions have substantially strengthened the manuscript. We hope that the revised manuscript is now suitable for publication in *Nature Medicine*. Our detailed point-by-point responses to the reviewers' comments are provided below.

Point-by-point answer to the reviewer comments

Reviewers' Comments:

Reviewer #1 (Remarks to the Author):

Q1. This is an impressively large and methodologically sophisticated dataset, and the authors take a rigorous approach to addressing an important and clinically meaningful research question that is whether the cardiovascular benefits of blood pressure–lowering therapy compound over time, with implications for the "earlier is better" treatment paradigm. The scale of the BPLTTC resource and the careful time stratified modeling allow the authors to examine this question more directly than prior work, and the clinical relevance of establishing (or refuting) temporal amplification of BP lowering benefits is substantial and impactful. Overall, this is a well refined approach and a well written manuscript.

Re: We thank the reviewer for their favourable review, and are pleased they recognised the clinical value of our research, alongside the methodological and scaling strengths of the BPLTTC resource.

Q2. However, the main concern or methodological challenge in such time stratified analyses is informative censoring leading to progressive changes in the risk profile of remaining participants. And thus the study feels insufficient to address both the framing and interpretation of the results. The year over year

comparisons are fundamentally limited by censoring, because each time a patient experiences an event (or is lost), the composition of the analytic population changes. As shown in the supplemental tables, participants remaining at ≥5 years are younger and have markedly fewer comorbidities, indicating that the analytic cohort becomes progressively healthier and more treatment tolerant over time. Because event free and uncensored survivors represent a physiologically selected subgroup, hazard ratios estimated in later years do not reflect the same population as in earlier intervals, undermining the biological interpretability of temporal trends.

Re: We agree that interval-specific HRs are estimated within evolving conditional risk sets. This concern was extensively assessed and debated by the working group during the analysis and drafting of the study report. To provide further clarity, we have addressed this issue from several distinct perspectives:

1. We conducted a series of targeted sensitivity analyses designed to evaluate the potential impact of informative censoring and evolving cohort composition on our findings:
(a) Inverse probability of censoring weighting (IPCW) based on measured baseline covariates (**Extended Data Table 2; Extended Data Fig. 1**), which yielded results consistent with the primary analysis.
(b) An extended IPCW model incorporating time-updated adherence in the subset of trials with repeated adherence assessments. In this analysis, adherence status was updated at each follow-up visit and included as a time-varying covariate, thereby partially capturing treatment intolerance, persistence, and evolving

clinical status—factors the reviewer correctly identifies as unmeasured in the baseline-only IPCW model. The results were again consistent with the primary analysis (**Extended Data Fig. 2**).

(c) Restriction to participants with adherence ≥80% in the 12 trials with available adherence data, directly addressing the concern regarding declining treatment compliance (**Extended Data Fig. 3**).

(d) Age-stratified subgroup analyses, including younger participants (<55 years) in whom early depletion of highly susceptible individuals would be expected to be less pronounced. The temporal pattern was consistent across all age subgroups (**Fig 3**).

(e) Continuous-time modelling approaches that do not rely on pre-specified annual cut-points: a time-varying Cox model implemented within a counting-process framework showed no evidence of progressively strengthening treatment effects for major cardiovascular events (**Extended Data Table 1**). Additionally, flexible parametric survival models (PMID: 12210632) visualised the temporal pattern, further confirming the primary finding (**Fig 2**).

None of these analyses showed evidence of progressively increasing treatment effects over time. The consistency of findings across approaches with fundamentally different assumptions—including alternative modelling of follow-up time, adjustment for changes in cohort composition, and accounting for attenuation of blood pressure separation or declining adherence—makes it unlikely that the observed pattern is attributable to any single source of bias.

2. Beyond our own analyses, published evidence supports the view that depletion bias is unlikely to account for the observed pattern. Strobel and colleagues investigated built-in selection bias using individual participant data from 27 large randomised clinical trials and a Dynamic Landmarking diagnostic approach, and found no evidence of meaningful selection bias in any of them (PMID: 37410301). Abrahamowicz and colleagues demonstrated, through simulation, that important bias towards the null occurs only when unmeasured susceptibility is a very strong risk factor and cumulative event incidence is high. When incidence is below 15%—consistent with our study population—even a 10-fold unmeasured risk factor produces year-specific HR bias towards the null of no more than 18% (PMID: 40608267). These findings suggest that, at the event rates observed in our dataset, depletion of susceptible individuals may contribute to some attenuation of later period-specific HRs, but is unlikely to account for the complete absence of progressive amplification fully.

3. An important line of supporting evidence comes from the Cholesterol Treatment Trialists' (CTT) Collaboration, which applied an essentially identical time-stratified analytical framework to individual participant data from randomised trials of statin therapy. The 2005 CTT meta-analysis of 90,056 participants across 14 trials reported that proportional risk reductions were smaller in year 1 and significantly larger in subsequent years ($P < 0.0001$ for the difference between year 1 and later years; PMID: 16214597). This progressive amplification was confirmed in their expanded 2010 analysis of 170,000 participants in 26 trials (PMID: 21067804) and further detailed in a 2016 comprehensive review, which noted that increasing non-compliance over time

means the true per-mmol/L reductions are likely underestimated in later years (PMID: 27616593). This precedent demonstrates that the analytical framework employed in our study is capable of detecting progressively strengthening treatment effects when they are genuinely present—serving as a "positive control" that validates the sensitivity of the method.

Taken together, these lines of evidence—while not eliminating the inherent limitation identified by the reviewer—substantially mitigate the concern that the absence of progressive amplification is attributable solely to survivor bias or declining adherence. We have revised the manuscript accordingly. The interval-specific HRs are now framed as addressing whether the proportional treatment effect among participants remaining at risk shows evidence of progressive amplification during randomised follow-up—rather than whether the biological efficacy of blood pressure–lowering therapy changes with prolonged exposure over the life course. The following statement has been added to the Discussion:

Added text in the Limitations:

"Second, the interpretation of the time-stratified estimates is limited because participants contributing to later intervals had survived and remained event-free and uncensored in earlier intervals. Interval-specific HRs therefore reflect evolving risk sets rather than a fixed baseline population, and IPCW cannot fully account for unmeasured factors related to censoring. However, the overall pattern was similar across sensitivity analyses and was not materially altered by alternative modelling of follow-up time, changes in cohort composition, attenuation of BP separation or declining adherence. These findings make it less likely that the

absence of progressively stronger effects was explained solely by informative censoring. Third, the present analysis did not evaluate the effects of treatment on other outcomes, such as cognitive function and chronic kidney disease, over time, and further studies will be essential to clarify these patterns. Fourth, because the mean age of the trial population was over 60 years, and even the youngest subgroup (<55 years old) represented midlife rather than early adulthood, these findings should not be extrapolated directly to treatment initiated in early adulthood. However, to our knowledge, trials conducted in low-risk younger individuals are lacking."

Added text in the conclusion:

"although later interval estimates should be interpreted in the context of changing risk sets over time."

Q3. I do not have a formal statistical background, and I may not fully understand the inverse probability of censoring weighting (IPCW) approach, but it seems that although IPCW improves balance on measured covariates, it cannot account for unmeasured drivers of dropout such as intolerance to BP lowering, frailty, or evolving hemodynamics. As a result, the reported attenuation and lack of compounding benefit may primarily reflect survivor bias rather than true stability or decline in pharmacologic efficacy. The conclusions would be strengthened by explicitly acknowledging that temporal patterns in treatment effect are confounded by shifting population composition and therefore cannot directly answer whether the biological benefit of BP lowering truly changes with

longer exposure.

Re: We agree that IPCW can improve comparability with respect to measured covariates but cannot eliminate bias arising from unmeasured or time-varying determinants of selection. As noted above, we have revised the manuscript to make this point more explicit in the Limitations section:

"Interval-specific HRs therefore reflect evolving risk sets rather than a fixed baseline population, and IPCW cannot fully account for unmeasured factors related to censoring."

To examine this issue further within the constraints of the available data, we extended our sensitivity analyses. In addition to the IPCW analysis based on measured baseline covariates, we fitted an additional IPCW model in the subset of trials with repeated adherence assessments that incorporated time-updated adherence. Adherence status was updated at each follow-up visit and included as a time-varying covariate, thereby partially capturing treatment persistence and related factors not reflected in the baseline-only IPCW model.

As shown in **Extended Data Fig. 2**, the results were consistent with the primary analysis and did not show progressively increasing proportional benefits over time. For major cardiovascular events, HRs were similar across follow-up intervals, ranging from 0.85 (95% CI, 0.80–0.91) in year 1 to 0.87 (0.74–1.01) beyond 5 years, with no evidence of a linear trend towards stronger benefit over time (P for trend = 0.072). Similar patterns were observed for stroke, ischaemic heart

disease, cardiovascular death and all-cause death. Heart failure was the main exception, showing a stronger early effect that attenuated over time (P for trend < 0.001).

Extended Data Fig. 2. Standardised effects of systolic blood pressure-lowering treatment on outcomes, stratified by follow-up duration, using inverse probability of censoring weighting, incorporating time-varying adherence

Follow-up period	Treatment group (n/N)	Incidence rate (%)	Control group (n/N)	Incidence rate (%)	HR (95% CI) per 5 mm Hg SBP reduction
**Major cardiovascular events**					
0-1 years follow-up	1,511 /39,568	3.8%	2,377 /50,750	4.7%	0.85 (0.80, 0.91)
1-2 years follow-up	1,318 /37,514	3.5%	1,995 /47,706	4.2%	0.88 (0.82, 0.95)
2-3 years follow-up	1,196 /35,501	3.4%	1,724 /44,939	3.8%	0.91 (0.84, 0.98)
3-4 years follow-up	1,038 /31,701	3.3%	1,452 /38,911	3.7%	0.90 (0.83, 0.97)
4-5 years follow-up	719 /25,073	2.9%	911 /30,210	3.0%	0.95 (0.85, 1.06)
>5 years follow-up	453 /15,176	3.0%	621 /17,812	3.5%	0.87 (0.74, 1.01)
All follow-up	6,235 /39,568	15.8%	9,080 /50,750	17.9%	0.89 (0.86, 0.92)
					P for trend = 0.072
**Ischaemic heart disease**					
0-1 years follow-up	932 /39,569	2.4%	1,413 /50,751	2.8%	0.93 (0.85, 1.02)
1-2 years follow-up	833 /37,926	2.2%	1,284 /48,442	2.7%	0.92 (0.84, 1.01)
2-3 years follow-up	758 /36,211	2.1%	1,111 /46,124	2.4%	0.93 (0.84, 1.03)
3-4 years follow-up	675 /32,588	2.1%	939 /40,365	2.3%	0.95 (0.86, 1.06)
4-5 years follow-up	487 /25,927	1.9%	595 /31,622	1.9%	1.07 (0.93, 1.23)
>5 years follow-up	328 /15,759	2.1%	466 /18,768	2.5%	0.89 (0.75, 1.07)
All follow-up	4,013 /39,569	10.1%	5,808 /50,751	11.4%	0.94 (0.90, 0.99)
					P for trend = 0.339
**Stroke**					
0-1 years follow-up	417 /39,568	1.1%	598 /50,753	1.2%	0.87 (0.76, 0.99)
1-2 years follow-up	336 /38,309	0.9%	558 /49,106	1.1%	0.75 (0.65, 0.86)
2-3 years follow-up	309 /36,958	0.8%	447 /47,329	0.9%	0.84 (0.73, 0.98)
3-4 years follow-up	269 /33,623	0.8%	402 /41,969	1.0%	0.81 (0.69, 0.95)
4-5 years follow-up	163 /27,212	0.6%	233 /33,475	0.7%	0.81 (0.65, 1.00)
>5 years follow-up	123 /16,912	0.7%	148 /20,353	0.7%	0.90 (0.65, 1.23)
All follow-up	1,617 /39,568	4.1%	2,386 /50,753	4.7%	0.84 (0.78, 0.89)
					P for trend = 0.595
**Heart failure**					
0-1 years follow-up	218 /37,257	0.6%	531 /48,417	1.1%	0.55 (0.47, 0.65)
1-2 years follow-up	253 /36,199	0.7%	359 /46,853	0.8%	0.92 (0.78, 1.08)
2-3 years follow-up	248 /34,968	0.7%	368 /45,289	0.8%	0.91 (0.77, 1.07)
3-4 years follow-up	226 /31,905	0.7%	337 /40,181	0.8%	0.85 (0.71, 1.01)
4-5 years follow-up	164 /26,204	0.6%	249 /32,325	0.8%	0.91 (0.72, 1.15)
>5 years follow-up	135 /16,514	0.8%	173 /19,768	0.9%	1.09 (0.81, 1.46)
All follow-up	1,244 /37,257	3.3%	2,017 /48,417	4.2%	0.82 (0.76, 0.88)
					P for trend < 0.001
**Cardiovascular death**					
0-1 years follow-up	291 /39,569	0.7%	417 /50,754	0.8%	0.90 (0.78, 1.05)
1-2 years follow-up	314 /38,638	0.8%	484 /49,604	1.0%	0.87 (0.75, 1.01)
2-3 years follow-up	368 /37,529	1.0%	495 /48,226	1.0%	0.99 (0.86, 1.14)
3-4 years follow-up	359 /34,362	1.0%	472 /43,096	1.1%	0.99 (0.85, 1.14)
4-5 years follow-up	247 /27,939	0.9%	369 /34,567	1.1%	0.84 (0.70, 1.00)
>5 years follow-up	225 /17,405	1.3%	291 /21,074	1.4%	0.94 (0.74, 1.20)
All follow-up	1,804 /39,569	4.6%	2,528 /50,754	5.0%	0.93 (0.88, 0.99)
					P for trend = 0.503
**All-cause death**					
0-1 years follow-up	521 /39,569	1.3%	716 /50,754	1.4%	0.96 (0.86, 1.08)
1-2 years follow-up	696 /38,638	1.8%	964 /49,604	1.9%	0.97 (0.88, 1.07)
2-3 years follow-up	812 /37,529	2.2%	1,114 /48,226	2.3%	0.97 (0.88, 1.06)
3-4 years follow-up	862 /34,362	2.5%	1,059 /43,096	2.5%	1.03 (0.94, 1.13)
4-5 years follow-up	642 /27,939	2.3%	856 /34,567	2.5%	0.93 (0.83, 1.05)
>5 years follow-up	570 /17,405	3.3%	660 /21,074	3.1%	1.05 (0.90, 1.22)
All follow-up	4,103 /39,569	10.4%	5,369 /50,754	10.6%	0.99 (0.95, 1.04)
					P for trend = 0.109

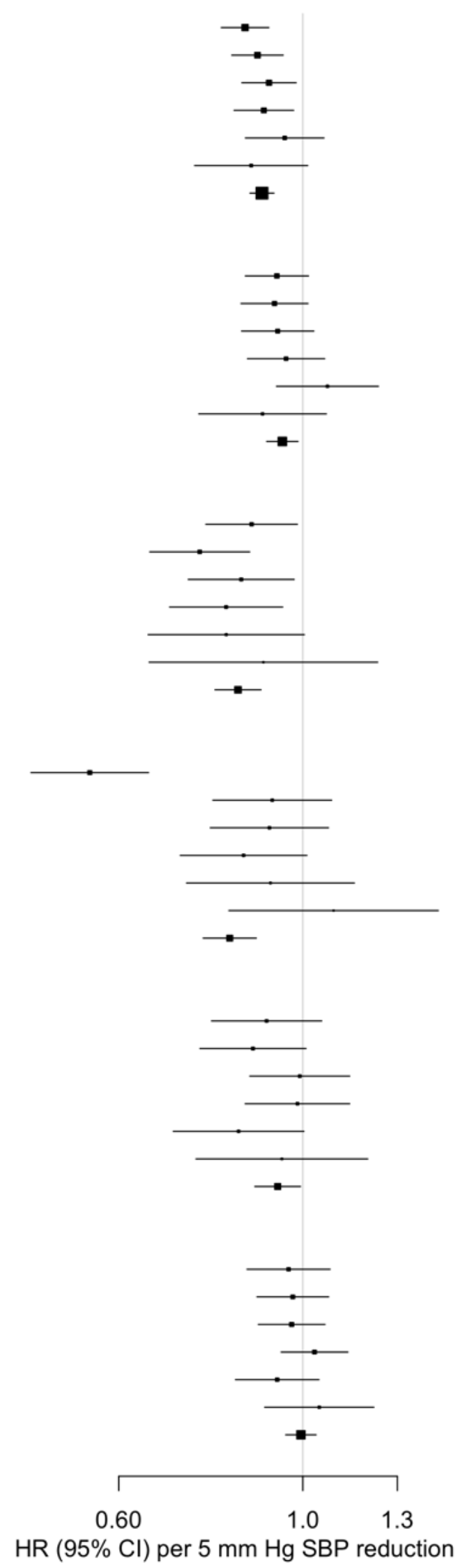

Q4. As an additional suggestion, the authors may consider complementing the time stratified hazard ratio approach with Poisson or negative binomial regression models comparing the total count of events across follow up years, accounting for person time. But that data will most likely not be available from these clinical trials which have clear definition of events and censoring after a single event. Regardless, such models could provide a population level summary of absolute event accumulation over time that is less sensitive to yearly changes in cohort composition. While not a substitute for causal time varying modeling, a count based analysis could offer a useful secondary perspective on whether the total burden of prevented events increases, stays stable, or diminishes with longer exposure. This would be another way to look at the "compounding benefit" hypothesis that may help triangulate the findings.

Re: We thank the reviewer for this suggestion. Count-based models can provide a useful complementary perspective on this question because they assess treatment effects on a different scale, namely event rates or event burden over person-time, rather than relying solely on interval-specific HRs.

However, as the reviewer anticipated, most included trials were designed around time-to-first-event analyses, with follow-up for a given endpoint commonly censored after the first occurrence. Indeed, only one trial (MOSES) explicitly documented recurrent cardiovascular events in a way that would support a formal recurrent-event analysis. Therefore, a harmonised recurrent-event Poisson or negative binomial analysis across the full BPLTTC dataset was not feasible.

To address the reviewer's suggestion as closely as the available data allowed, we conducted an exploratory Poisson analysis based on counts of component events. Instead of treating major cardiovascular events solely as a time-to-first-event composite, we used the four component outcomes underlying this composite—ischaemic heart disease, stroke, heart failure, and cardiovascular death— to approximate cumulative cardiovascular event burden. For each participant within each follow-up interval, we counted the number of these component outcomes that occurred. Under this approach, participants could contribute more than one event over follow-up if they experienced different component outcomes at different times.

Then, the analysis was based on event rates rather than raw event counts alone. In each follow-up interval, each participant contributed the time they were actually under observation during that interval, up to either the end of the interval or the end of their observed follow-up, whichever came first. The Poisson model then compared the number of events counted relative to the accumulated follow-up time, thereby estimating incidence rate ratios (IRRs) rather than simply comparing crude totals. As in our primary analyses, treatment effects were standardised per 5 mmHg reduction in systolic blood pressure, and the trial was accounted for in the model.

As expected, this broader component-based definition increased the number of counted events relative to the original time-to-first-event composite. Overall, the total number of major cardiovascular events increased from 43,476 under the original first-event definition to 56,071 under the approximate component-count

definition. The corresponding interval-specific counts are shown in the following **Table R1**.

Table R1. Number of participants and events in each follow-up interval

Follow-up interval	Treatment participants, n	Treatment events, n	Treatment event rate, %	Control participants, n	Control events, n	Control event rate, %
**Original major cardiovascular disease outcome (time-to-first-event composite)**						
0–1 years	168,980	5,139	3.0	188,334	6,717	3.6
1–2 years	158,669	4,238	2.7	176,296	5,464	3.1
2–3 years	146,108	3,624	2.5	162,675	4,490	2.8
3–4 years	120,181	2,909	2.4	134,429	3,822	2.8
4–5 years	86,599	1,976	2.3	98,349	2,375	2.4
>5 years	39,286	1,223	3.1	43,392	1,468	3.4
Overall	168,980	19,142	11.3	188,334	24,334	12.9
**Approximate component-count definition used in the exploratory Poisson analysis**						
0–1 years	169,190	6,065	3.6	188,537	7,968	4.2
1–2 years	162,546	5,232	3.2	181,494	6,858	3.8
2–3 years	152,441	4,661	3.1	171,182	5,945	3.5
3–4 years	127,802	3,916	3.1	144,557	5,197	3.6
4–5 years	94,043	2,701	2.9	108,590	3,454	3.2
>5 years	43,602	1,838	4.2	49,186	2,236	4.5
Overall	169,190	24,413	14.4	188,537	31,658	16.8

The results of this complementary count-based analysis were consistent with our primary time-stratified Cox analysis. The overall incidence rate ratio per 5 mmHg reduction in systolic blood pressure was 0.90 (95% CI 0.89 to 0.92). The interval-

specific incidence rate ratios were as follows: 0–1 years, 0.88 (95% CI, 0.85 to 0.91); 1–2 years, 0.88 (95% CI, 0.85 to 0.92); 2–3 years, 0.93 (95% CI, 0.90 to 0.97); 3–4 years, 0.89 (95% CI, 0.85 to 0.93); 4–5 years, 0.93 (95% CI, 0.89 to 0.99); and >5 years, 0.96 (95% CI, 0.90 to 1.02). There was a trend towards attenuation over time (P for trend = 0.026) (**Figure R1**). Thus, although approximate, this analysis provided the population-level count-based triangulation suggested by the reviewer and did not substantively alter the interpretation of the main findings.

Figure R1. Exploratory Poisson analysis of component-count major cardiovascular events across follow-up intervals

Follow-up period	Treatment events / person-time	Incidence rate (/1000 person-years)	Control events / person-time	Incidence rate (/1000 person-years)	IRR (95% CI) per 5 mm Hg SBP reduction
0-1 years follow-up	6,065 / 60,557,637	36.6	7,968 / 67,550,774	43.1	0.88 (0.85, 0.91)
1-2 years follow-up	5,232 / 57,975,554	33.0	6,858 / 64,807,121	38.7	0.88 (0.85, 0.92)
2-3 years follow-up	4,661 / 51,188,518	33.3	5,945 / 57,722,845	37.6	0.93 (0.90, 0.97)
3-4 years follow-up	3,916 / 40,783,527	35.1	5,197 / 46,525,542	40.8	0.89 (0.85, 0.93)
4-5 years follow-up	2,701 / 24,928,851	39.6	3,454 / 28,990,753	43.5	0.93 (0.89, 0.99)
>5 years follow-up	1,838 / 17,282,915	38.8	2,236 / 18,314,492	44.6	0.96 (0.90, 1.02)
**All follow-up**	**24,413 / 252,717,001**	**35.3**	**31,658 / 283,911,526**	**40.7**	**0.90 (0.89, 0.92)**

P for trend = 0.026

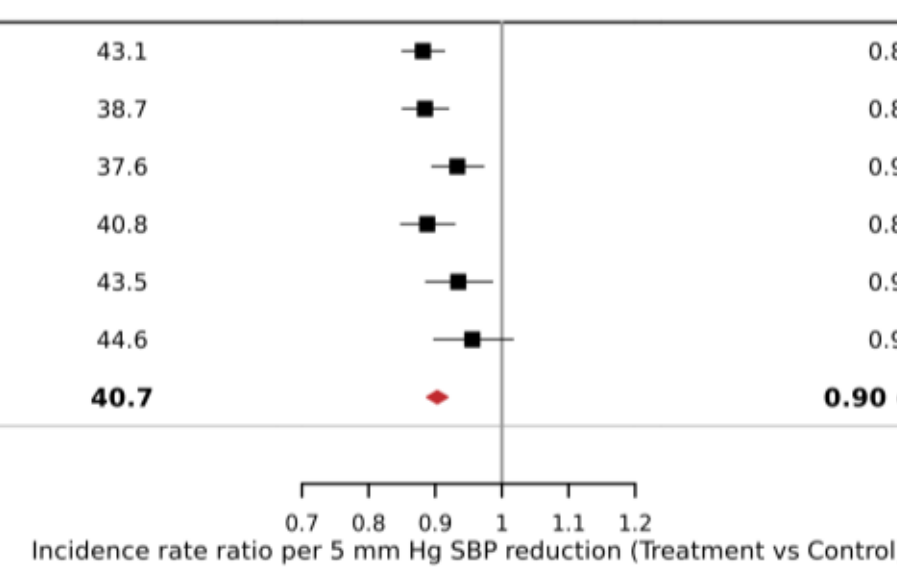

Although informative, we note that even if recurrent-event data had been uniformly available, count-based models would not resolve the key interpretational issue raised in the review. Later follow-up intervals would still include participants who remained alive and under observation, and the clinical risk profile after a first event differs importantly from that before it. Accordingly, recurrent-event count models can be useful for triangulation, but they do not eliminate concerns about informative censoring or the changing composition of risk sets over time. For these reasons, we have used this analysis as a supportive

sensitivity analysis in response to the reviewer's suggestion, rather than incorporating it as a primary result into the main manuscript.

To address the reviewer's broader objective of complementing interval-specific HR analyses with estimands anchored to the original randomised population and quantifying cumulative absolute benefit over time, we added fixed-horizon analyses from randomisation to 1, 2, 3, 4 and 5 years on the cumulative absolute-risk scale. These baseline-anchored estimands show that absolute benefit increases with longer exposure. For major cardiovascular events, the standardised absolute risk reduction increased from 0.30% at 1 year (NNT 337) to 1.25% at 5 years (NNT 80). Absolute benefits also accumulated over time for stroke, cardiovascular death and all-cause death, whereas reductions for ischaemic heart disease were smaller and estimates for heart failure were more modest and less consistent across time horizons (**Extended Data Table 3**).

We have incorporated these results into the manuscript and revised the Discussion accordingly, as follows:

"Importantly, the absence of progressively increasing proportional effects should not be interpreted as evidence of no cumulative absolute treatment benefit. In complementary fixed-horizon analyses anchored to the original randomised population, cumulative absolute risk reductions increased steadily over time for major cardiovascular events and several secondary outcomes. These analyses provide an important complement to the interval-specific HRs because they are estimated on the absolute scale and do not condition on event-free survival within

successive follow-up intervals. Together, the relative- and absolute-scale results suggest that BP-lowering treatment confers protection early, and that continued exposure allows this benefit to accumulate over time in absolute terms but without progressive amplification of proportional effects."

Extended Data Table 3. Fixed-horizon absolute risk reduction and time-specific number needed to treat, standardised to a 5-mmHg lower systolic blood pressure

Outcome	Time horizon	Standardised ARR, % (95% CI)	Number needed to treat	Included trials
Major cardiovascular events	0–1 year	0.30 (0.20 to 0.40)	337	50
	0–2 years	0.59 (0.45 to 0.74)	169	49
	0–3 years	0.74 (0.57 to 0.91)	135	47
	0–4 years	1.11 (0.88 to 1.33)	91	46
	0–5 years	1.25 (0.94 to 1.57)	80	36
Stroke	0–1 year	0.07 (0.02 to 0.12)	1535	50
	0–2 years	0.17 (0.10 to 0.24)	596	49
	0–3 years	0.26 (0.17 to 0.35)	385	47
	0–4 years	0.38 (0.27 to 0.50)	261	46
	0–5 years	0.52 (0.36 to 0.68)	192	36
Ischaemic heart disease	0–1 year	0.05 (−0.02 to 0.11)	—	48
	0–2 years	0.16 (0.07 to 0.24)	642	47
	0–3 years	0.19 (0.08 to 0.29)	531	46
	0–4 years	0.25 (0.12 to 0.38)	394	45
	0–5 years	0.20 (0.03 to 0.38)	497	35
Heart failure	0–1 year	0.08 (0.04 to 0.12)	1312	39
	0–2 years	0.07 (0.02 to 0.12)	1530	40
	0–3 years	0.06 (−0.00 to 0.11)	—	39

	0–4 years	0.10 (0.02 to 0.17)	1057	38
	0–5 years	0.05 (−0.03 to 0.13)	—	30
Cardiovascular death	0–1 year	0.04 (−0.00 to 0.08)	—	47
	0–2 years	0.11 (0.04 to 0.17)	925	46
	0–3 years	0.16 (0.07 to 0.24)	633	45
	0–4 years	0.20 (0.09 to 0.32)	493	44
	0–5 years	0.29 (0.12 to 0.47)	342	34
All-cause death	0–1 year	0.10 (0.04 to 0.17)	987	49
	0–2 years	0.17 (0.07 to 0.27)	578	48
	0–3 years	0.27 (0.13 to 0.40)	375	46
	0–4 years	0.38 (0.20 to 0.55)	266	45
	0–5 years	0.36 (0.11 to 0.62)	275	35

Reviewer #2 (Remarks to the Author):

Q5. Thank you very much for giving me the opportunity to read this excellent manuscript by Yang et al. This analysis has several strengths. It leverages the largest available individual participant–level dataset of antihypertensive trials, standardizes effects to a clinically relevant 5-mmHg SBP reduction, and confirms findings through multiple sensitivity analyses, including IPCW to account for potential selection bias. The methodology appears to be very rigorous and consistent. Within approximately five years of FU, the proportional hazard reduction associated with BP lowering appears to be established early and does not progressively increase during annual FU intervals.

As highlighted by the authors, the topic of whether antihypertensive therapy

confers progressively increasing benefit with longer duration has been inferred indirectly from observational or Mendelian data rather than tested directly in randomized trials. In that sense, this analysis addresses an important and previously unexplored question with clinical relevance.

Re: We thank the reviewer for these positive and thoughtful comments.

However, the interpretation of these findings requires caution.

Q6. First, the analyses estimate treatment effects within successive conditional risk sets. Participants contribute to the 1–2 year interval only if they survive event-free through year 1 (same reasoning for later intervals). As a result, the participants entering > 3 years are not equivalent to the baseline population. This can be seen in Table 1, where the prevalence of prior CV disease declines from approximately 44% in the first year to 26% at ≥5 years. Same observation regarding for all reported comorbidities (except CKD). Age also changes during FU. These changes suggest "depletion" of higher-risk individuals earlier in follow-up.

Second, follow-up is split into annual intervals, and treatment effects estimated in later years are conditional on survival. My concern is that the comparison of HRs across intervals therefore compares effects across evolving risk strata rather than across a stable population (in terms of CV risk): participants most susceptible to events (and also those potentially most responsive to treatment) are removed from the dataset early, leaving a lower-risk cohort in later follow-

up intervals. Even if the biological effect of sustained BP reduction increased over time, the observed HR could attenuate due to changes in the composition of the population. Could the authors comment on that point?

Re: We agree that interval-specific HRs are estimated within evolving risk sets, and that depletion of higher-risk participants may affect their interpretation over time. This issue was carefully considered and extensively discussed by the working group during the analysis and interpretation of the results, and the same concern was also raised by Reviewer 1.

However, several complementary analyses, together with supporting evidence from previous studies, suggest that although this limitation cannot be eliminated, the absence of progressive amplification is unlikely to be explained solely by survivor bias or changing cohort composition. In addition, the exploratory count-based Poisson analysis suggested by Reviewer 1, which modelled rates of component cardiovascular events over person-time, provided supportive triangulation and yielded results consistent with the primary findings.

We have therefore revised the manuscript to acknowledge this limitation more explicitly and to clarify that interval-specific estimates should be interpreted as treatment effects within evolving risk sets during randomised follow-up. For the full details of these revisions and analyses, please see our response to Reviewer 1, Q2.

Q7. IPCW partially addresses imbalance in measured baseline covariates across

annual follow-up groups. However, it can only aacount for measured characteristics included in the model; it does not fully account for dynamic risk evolution. Thus, in my opinion, caution is warranted when interpreting temporal changes in HR.

Re: We made two revisions in response. First, we expanded the IPCW analysis by adding time-updated adherence in the subset of trials with repeated adherence assessments. Specifically, we fitted an additional IPCW model that incorporated adherence as a time-varying covariate, potentially capturing factors related to treatment persistence not captured by the baseline-only model. Second, we revised the manuscript to acknowledge this limitation in the Limitations section explicitly.

As this concern overlaps with that raised by Reviewer 1, please see our response to Reviewer 1, Q2, for full details.

Q8. Another point of concern is the distinction between time-varying HRs and lifetime biological effect. The analysis evaluates whether the proportional hazard reduction increases over time, which reflects changes in instantaneous relative risk. In contrast, biological effect refers to the impact of sustained BP reduction on vascular structure and long-term disease processes — concepts that cannot really be assessed within the framework of this study. In my opinion, these may be different questions. A stable or decreasing HR over time does not exclude cumulative benefit from a biological standpoint. Furthermore, the median FU of 4.2 years limits interpretation of life-course effects.

Re: Even if the proportional treatment effect on cardiovascular events remains constant over time, as observed in this study, sustained blood pressure lowering may still influence vascular remodelling or disease progression in ways that could lead to delayed clinical benefit, as the reviewer pointed out.

We note in the Discussion that this question may be partly informed by previous studies evaluating possible legacy effects. However, the available evidence remains mixed: some post-trial follow-up studies have suggested persistent benefit, whereas others have not confirmed this pattern. Taken together, our findings should be interpreted as indicating no evidence of progressively stronger proportional effects during the period of available randomised follow-up, rather than excluding the possibility of longer-term cumulative biological benefit.

We have acknowledged this in the Limitations section: "*First, trials included in this study had a median follow-up of only 4.2 years, potentially insufficient to detect longer-term benefits. Considering that BP-lowering therapy may prevent or delay hypertension-induced pathological changes such as atherosclerosis,[33] left ventricular hypertrophy,[34] and carotid intima-media thickening,[35], longer-term evidence is needed to fully elucidate any potential compounding effects. Since trials typically end early once benefit is proven, post-trial extensions are necessary to assess long-term outcomes*."

Q9. Conclusions regarding "vaccine-like" prevention strategies (that the authors referred to) or the absence of compounding benefit over the long term extend well beyond this time interval.

Re: We agree with your suggestion, and we have therefore removed the term from the Discussion. We refer to this analogy only because it has been used in some contexts (PMID: 23729329) to frame the rationale for earlier pharmacological prevention, not because our study directly evaluates lifelong or vaccine-like effects.

We now mention it only briefly in the Introduction as follows.

"On this basis, earlier and more proactive BP-lowering strategies have been proposed, including age-specific treatment thresholds[13] and broader life-course prevention paradigms. Such approaches have sometimes been framed metaphorically as 'vaccine-like', in the sense that BP- and lipid-lowering interventions initiated earlier in life may confer long-term cardiovascular protection,[14] on the premise that earlier intervention may yield disproportionately larger relative risk reductions over the life course.[15]"

Reviewer #3 (Remarks to the Author):

Q10. Qianqian Yang and colleagues present a paper into the duration of antihypertensive treatment and the effect on major cardiovascular outcomes based in the BPLTTC dataset of randomized clinical trials. In essence, they show

that the effect of blood pressure-lowering treatment on cardiovascular disease materializes early with no further risk reduction in subsequent years (until around 5 years or a bit more).

This paper, despite some limitations, is highly relevant for clinical practice and helps physicians and patients to make informed decisions. It will probably not change the approach to treat hypertension early and aggressively in most patients but the paper provides a scientific basis for this approach and also helps to explore other options with some patients.

The data are robust, analysis is sound and the sensitivity analyses are reassuring. I do not have specific concerns or suggestions here.

Re: We thank the reviewer for the positive assessment of our work and for these constructive suggestions, which have helped us strengthen the manuscript.

I would, however, like to flag up a few other issues and ask the authors for clarification of a few specific questions.

Q11. The analysis is entirely based on SBP reduction. Would a DBP-based approach lead to similar findings? An analysis of the effect of DBP-lowering on at least the major combined outcome should be provided as part of supplementary material.

Re: Our primary analyses were standardised to systolic blood pressure because this is the most widely used and clinically interpretable measure for cardiovascular risk stratification, and in the predominantly middle-aged and older populations represented in these trials, it is generally a stronger predictor of cardiovascular outcomes than diastolic blood pressure (PMID: 41212942; PMID: 39210715). This approach is also consistent with previous BPLTTC analyses (PMID: 34461040). However, we agree that a diastolic blood pressure-based analysis provides an important complementary check.

As the mean diastolic blood pressure reduction between randomised groups, after excluding the first 12 months, was 3 mm Hg (2.9–3.0) across all trials that aimed to achieve a between-group difference in blood pressure, we repeated the primary composite-outcome analysis after standardising treatment effects to a 3 mm Hg reduction in diastolic blood pressure and added the results to the **Supplementary Fig. 5**. As shown in the following Figure, the diastolic blood pressure-standardised analysis showed a temporal pattern similar to that of the main analysis: treatment benefit was established early after initiation and did not progressively increase with longer follow-up.

Supplementary Fig. 5. Standardised effects of diastolic blood pressure lowering per 3 mm Hg reduction on outcomes, stratified by follow-up duration

Follow-up period	Treatment group (n/N)	Incidence rate (%)	Control group (n/N)	Incidence rate (%)	HR (95% CI) per 3 mm Hg DBP reduction
**Major cardiovascular events**					
0-1 years follow-up	5,139 /161,462	3.2%	6,717 /180,661	3.7%	0.88 (0.84, 0.92)
1-2 years follow-up	4,238 /151,314	2.8%	5,464 /168,769	3.2%	0.86 (0.82, 0.90)
2-3 years follow-up	3,642 /138,913	2.6%	4,490 /155,317	2.9%	0.91 (0.86, 0.97)
3-4 years follow-up	2,909 /117,006	2.5%	3,822 /131,115	2.9%	0.83 (0.78, 0.89)
4-5 years follow-up	1,991 /86,377	2.3%	2,373 /98,124	2.4%	0.92 (0.85, 1.00)
>5 years follow-up	1,223 /39,286	3.1%	1,468 /43,392	3.4%	0.90 (0.83, 0.98)
					P for trend = 0.021
All follow-up	19,142 /161,462	11.9%	24,334 /180,661	13.5%	0.88 (0.86, 0.90)
**Ischaemic heart disease**					
0-1 years follow-up	2,339 /161,459	1.4%	3,056 /180,691	1.7%	0.92 (0.86, 0.99)
1-2 years follow-up	1,977 /153,303	1.3%	2,689 /171,518	1.6%	0.87 (0.80, 0.94)
2-3 years follow-up	1,806 /142,116	1.3%	2,270 /159,615	1.4%	0.95 (0.87, 1.03)
3-4 years follow-up	1,456 /120,676	1.2%	1,977 /136,028	1.5%	0.84 (0.76, 0.93)
4-5 years follow-up	1,029 /89,790	1.1%	1,253 /102,907	1.2%	0.92 (0.81, 1.04)
>5 years follow-up	756 /40,936	1.8%	940 /45,706	2.1%	0.88 (0.79, 0.98)
					P for trend = 0.677
All follow-up	9,363 /161,459	5.8%	12,185 /180,691	6.7%	0.90 (0.87, 0.94)
**Stroke**					
0-1 years follow-up	1,932 /161,461	1.2%	2,447 /180,638	1.4%	0.87 (0.82, 0.93)
1-2 years follow-up	1,472 /153,476	1.0%	1,884 /171,811	1.1%	0.82 (0.76, 0.89)
2-3 years follow-up	1,150 /142,671	0.8%	1,493 /160,535	0.9%	0.83 (0.76, 0.91)
3-4 years follow-up	899 /121,910	0.7%	1,211 /137,689	0.9%	0.80 (0.71, 0.90)
4-5 years follow-up	648 /91,558	0.7%	817 /105,160	0.8%	0.88 (0.78, 0.99)
>5 years follow-up	425 /42,299	1.0%	494 /47,394	1.0%	0.92 (0.81, 1.06)
					P for trend = 0.305
All follow-up	6,526 /161,461	4.0%	8,346 /180,638	4.6%	0.85 (0.82, 0.88)
**Heart failure**					
0-1 years follow-up	764 /146,468	0.5%	1,239 /166,018	0.7%	0.78 (0.69, 0.88)
1-2 years follow-up	731 /139,599	0.5%	933 /158,341	0.6%	0.91 (0.80, 1.03)
2-3 years follow-up	682 /129,663	0.5%	900 /148,042	0.6%	0.93 (0.82, 1.07)
3-4 years follow-up	628 /109,992	0.6%	820 /126,271	0.6%	0.87 (0.74, 1.02)
4-5 years follow-up	380 /82,287	0.5%	528 /96,470	0.5%	0.90 (0.74, 1.10)
>5 years follow-up	218 /36,019	0.6%	263 /41,515	0.6%	0.98 (0.79, 1.21)
					P for trend < 0.001
All follow-up	3,403 /146,468	2.3%	4,683 /166,018	2.8%	0.88 (0.83, 0.93)
**Cardiovascular death**					
0-1 years follow-up	1,064 /159,988	0.7%	1,286 /179,530	0.7%	0.91 (0.82, 1.00)
1-2 years follow-up	1,116 /153,571	0.7%	1,410 /172,708	0.8%	0.89 (0.80, 0.98)
2-3 years follow-up	1,090 /143,884	0.8%	1,336 /162,859	0.8%	0.95 (0.86, 1.06)
3-4 years follow-up	992 /123,704	0.8%	1,252 /140,691	0.9%	0.90 (0.80, 1.00)
4-5 years follow-up	694 /93,092	0.7%	903 /107,981	0.8%	0.87 (0.76, 1.00)
>5 years follow-up	526 /42,910	1.2%	642 /48,834	1.3%	0.93 (0.82, 1.05)
					P for trend = 0.495
All follow-up	5,482 /159,988	3.4%	6,829 /179,530	3.8%	0.91 (0.87, 0.95)
**All-cause death**					
0-1 years follow-up	2,231 /161,176	1.4%	2,714 /180,319	1.5%	0.92 (0.86, 0.98)
1-2 years follow-up	2,812 /154,774	1.8%	3,328 /173,528	1.9%	0.96 (0.90, 1.02)
2-3 years follow-up	2,854 /144,904	2.0%	3,515 /163,460	2.2%	0.92 (0.86, 0.99)
3-4 years follow-up	2,644 /124,423	2.1%	3,234 /141,036	2.3%	0.91 (0.84, 0.97)
4-5 years follow-up	1,844 /93,795	2.0%	2,275 /108,327	2.1%	0.97 (0.88, 1.06)
>5 years follow-up	1,329 /43,599	3.0%	1,484 /49,176	3.0%	0.99 (0.91, 1.07)
					P for trend = 0.090
All follow-up	13,714 /161,176	8.5%	16,550 /180,319	9.2%	0.94 (0.91, 0.97)

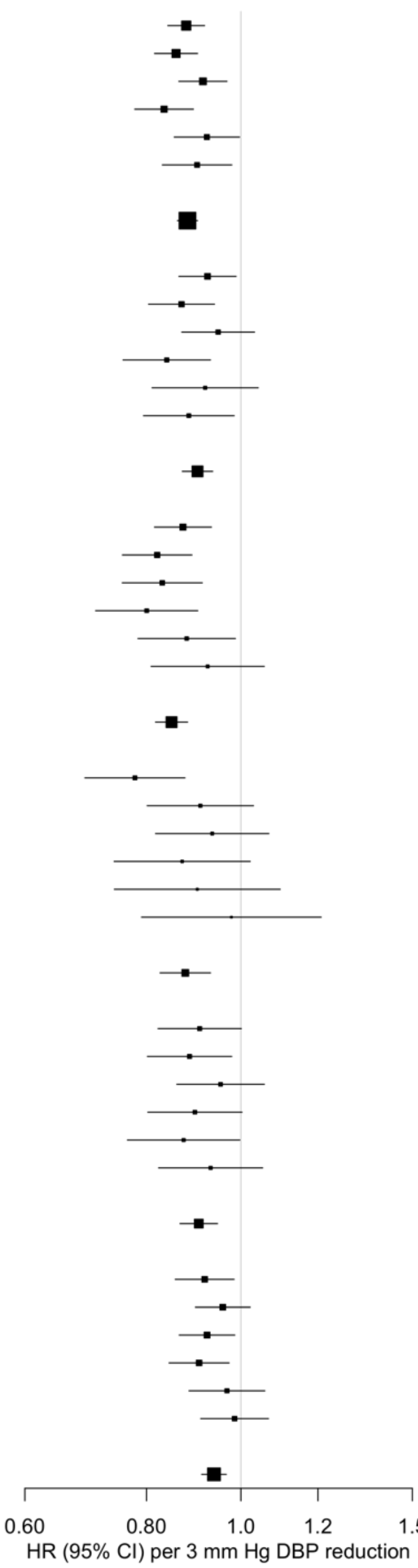

Q12. I note the adjustments for relevant potential confounders but am surprised that males and females were not studied separately in subgroup analyses. There is also no discussion on any male/female differences (or absence of such differences) in the paper. Please provide this subgroup analysis as part of the supplementary material.

Re: We conducted sex-stratified subgroup analyses, and the results have been added to the Supplementary Information. As shown in **Supplementary Figs. 11-12**, the temporal pattern was similar in women and men and consistent with the primary findings, with no evidence that the effect of blood pressure lowering varied over time according to sex.

Supplementary Fig. 11. Standardised effects of systolic blood pressure treatment on outcomes among men, stratified by follow-up duration

Follow-up period	Treatment group (n/N)	Incidence rate (%)	Control group (n/N)	Incidence rate (%)	HR (95% CI) per 5 mm Hg SBP reduction
**Major cardiovascular events**					
0-1 years follow-up	3,383 /98,484	3.4%	4,331 /110,290	3.9%	0.89 (0.85, 0.94)
1-2 years follow-up	2,748 /92,388	3.0%	3,490 /103,239	3.4%	0.90 (0.85, 0.95)
2-3 years follow-up	2,321 /84,977	2.7%	2,976 /95,168	3.1%	0.89 (0.84, 0.95)
3-4 years follow-up	1,924 /70,852	2.7%	2,495 /79,790	3.1%	0.87 (0.82, 0.93)
4-5 years follow-up	1,255 /52,054	2.4%	1,489 /59,600	2.5%	0.96 (0.89, 1.04)
>5 years follow-up	757 /22,887	3.3%	922 /25,359	3.6%	0.93 (0.85, 1.03)
					P for trend = 0.111
All follow-up	12,388 /98,484	12.6%	15,703 /110,290	14.2%	0.90 (0.88, 0.92)
**Ischaemic heart disease**					
0-1 years follow-up	1,638 /98,582	1.7%	2,097 /110,383	1.9%	0.93 (0.87, 1.00)
1-2 years follow-up	1,346 /93,758	1.4%	1,816 /105,021	1.7%	0.91 (0.84, 0.98)
2-3 years follow-up	1,243 /87,098	1.4%	1,597 /97,881	1.6%	0.93 (0.85, 1.00)
3-4 years follow-up	1,023 /73,197	1.4%	1,373 /82,864	1.7%	0.89 (0.81, 0.97)
4-5 years follow-up	703 /54,190	1.3%	836 /62,542	1.3%	0.98 (0.88, 1.10)
>5 years follow-up	503 /23,874	2.1%	627 /26,687	2.3%	0.91 (0.81, 1.03)
					P for trend = 0.684
All follow-up	6,456 /98,582	6.5%	8,346 /110,383	7.6%	0.92 (0.89, 0.95)
**Stroke**					
0-1 years follow-up	1,229 /98,585	1.2%	1,528 /110,374	1.4%	0.89 (0.82, 0.96)
1-2 years follow-up	894 /93,971	1.0%	1,145 /105,341	1.1%	0.83 (0.75, 0.91)
2-3 years follow-up	679 /87,641	0.8%	928 /98,712	0.9%	0.81 (0.73, 0.90)
3-4 years follow-up	555 /74,249	0.7%	731 /84,264	0.9%	0.84 (0.74, 0.95)
4-5 years follow-up	360 /55,602	0.6%	450 /64,356	0.7%	0.91 (0.79, 1.05)
>5 years follow-up	248 /24,891	1.0%	276 /27,997	1.0%	1.03 (0.86, 1.22)
					P for trend = 0.155
All follow-up	3,965 /98,585	4.0%	5,058 /110,374	4.6%	0.86 (0.83, 0.90)
**Heart failure**					
0-1 years follow-up	460 /91,009	0.5%	737 /102,898	0.7%	0.72 (0.62, 0.83)
1-2 years follow-up	447 /87,141	0.5%	570 /98,626	0.6%	0.92 (0.80, 1.06)
2-3 years follow-up	412 /81,326	0.5%	548 /92,605	0.6%	0.92 (0.80, 1.06)
3-4 years follow-up	398 /68,515	0.6%	512 /78,718	0.7%	0.90 (0.77, 1.05)
4-5 years follow-up	229 /51,149	0.4%	312 /60,126	0.5%	0.93 (0.75, 1.15)
>5 years follow-up	121 /21,815	0.6%	164 /25,037	0.7%	0.93 (0.73, 1.19)
					P for trend = 0.021
All follow-up	2,067 /91,009	2.3%	2,843 /102,898	2.8%	0.87 (0.81, 0.93)
**Cardiovascular death**					
0-1 years follow-up	672 /97,925	0.7%	808 /109,888	0.7%	0.90 (0.81, 1.01)
1-2 years follow-up	698 /94,322	0.7%	865 /106,127	0.8%	0.88 (0.79, 0.99)
2-3 years follow-up	698 /88,610	0.8%	866 /100,322	0.9%	0.94 (0.84, 1.05)
3-4 years follow-up	632 /75,477	0.8%	802 /86,226	0.9%	0.92 (0.82, 1.02)
4-5 years follow-up	432 /56,629	0.8%	577 /66,178	0.9%	0.90 (0.78, 1.03)
>5 years follow-up	303 /25,292	1.2%	400 /28,856	1.4%	0.89 (0.77, 1.04)
					P for trend = 0.710
All follow-up	3,435 /97,925	3.5%	4,318 /109,888	3.9%	0.91 (0.86, 0.95)
**All-cause death**					
0-1 years follow-up	1,467 /98,509	1.5%	1,773 /110,287	1.6%	0.93 (0.86, 1.00)
1-2 years follow-up	1,812 /94,909	1.9%	2,138 /106,533	2.0%	0.95 (0.89, 1.02)
2-3 years follow-up	1,841 /89,128	2.1%	2,326 /100,636	2.3%	0.92 (0.86, 0.99)
3-4 years follow-up	1,747 /75,867	2.3%	2,133 /86,425	2.5%	0.96 (0.89, 1.02)
4-5 years follow-up	1,212 /57,008	2.1%	1,454 /66,377	2.2%	1.01 (0.92, 1.10)
>5 years follow-up	825 /25,661	3.2%	931 /29,053	3.2%	1.00 (0.91, 1.10)
					P for trend = 0.101
All follow-up	8,904 /98,509	9.0%	10,755 /110,287	9.8%	0.96 (0.93, 0.99)

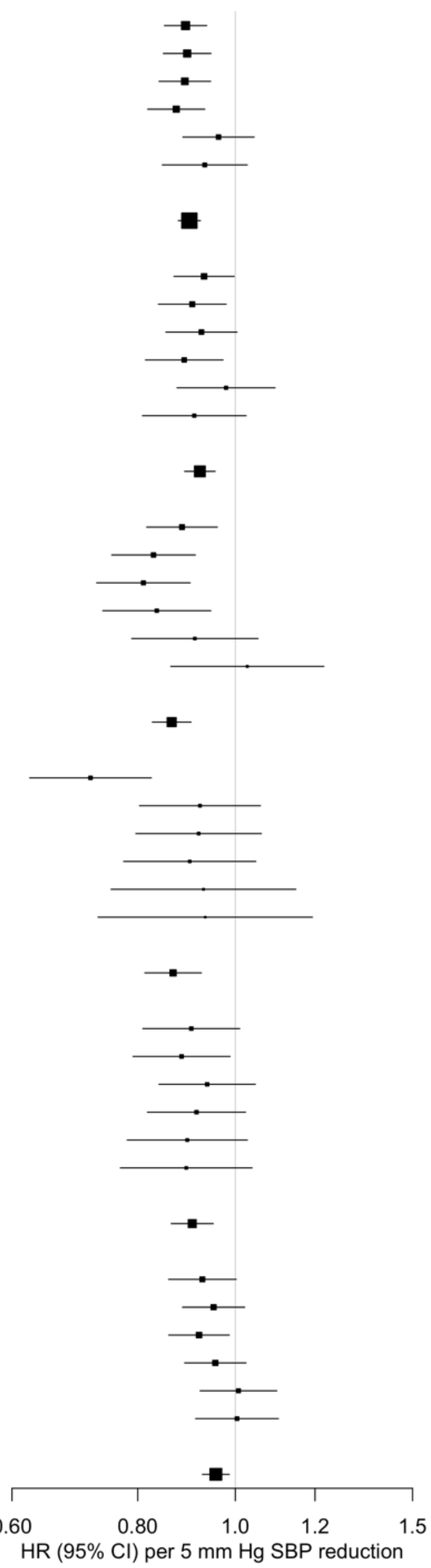

Supplementary Fig. 12. Standardised effects of systolic blood pressure treatment on outcomes among women, stratified by follow-up duration

Follow-up period	Treatment group (n/N)	Incidence rate (%)	Control group (n/N)	Incidence rate (%)	HR (95% CI) per 5 mm Hg SBP reduction
**Major cardiovascular events**					
0-1 years follow-up	1,756 /66,205	2.7%	2,386 /73,725	3.2%	0.85 (0.79, 0.90)
1-2 years follow-up	1,490 /62,084	2.4%	1,974 /68,817	2.9%	0.86 (0.81, 0.93)
2-3 years follow-up	1,321 /57,029	2.3%	1,514 /63,364	2.4%	1.03 (0.96, 1.12)
3-4 years follow-up	985 /47,598	2.1%	1,327 /52,805	2.5%	0.87 (0.79, 0.95)
4-5 years follow-up	736 /34,434	2.1%	884 /38,617	2.3%	0.98 (0.88, 1.08)
>5 years follow-up	466 /16,399	2.8%	546 /18,033	3.0%	0.94 (0.83, 1.07)
					P for trend = 0.014
All follow-up	6,754 /66,205	10.2%	8,631 /73,725	11.7%	0.90 (0.87, 0.93)
**Ischaemic heart disease**					
0-1 years follow-up	701 /66,193	1.1%	959 /73,735	1.3%	0.91 (0.82, 1.01)
1-2 years follow-up	631 /62,792	1.0%	873 /69,855	1.2%	0.89 (0.80, 0.99)
2-3 years follow-up	563 /58,198	1.0%	673 /65,020	1.0%	1.09 (0.96, 1.23)
3-4 years follow-up	433 /48,980	0.9%	604 /54,691	1.1%	0.87 (0.75, 0.99)
4-5 years follow-up	326 /35,717	0.9%	417 /40,462	1.0%	0.99 (0.84, 1.15)
>5 years follow-up	253 /17,062	1.5%	313 /19,019	1.6%	0.90 (0.76, 1.07)
					P for trend = 0.681
All follow-up	2,907 /66,193	4.4%	3,839 /73,735	5.2%	0.93 (0.89, 0.98)
**Stroke**					
0-1 years follow-up	703 /66,208	1.1%	919 /73,720	1.2%	0.84 (0.76, 0.93)
1-2 years follow-up	578 /62,768	0.9%	739 /69,857	1.1%	0.86 (0.77, 0.97)
2-3 years follow-up	471 /58,227	0.8%	565 /65,137	0.9%	0.93 (0.81, 1.05)
3-4 years follow-up	344 /49,175	0.7%	480 /54,971	0.9%	0.82 (0.71, 0.95)
4-5 years follow-up	288 /36,071	0.8%	367 /40,904	0.9%	0.90 (0.77, 1.05)
>5 years follow-up	177 /17,408	1.0%	218 /19,397	1.1%	0.95 (0.78, 1.17)
					P for trend = 0.416
All follow-up	2,561 /66,208	3.9%	3,288 /73,720	4.5%	0.87 (0.82, 0.92)
**Heart failure**					
0-1 years follow-up	304 /58,785	0.5%	502 /66,584	0.8%	0.72 (0.62, 0.84)
1-2 years follow-up	284 /55,715	0.5%	363 /63,110	0.6%	0.91 (0.76, 1.08)
2-3 years follow-up	270 /51,526	0.5%	352 /58,759	0.6%	0.93 (0.78, 1.10)
3-4 years follow-up	230 /42,985	0.5%	308 /49,099	0.6%	0.91 (0.75, 1.11)
4-5 years follow-up	151 /31,253	0.5%	216 /36,446	0.6%	0.86 (0.69, 1.08)
>5 years follow-up	97 /14,204	0.7%	99 /16,478	0.6%	1.15 (0.86, 1.53)
					P for trend = 0.007
All follow-up	1,336 /58,785	2.3%	1,840 /66,584	2.8%	0.87 (0.81, 0.94)
**Cardiovascular death**					
0-1 years follow-up	392 /65,442	0.6%	478 /73,142	0.7%	0.93 (0.81, 1.07)
1-2 years follow-up	418 /62,559	0.7%	545 /70,011	0.8%	0.90 (0.79, 1.03)
2-3 years follow-up	392 /58,516	0.7%	470 /65,894	0.7%	1.02 (0.89, 1.18)
3-4 years follow-up	360 /49,771	0.7%	450 /56,038	0.8%	0.96 (0.83, 1.12)
4-5 years follow-up	262 /36,581	0.7%	326 /41,906	0.8%	0.98 (0.83, 1.16)
>5 years follow-up	223 /17,618	1.3%	242 /19,978	1.2%	1.15 (0.95, 1.38)
					P for trend = 0.065
All follow-up	2,047 /65,442	3.1%	2,511 /73,142	3.4%	0.97 (0.91, 1.03)
**All-cause death**					
0-1 years follow-up	764 /66,046	1.2%	941 /73,532	1.3%	0.92 (0.83, 1.02)
1-2 years follow-up	1,000 /63,175	1.6%	1,190 /70,425	1.7%	0.99 (0.91, 1.09)
2-3 years follow-up	1,013 /59,018	1.7%	1,189 /66,181	1.8%	1.02 (0.93, 1.12)
3-4 years follow-up	897 /50,100	1.8%	1,101 /56,184	2.0%	0.97 (0.88, 1.07)
4-5 years follow-up	632 /36,905	1.7%	821 /42,053	2.0%	0.95 (0.85, 1.06)
>5 years follow-up	504 /17,938	2.8%	553 /20,123	2.7%	1.08 (0.95, 1.22)
					P for trend = 0.280
All follow-up	4,810 /66,046	7.3%	5,795 /73,532	7.9%	0.98 (0.94, 1.02)

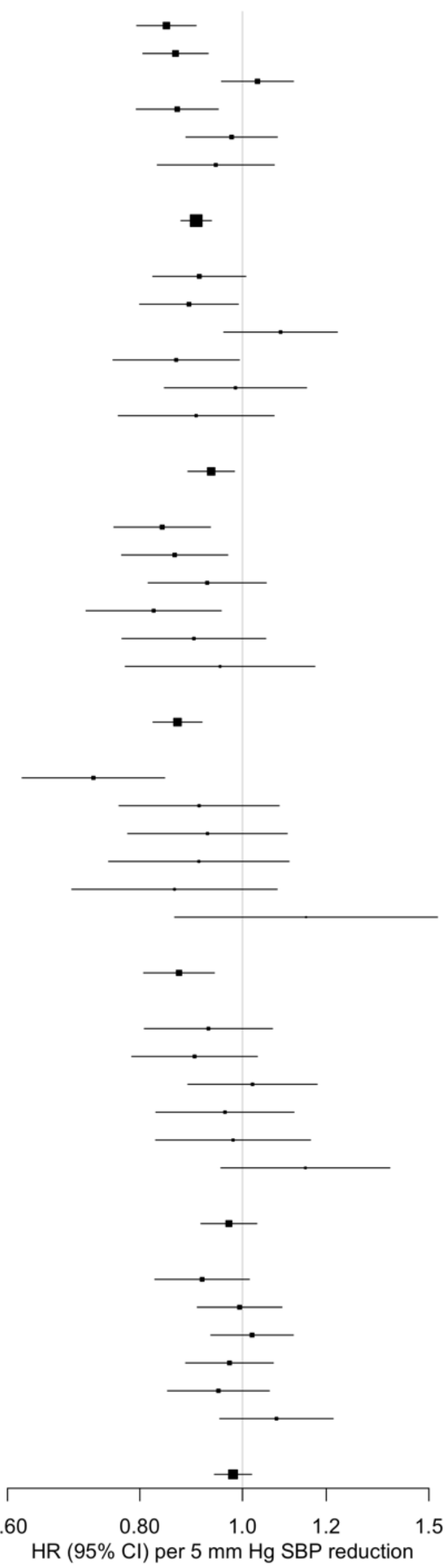

Q13. The study focusses on traditional cardiovascular outcomes (stroke, HF, IHD) whereas CKD has not been assessed. It could be interesting to see CKD as additional outcome.

Re: We agree that kidney outcomes are clinically important. However, we did not include chronic kidney disease (CKD) as an outcome in the present analysis because this study was specifically designed to evaluate traditional cardiovascular endpoints for two reasons:

First, blood pressure-related renal damage may operate through mechanisms and over different timescales that differ partly from the atherosclerotic and structural pathways underlying major cardiovascular events (PMID: 33637192).

Second, a robust pooled renal analysis would require harmonised kidney specific outcome definitions, such as sustained decline in estimated glomerular filtration rate, doubling of serum creatinine, or kidney failure, together with repeated kidney-function measurements (PMID: 33637192), which were not consistently available across all included trials. Instead, renal outcomes are the focus of a dedicated ongoing BPLTTC project designed specifically to examine CKD progression.

Q14. I would also like to understand if there are differences between those with vs without diabetes. From the adjusted models this doesn't seem to be the case but again, subgroup or sensitivity analyses could be provided.

Re: We have added subgroup analyses by baseline diabetes status. The temporal pattern was similar in participants with and without diabetes, with no evidence that treatment effects evolved differently over time by diabetes status (**Supplementary Figs. 13-14**).

Supplementary Fig. 13. Standardised effects of systolic blood pressure treatment on outcomes among participants with diabetes, stratified by follow-up duration

Follow-up period	Treatment group (n/N)	Incidence rate (%)	Control group (n/N)	Incidence rate (%)	HR (95% CI) per 5 mm Hg SBP reduction
**Major cardiovascular events**					
0-1 years follow-up	2,122 /46,772	4.5%	2,589 /53,075	4.9%	0.94 (0.88, 1.00)
1-2 years follow-up	1,742 /43,391	4.0%	2,169 /49,173	4.4%	0.91 (0.85, 0.98)
2-3 years follow-up	1,484 /39,537	3.8%	1,816 /45,077	4.0%	0.97 (0.89, 1.04)
3-4 years follow-up	1,233 /33,577	3.7%	1,564 /38,211	4.1%	0.90 (0.83, 0.97)
4-5 years follow-up	756 /25,443	3.0%	873 /29,236	3.0%	1.02 (0.92, 1.13)
>5 years follow-up	379 /9,973	3.8%	476 /11,315	4.2%	0.90 (0.78, 1.03)
					P for trend = 0.665
All follow-up	7,716 /46,772	16.5%	9,487 /53,075	17.9%	0.93 (0.90, 0.97)
**Ischaemic heart disease**					
0-1 years follow-up	948 /46,744	2.0%	1,130 /53,075	2.1%	1.03 (0.94, 1.13)
1-2 years follow-up	771 /44,202	1.7%	1,054 /50,302	2.1%	0.90 (0.81, 1.00)
2-3 years follow-up	716 /40,908	1.8%	889 /46,869	1.9%	1.01 (0.90, 1.13)
3-4 years follow-up	574 /35,192	1.6%	796 /40,334	2.0%	0.87 (0.77, 0.97)
4-5 years follow-up	430 /27,067	1.6%	503 /31,428	1.6%	1.07 (0.93, 1.22)
>5 years follow-up	262 /10,546	2.5%	327 /12,161	2.7%	0.91 (0.77, 1.07)
					P for trend = 0.475
All follow-up	3,701 /46,744	7.9%	4,699 /53,075	8.9%	0.96 (0.92, 1.01)
**Stroke**					
0-1 years follow-up	699 /46,756	1.5%	859 /53,052	1.6%	0.86 (0.76, 0.97)
1-2 years follow-up	535 /44,312	1.2%	659 /50,428	1.3%	0.87 (0.76, 0.99)
2-3 years follow-up	412 /41,145	1.0%	524 /47,269	1.1%	0.88 (0.76, 1.02)
3-4 years follow-up	346 /35,709	1.0%	451 /41,041	1.1%	0.84 (0.72, 0.99)
4-5 years follow-up	179 /27,726	0.6%	250 /32,340	0.8%	0.83 (0.67, 1.02)
>5 years follow-up	111 /11,185	1.0%	152 /12,897	1.2%	0.84 (0.65, 1.08)
					P for trend = 0.736
All follow-up	2,282 /46,756	4.9%	2,895 /53,052	5.5%	0.85 (0.80, 0.91)
**Heart failure**					
0-1 years follow-up	414 /44,969	0.9%	612 /51,591	1.2%	0.83 (0.70, 0.98)
1-2 years follow-up	387 /42,798	0.9%	481 /49,174	1.0%	0.96 (0.81, 1.14)
2-3 years follow-up	353 /39,786	0.9%	478 /46,168	1.0%	0.92 (0.79, 1.08)
3-4 years follow-up	332 /34,391	1.0%	425 /39,937	1.1%	0.93 (0.79, 1.09)
4-5 years follow-up	197 /26,564	0.7%	248 /31,347	0.8%	0.96 (0.77, 1.19)
>5 years follow-up	99 /10,173	1.0%	130 /12,144	1.1%	1.02 (0.78, 1.35)
					P for trend = 0.035
All follow-up	1,782 /44,969	4.0%	2,374 /51,591	4.6%	0.92 (0.85, 0.99)
**Cardiovascular death**					
0-1 years follow-up	475 /45,955	1.0%	478 /52,634	0.9%	1.08 (0.93, 1.25)
1-2 years follow-up	475 /44,057	1.1%	545 /50,701	1.1%	0.93 (0.79, 1.08)
2-3 years follow-up	432 /41,225	1.0%	530 /47,941	1.1%	0.95 (0.82, 1.11)
3-4 years follow-up	438 /35,884	1.2%	517 /41,852	1.2%	1.01 (0.87, 1.18)
4-5 years follow-up	253 /27,883	0.9%	364 /33,138	1.1%	0.82 (0.68, 0.98)
>5 years follow-up	185 /10,888	1.7%	213 /13,120	1.6%	1.13 (0.92, 1.38)
					P for trend = 0.330
All follow-up	2,258 /45,955	4.9%	2,647 /52,634	5.0%	0.98 (0.92, 1.05)
**All-cause death**					
0-1 years follow-up	873 /46,697	1.9%	1,022 /53,001	1.9%	0.95 (0.86, 1.07)
1-2 years follow-up	1,120 /44,794	2.5%	1,272 /51,064	2.5%	0.99 (0.89, 1.09)
2-3 years follow-up	1,118 /41,963	2.7%	1,337 /48,303	2.8%	0.98 (0.89, 1.08)
3-4 years follow-up	1,038 /36,613	2.8%	1,250 /42,215	3.0%	1.01 (0.91, 1.11)
4-5 years follow-up	691 /28,590	2.4%	899 /33,495	2.7%	0.94 (0.84, 1.05)
>5 years follow-up	468 /11,577	4.0%	497 /13,465	3.7%	1.07 (0.94, 1.22)
					P for trend = 0.736
All follow-up	5,308 /46,697	11.4%	6,277 /53,001	11.8%	0.99 (0.95, 1.03)

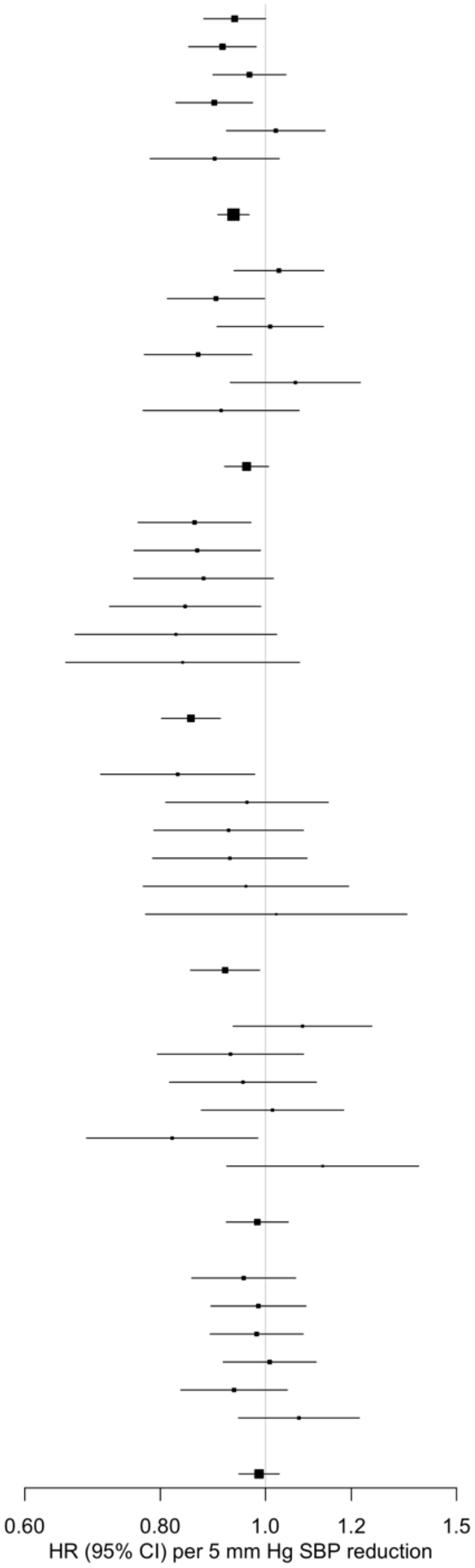

Supplementary Fig. 14. Standardised effects of systolic blood pressure treatment on outcomes among participants without diabetes, stratified by follow-up duration

Follow-up period	Treatment group (n/N)	Incidence rate (%)	Control group (n/N)	Incidence rate (%)	HR (95% CI) per 5 mm Hg SBP reduction
**Major cardiovascular events**					
0-1 years follow-up	3,014 /114,621	2.6%	4,123 /127,515	3.2%	0.84 (0.80, 0.88)
1-2 years follow-up	2,491 /107,859	2.3%	3,292 /119,534	2.8%	0.86 (0.82, 0.91)
2-3 years follow-up	2,158 /99,324	2.2%	2,674 /110,186	2.4%	0.92 (0.87, 0.98)
3-4 years follow-up	1,676 /83,384	2.0%	2,257 /92,861	2.4%	0.85 (0.79, 0.91)
4-5 years follow-up	1,235 /60,894	2.0%	1,499 /68,854	2.2%	0.94 (0.87, 1.02)
>5 years follow-up	842 /29,281	2.9%	992 /32,052	3.1%	0.96 (0.87, 1.05)
					P for trend = 0.001
All follow-up	11,416 /114,621	10.0%	14,837 /127,515	11.6%	0.88 (0.86, 0.90)
**Ischaemic heart disease**					
0-1 years follow-up	1,390 /114,646	1.2%	1,924 /127,546	1.5%	0.87 (0.81, 0.93)
1-2 years follow-up	1,204 /109,037	1.1%	1,632 /121,154	1.3%	0.90 (0.83, 0.97)
2-3 years follow-up	1,090 /101,156	1.1%	1,381 /112,692	1.2%	0.95 (0.87, 1.03)
3-4 years follow-up	882 /85,439	1.0%	1,181 /95,651	1.2%	0.89 (0.80, 0.98)
4-5 years follow-up	599 /62,683	1.0%	750 /71,445	1.0%	0.92 (0.81, 1.04)
>5 years follow-up	493 /30,358	1.6%	613 /33,519	1.8%	0.91 (0.80, 1.02)
					P for trend = 0.221
All follow-up	5,658 /114,646	4.9%	7,481 /127,546	5.9%	0.90 (0.87, 0.94)
**Stroke**					
0-1 years follow-up	1,231 /114,636	1.1%	1,587 /127,517	1.2%	0.86 (0.79, 0.93)
1-2 years follow-up	937 /109,100	0.9%	1,225 /121,322	1.0%	0.83 (0.76, 0.91)
2-3 years follow-up	738 /101,474	0.7%	969 /113,211	0.9%	0.84 (0.76, 0.93)
3-4 years follow-up	553 /86,156	0.6%	760 /96,604	0.8%	0.82 (0.73, 0.93)
4-5 years follow-up	469 /63,792	0.7%	566 /72,785	0.8%	0.95 (0.84, 1.07)
>5 years follow-up	314 /31,082	1.0%	342 /34,471	1.0%	1.05 (0.90, 1.23)
					P for trend = 0.025
All follow-up	4,242 /114,636	3.7%	5,449 /127,517	4.3%	0.86 (0.83, 0.90)
**Heart failure**					
0-1 years follow-up	350 /101,442	0.3%	627 /114,368	0.5%	0.66 (0.57, 0.76)
1-2 years follow-up	344 /96,748	0.4%	451 /109,112	0.4%	0.90 (0.78, 1.04)
2-3 years follow-up	329 /89,832	0.4%	422 /101,825	0.4%	0.93 (0.79, 1.08)
3-4 years follow-up	296 /75,563	0.4%	395 /86,296	0.5%	0.87 (0.73, 1.04)
4-5 years follow-up	183 /55,688	0.3%	280 /65,092	0.4%	0.83 (0.67, 1.02)
>5 years follow-up	118 /25,816	0.5%	133 /29,346	0.5%	1.06 (0.82, 1.36)
					P for trend = 0.004
All follow-up	1,620 /101,442	1.6%	2,308 /114,368	2.0%	0.83 (0.78, 0.89)
**Cardiovascular death**					
0-1 years follow-up	586 /113,964	0.5%	805 /126,828	0.6%	0.82 (0.73, 0.91)
1-2 years follow-up	636 /109,450	0.6%	864 /121,946	0.7%	0.84 (0.76, 0.94)
2-3 years follow-up	658 /102,607	0.6%	806 /114,863	0.7%	0.97 (0.88, 1.08)
3-4 years follow-up	554 /87,775	0.6%	734 /98,795	0.7%	0.89 (0.79, 1.00)
4-5 years follow-up	441 /65,169	0.7%	539 /74,808	0.7%	0.98 (0.86, 1.11)
>5 years follow-up	340 /31,990	1.1%	429 /35,687	1.2%	0.94 (0.82, 1.09)
					P for trend = 0.038
All follow-up	3,215 /113,964	2.8%	4,177 /126,828	3.3%	0.90 (0.85, 0.94)
**All-cause death**					
0-1 years follow-up	1,354 /114,410	1.2%	1,688 /127,250	1.3%	0.90 (0.84, 0.97)
1-2 years follow-up	1,686 /109,916	1.5%	2,053 /122,403	1.7%	0.95 (0.89, 1.02)
2-3 years follow-up	1,735 /102,889	1.7%	2,178 /115,102	1.9%	0.94 (0.88, 1.01)
3-4 years follow-up	1,606 /87,765	1.8%	1,983 /98,777	2.0%	0.94 (0.88, 1.01)
4-5 years follow-up	1,152 /65,165	1.8%	1,376 /74,797	1.8%	1.02 (0.93, 1.11)
>5 years follow-up	860 /31,990	2.7%	987 /35,684	2.8%	1.01 (0.92, 1.11)
					P for trend = 0.016
All follow-up	8,393 /114,410	7.3%	10,265 /127,250	8.1%	0.95 (0.92, 0.98)

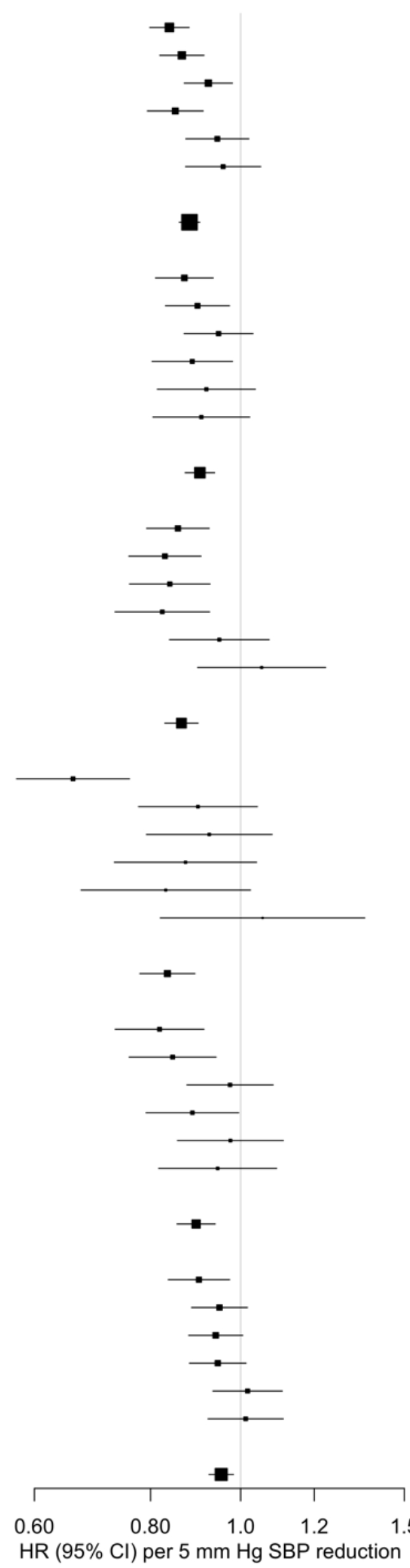

Q15. More than a third of participants were on lipid-lowering therapy. Is there any confounding by these powerful medications that also reduce CV risk? It is unlikely that e.g. statins would interact with the effect of blood pressure reduction on risk reduction but again a sensitivity analysis of participants who were not on lipid-lowering therapy could be provided.

Re: First, confounding by baseline lipid-lowering therapy should be limited because the included studies were large randomised trials and baseline use of lipid-lowering therapy was balanced by design between intervention and comparator groups within each trial. This substantially reduces the possibility that concomitant statin use affected the estimated relative treatment effects.

Second, there is no clear evidence that lipid-lowering therapy modifies the relative effect of blood pressure-lowering treatment. Large-scale randomised trials with factorial designs, including ASCOT Legacy Study (PMID: 30158072) and HOPE-3 (PMID: 27040132), as well as subsequent meta-analyses of their joint effects (PMID: 29595132), suggest that these interventions reduce cardiovascular risk in a largely independent and additive manner. Accordingly, a biologically important interaction between lipid-lowering therapy and blood pressure reduction appears unlikely in the context of our study.

Third, not all included trials systematically collected or reported detailed information on baseline lipid-lowering therapy. Restricting analyses to participants with complete data would therefore reduce the available sample size

and statistical power, particularly for the time-stratified analyses presented in this study.

That said, in response to the reviewer's suggestion, we conducted a sensitivity analysis restricted to participants who were not receiving lipid-lowering therapy, for which such data were available. The results were consistent with the overall findings. Specifically, relative risk reductions were established early after treatment initiation and did not show a progressively increasing benefit in major CVD prevention over time (**Figure R2)**.

Figure R2. Standardised effects of blood pressure-lowering treatment on major cardiovascular events across follow-up intervals, restricting to participants without use of lipid-lowering treatment at baseline

Follow-up period	Treatment group (n/N)	Incidence rate (%)	Control group (n/N)	Incidence rate (%)	HR (95% CI) per 5 mm Hg SBP reduction
0-1 years follow-up	1,268 / 47,224	2.7%	1,441 / 45,816	3.1%	0.84 (0.78, 0.91)
1-2 years follow-up	1,136 / 44,013	2.6%	1,313 / 42,623	3.1%	0.82 (0.75, 0.89)
2-3 years follow-up	976 / 40,616	2.4%	1,098 / 39,236	2.8%	0.87 (0.80, 0.96)
3-4 years follow-up	879 / 37,155	2.4%	1,003 / 35,827	2.8%	0.84 (0.76, 0.92)
4-5 years follow-up	571 / 28,223	2.0%	587 / 27,300	2.2%	0.93 (0.82, 1.07)
>5 years follow-up	202 / 12,461	1.6%	223 / 12,126	1.8%	0.93 (0.76, 1.15)
**All follow-up**	**5,032 / 47,224**	**10.7%**	**5,665 / 45,816**	**12.4%**	**0.85 (0.82, 0.89)**

P for trend = 0.357

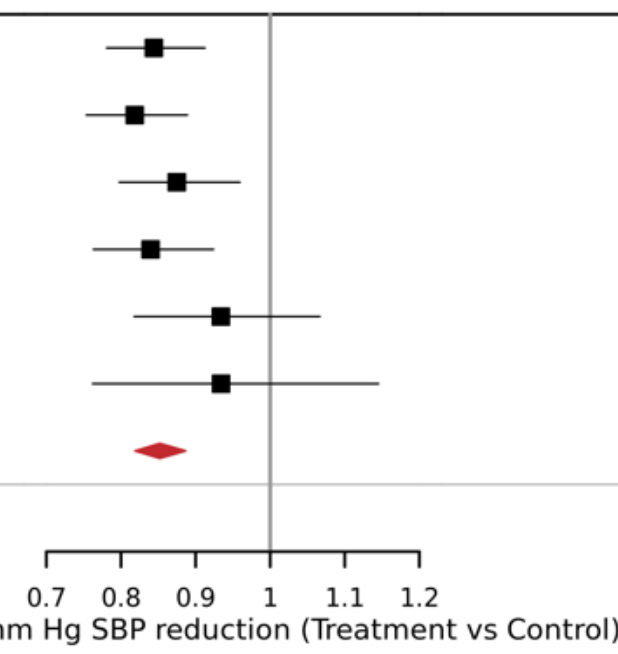

Q16. The analysis by drug classes is not entirely clear to me. Many participants will be on combination therapy either because of pre-existing therapy, add-on therapy or trial designs with combination therapy. Please elaborate on how the analysis by drug class was done.

Re: The network meta-analysis was conducted at the level of randomised drug-class allocation, following the intention-to-treat principle. The estimand was therefore the effect of being assigned to a given drug-class strategy within a trial, rather than the isolated pharmacological effect of strict monotherapy.

First, regarding background or add-on therapy, participants could receive background antihypertensive therapy at baseline or additional therapy during follow-up according to individual trial protocols. However, because treatment assignment was randomised, such co-interventions should, in principle, have been balanced across randomised groups within each trial and were therefore not modelled explicitly. Accordingly, the estimated effects reflect the overall effect of allocation to a given drug-class strategy, inclusive of any protocol-permitted background or add-on therapy.

Second, regarding trials involving combination therapy or non–drug-class comparisons, we excluded trial arms in which the randomised intervention consisted of a fixed combination (for example, ACE inhibitor plus calcium channel blocker in BENEDICT), because these could not be cleanly assigned to a single class. We also excluded trials in which the randomised comparison was treatment intensity rather than drug class (for example, intensive-versus-standard target

trials such as SPRINT and ACCORD). Trials with three or more arms were retained and analysed using a multi-arm Cox model with a common reference group, thereby preserving the within-trial randomisation structure.

To improve clarity, we have added the following statement to the Methods section:

"The network meta-analysis was conducted at the level of randomised drug-class allocation, following the intention-to-treat principle. Estimated effects, therefore, reflected assignment to a given drug-class strategy, including any protocol-permitted background or add-on therapy. Trial arms in which the randomised intervention consisted of fixed combination therapies were excluded to ensure that each comparison could be attributed to a single drug class."

Q17. A more general comment, and the authors acknowledge this in the limitations section, is that outcomes that will need more time to develop (e.g. cognitive impairment) will not be captured within the median follow-up time of 4 years. In fact, if lifecourse and lifetime risk play a prominent role for any outcome it is probably cognitive impairment – we just don't have robust data in this area yet.

Re: We have expanded the Limitations section accordingly:

“Third, the present analysis did not evaluate the effects of treatment on other outcomes, such as cognitive function and chronic kidney disease, over time, and further studies will be essential to clarify these patterns.”

Q18. Equally generally, the mean age of study participants wasn’t exactly on the young side. Conclusions about lifetime risk cannot be easily drawn from the data and this should be acknowledged. Plasticity of the cardiovascular system and effects to modify disease progression will presumably be different in a 30-year-old compared to a 65-year-old person.

Re: We have revised the Discussion to avoid extrapolating these findings to lifetime risk reduction and have explicitly acknowledged this limitation as follows:

“Fourth, because the mean age of the trial population was over 60 years, and even the youngest subgroup (<55 years old) represented midlife rather than early adulthood, these findings should not be extrapolated directly to treatment initiated in early adult life. However, to our knowledge, trials conducted in low-risk younger individuals are lacking.”

Q19. As far as I saw, the ASCOT Legacy trial was not mentioned. Wouldn’t line 410 provide an opportunity to do so?

Re: We have now incorporated this study into the Discussion as an example of extended follow-up after randomised treatment:

"For example, in the Anglo-Scandinavian Cardiac Outcomes Trial (ASCOT) Legacy study, participants originally assigned to the amlodipine-based regimen had lower long-term cardiovascular death.[21]"

Q20. The discussion on sensitivity analyses (lines 444 to 463) can be shortened as these analyses have been introduced in detail in the methods section and reported in the results section. The discussion doesn't go much deeper.

Re: We have removed this from the Discussion section and now retain only a summary in the limitations paragraph:

"However, the overall pattern was similar across sensitivity analyses and was not materially altered by alternative modelling of follow-up time, changes in cohort composition, attenuation of BP separation or declining adherence. These findings make it less likely that the absence of progressively stronger effects was explained solely by informative censoring."

Q21. Having read the paper in some detail I spotted a typo in line 34 (should read "a hypothesis") and the sentence in lines 244/345 will need revision/completion.

Re: We thank the reviewer for identifying these points. We have corrected them accordingly.

The corrected text now reads as follows.

Abstract:

“…a hypothesis…”

Line 244:

“To assess whether treatment effects changed across follow-up, we performed a meta-regression of the interval-specific treatment estimates derived from the IPD network meta-analysis, with the Knapp–Hartung adjustment applied.”

Line 345:

“Third, to examine the potential influence of declining treatment adherence over time, analyses were restricted to participants with high adherence (defined as taking at least 80% of the prescribed medication) across 12 trials with available adherence data.”

Point-by-point answer to the reviewer comments

Re: We thank the reviewers for their careful re-evaluation of our revised manuscript and for their constructive comments throughout the review process.

Reviewer #1:

Remarks to the Author:

Appreciate the authors' thoughtful and substantive revision that addresses the primary methodological concerns raised in my initial review. The revised manuscript now clearly acknowledges that the time stratified hazard ratios are conditional on evolving risk sets and that participants contributing to later follow up intervals represent a progressively selected, event free subset of the original randomized cohort. The expanded discussion of informative censoring, the explicit recognition of the limitations of IPCW in the presence of unmeasured dropout drivers, and more careful tempering of causal interpretation helps strengthen the framing of the results. The rigor and addition of complementary fixed horizon absolute risk analyses anchored to the original randomized population provides an important population level perspective that helps reconcile the stable or attenuated relative effects with accumulating absolute benefit over time. Still, residual survivor bias cannot be fully excluded, the authors' revised interpretation appropriately reflects this limitation and avoids over attribution of temporal patterns to treatment alone. Overall, the revisions have significantly improved the methodological transparency and interpretability of the manuscript, and my central concerns have been satisfactorily addressed.

Re: We thank you for your careful reassessment of the revised manuscript and for your positive comments. We are grateful that you found the revisions to have improved the manuscript.

Reviewer #2:

Remarks to the Author:

Many thanks to the authors for addressing the comments. No further comment

from my side.

Re: Thank you for reassessing the revised manuscript and for confirming that your previous comments have been addressed.

Reviewer #3:
Remarks to the Author:
Thank you. My comments have been addressed appropriately and the paper has further improved. I have no additional questions.

Re: We thank you for reassessing the revised manuscript and for your positive comments on the revised paper.
